# Antibacterial activity of the nitrovinylfuran G1 (Furvina) and its conversion products

**DOI:** 10.1038/srep36844

**Published:** 2016-11-10

**Authors:** Ülar Allas, Lauri Toom, Anastasia Selyutina, Uno Mäeorg, Ricardo Medina, Andres Merits, Ago Rinken, Vasili Hauryliuk, Niilo Kaldalu, Tanel Tenson

**Affiliations:** 1 University of Tartu, Institute of Technology, Nooruse 1, Tartu 50411, Estonia; 2 Institute of Chemistry, University of Tartu, Ravila 14a, Tartu 50411, Estonia; 3 Universidad Central “Marta Abreu” de Las Villas, Santa Clara, Cuba; 4 Department of Molecular Biology, Umeå University, Umeå, Sweden; 5 Laboratory for Molecular Infection Medicine Sweden, Umeå University, Umeå, Sweden

## Abstract

2-Bromo-5-(2-bromo-2-nitrovinyl)furan (G1 or Furvina) is an antimicrobial with a direct reactivity against thiol groups. It is active against Gram-positive and Gram-negative bacteria, yeasts and filamentous fungi. By reacting with thiol groups it causes direct damage to proteins but, as a result, is very short-living and interconverts into an array of reaction products. Our aim was to characterize thiol reactivity of G1 and its conversion products and establish how much of antimicrobial and cytotoxic effects are due to the primary activity of G1 and how much can be attributed to its reaction products. Stability of G1 in growth media as well as its conversion in the presence of thiols was characterized. The structures of G1 decomposition products were determined using NMR and mass-spectroscopy. Concentration- and time-dependent killing curves showed that G1 is bacteriostatic for *Escherichia coli* at the concentration of 16 μg/ml and bactericidal at 32 μg/ml. However, G1 is inefficient against non-growing *E. coli*. Addition of cysteine to medium reduces the antimicrobial potency of G1. Nevertheless, the reaction products of G1 and cysteine enabled prolonged antimicrobial action of the drug. Therefore, the activity of 2-bromo-5-(2-bromo-2-nitrovinyl)furan is a sum of its immediate reactivity and the antibacterial effects of the conversion products.

2-Bromo-5-(2-bromo-2-nitrovinyl)furan (G1 or Furvina) belongs to the group of 2-vinylfuran-based antimicrobials^1^. It is an efficient antimicrobial with broad-spectrum activity against Gram-positive and Gram-negative bacteria, yeasts and filamentous fungi^2^,^3^,^4^. G1 is currently in medical use in Cuba. It is marketed as Dermofural ointment for treatment of human skin and nail infections and as Furvinol for veterinary use. In Europe and USA, G1 is not in clinical use but patents for its synthesis and application as an anti-infective have been granted internationally^5^,^6^,^7^.

The exact details of action of G1 are a matter of debate. Just like other vinylfurans (2-furylethylenes), it is a thiolreactive compound and reacts with functional thiol groups in living cells, including those of vital enzymes^4^,^8^,^9^,^10^. The reaction center of vinylfurans in these addition reactions is the electrophilic exocyclic double bond^9^,^11^. For example, G1 has been shown to react with the cysteine residues of MurA, a protein that is crucial to peptidoglycan synthesis and is the target of phosphomycin^4^. Modification of cysteines is irreversible, supposedly rather nonselective and bactericidal, if it is extensive^4^. Due to the thiol reactivity, G1 must be short-lived both in a patient and during the standard tests of antimicrobial activity because such tests are carried out in cysteine-containing complex media. Besides adduct formation, G1 induces oxidation of thiol groups and formation of non-native disulfide bonds in a redox reaction. That leads to formation of a thiol-reactive de-bromo derivative of G1, which possesses antibacterial activity acting by the same mechanism as G1^4^. Furthermore, G1 is unstable in aqueous media even in the lack of thiols^4^. It breaks up into 5-bromo-2-furaldehyde, which does not have any antimicrobial activity, and bromonitromethane, that has been patented as a biocide while its target and the mechanism of action are unknown^4^,^12^,^13^.

Volatility of G1 evokes a question, how much of the reported antibacterial and cytotoxic effects are attributed to the compound itself and how much are due to its reaction- and breakdown products. Standard measurements of minimum inhibitory concentration (MIC), minimum bactericidal concentration (MBC), and cytotoxicity do not answer this question because all these tests are the endpoint measurements and score the compound’s effect at a single time point, usually 24–72 h after its application. In spite of its high reactivity, G1 has shown reasonable long-term effects in these tests^2^,^3^,^4^.

In this study, we wanted to clarify if the activity of G1 is lost shortly after application or the reaction products are active in a long term. We identified G1 reaction products and characterized interconversion of G1 in standard growth media. Thereafter, we assessed the antibacterial and cytotoxic effects using time-resolved assays. The experimental setup was based on a simple notion: we allowed G1 to react before we started susceptibility testing. Our study shows that the activity of G1 is partly attributed to its immediate reactivity and partly to the antibacterial effects of the reaction products.

## Materials and Methods

### Colorimetric measurements of reactivity and decomposition of G1

For measurements of conversion in culture media without addition of extra cysteine, G1 dissolved in DMSO was added to: cation-adjusted BBL Mueller-Hinton II Broth, (CAMHB) (Becton, Dickinson); M9 medium^14^ supplemented with glycine (Gly), L-isoleucine (Ile), L-phenylalanine (Phe), L-methionine (Met) and L-lysine monohydrochloride (Lys·HCl), each at a concentration of 100 μg/ml; and a mammalian cell culture medium (Iscove’s Modified Dulbecco’s Medium, 10% fetal calf serum, 1× Pen/Strep growth medium). A_390_ was recorded with an interval of 5 sec.

For measurements of the reactivity with cysteine, G1 dissolved in DMSO was added to M9 minimal media with different pH values to the final concentration of 16 μg/ml (0.054 mM). Aqueous solution of L-cysteine hydrochloride monohydrate (AppliChem) was added and A_390_ was recorded with an interval of 1 sec using Spectrophotometer Ultrospec 7000 (GE Healthcare Life Sciences).

### NMR spectroscopy and HPLC-HRMS analysis

The details of NMR spectroscopy as well as HPLC-HRMS analysis can be found in Supplemental material.

### Bacterial strains and growth conditions

*Escherichia coli* strain BW25113 (Δ*lacZ4787*(::*rrnB-3*), *hsdR514I*, ∆(*araD-araB*)*567* ∆(*rhaD-rhaB*)*568*, *rph-1*)^15^ and an uropathogenic strain CFT073 (O6:K2:H1)^16^ were used in all experiments and grown at 37 °C in Difco LB Broth, Lennox and on Difco LB Agar, Lennox (Becton, Dickinson) plates when not specified otherwise.

### Antibiotic susceptibility testing

MIC measurements were performed in accordance with the standard protocol^17^. In brief, G1 was dissolved in DMSO and stored at −20 °C. Working solutions of G1 were prepared by diluting G1 stock solution (5 mg/ml) in a testing medium. Testing media were: CAMHB supplemented with L-cysteine hydrochloride monohydrate (Cys·HCl·H_2_O) at a concentration of 100 μg/ml; M9 medium^14^ supplemented with 0.2% glucose and Gly, Ile, Phe, Met and Lys·HCl, each at a concentration of 100 μg/ml; and M9 medium supplemented with 0.2% glucose and Cys·HCl·H_2_O, Gly, Ile, Phe, Met and Lys·HCl, each at a concentration of 100 μg/ml. All the added amino acids were from Amresco, except Cys·HCl·H_2_O, which was from AppliChem. Two-fold serial dilutions were prepared using testing medium in 96-well plates. A growth control lacking G1 and a negative control containing only the testing medium were included. For inoculum preparation, fresh colonies were transferred into 3 ml LB broth and grown in a test tube overnight (approximately 16 h) at 37 °C with shaking. Overnight cultures were diluted 10-fold with LB broth and 3 ml volumes were incubated for 1 h at 37 °C with shaking. After 1 h resuscitation, bacteria were diluted in the testing medium 2000-fold to obtain final inoculum density of 2 × 10^4^ to 5 × 10^4^ CFU/ml. Inoculated microdilution plates were incubated at 37 °C for 12 h and 24 h. Growth was assessed measuring A_600_ using Tecan Sunrise plate reader. Yeast susceptibility tests were performed as suggested in National Committee for Clinical Laboratory Standards document M27-A2^18^.

### Determination of concentration- and time-dependent bactericidal activity

To test killing of bacteria in the logarithmic growth phase, overnight cultures were grown in 3 ml LB, diluted 1:1000 into 20 ml CAMHB and grown for 2.5 hours in 125 ml flasks at 37 °C with shaking. At this point, G1 was added at the concentrations of 8, 16, 32, and, 80 μg/ml. Samples were taken before addition of the drug and during a 5 hour time course after addition of the drug, serially diluted in CAMHB and drop-plated on LB agar.

To test killing of the stationary phase bacteria, overnight cultures were diluted 1:1000 into CAMHB and grown into stationary phase. After 12 hours at 37 °C with shaking, the cells were collected by centrifugation at 5000 g for 10 min. Conditioned CAMHB medium from the culture was filter-sterilized and used for resuspension and dilution of the bacteria. Bacteria were resuspended in a volume that was equal to the volume of the culture and diluted 1:10, 1:100, and 1:1000 in the conditioned medium. DMSO-dissolved G1 was added to the undiluted suspension and to all of the dilutions at the concentrations of 8, 32, and, 80 μg/ml. Bacteria were incubated for 5 hours at 37 °C with shaking. Samples were taken before addition of the drug and after the 5 hour incubation, serially diluted in fresh CAMHB medium and drop-plated on LB agar.

The number of viable bacteria was determined by counting colonies after 20 h of incubation at 37 °C. The limit of detection was 100 CFU/ml. The experiments were done in triplicates.

### Evaluation of cytotoxcicity in mammalian culture

The mammalian cytotoxicity was assayed using the xCELLigence RTCA DP Instrument (Roche). Different concentrations of DMSO-dissolved G1 were pre-incubated in media for 5 minutes. Then, 150 μl of U2OS human bone osteosarcoma epithelial cells or HeLa cells were combined with 50 μl dilutions of G1, seeded on E-plate 16 with integrated microelectrodes (3.5 × 10^3^ of cells, 150 μl of Iscove’s Modified Dulbecco’s Medium (IMDM), 10% fetal calf serum (FCS), 1× Pen/Strep growth medium) and incubated at 37 °C, 5% CO_2_ for 27 hours. The amount of viable cells was determined by measuring the electric impendence. Cell cultures contained 0.5% final concentration of DMSO. The experiments were done in duplicates.

## Results

### Stability and reactivity of G1 in microbial- and cell culture-media

**Figure 1:**
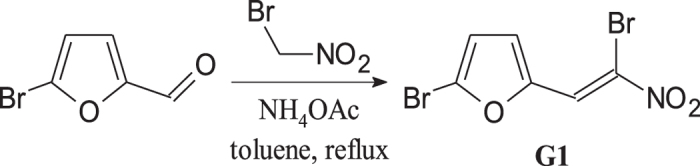
Synthesis of 2-bromo-5-(2-bromo-2-nitrovinyl)furan (G1).

G1 was synthesized according to the scheme of Scholz and colleagues^4^ with minor modifications (Fig. 1; detailed description in Supplemental material). The chemical purity of the compound was confirmed by TLC and NMR.

**Figure 2:**
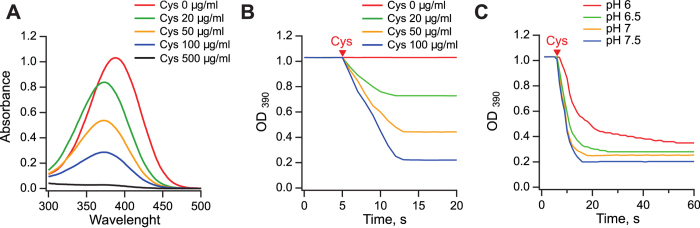
Colorimetric measurement of G1 reactivity with cysteine. Absorbance spectra of G1 and its reaction products obtained at different concentrations of cysteine hydrochloride (**A**). G1 at the concentration of 16 μg/ml (0.054 mM) was added 5 seconds after the start of the A_390_ measurement and reacted with cysteine hydrochloride in M9 minimal medium. Measurements were carried out at pH 7.4 using different concentrations of cysteine (**B**) and at different pH values in the presence of 100 μg/ml cysteine hydrochloride (**C**).

Conversion of vinylfurans, including G1, can be measured spectrophotometrically. Many vinylfurans have adsorption maxima in the 310–380 nm region in buffered aqueous solutions, while the adsorption maxima of the decomposition and reaction products are significantly lower and are shifted towards lower wavelengths^8^,^9^. G1 has the adsorption maximum at 390 nm (Fig. 2A). To estimate the time required for full conversion of G1 during antimicrobial assays, we measured drop of absorbance at this wavelength in different assay media applying G1 at concentrations that we used in biological assays. Among these media, M9 minimal medium with a supplement of 5 amino acids is free of sulfhydryl groups, whereas the bacterial complete medium CAMHB and mammalian cell culture medium (IMDM + FCS) contain unknown concentrations of thiols. Conversion of G1 in M9 medium and IMDM followed exponential decay with half-lives of 58 minutes and 11 minutes, respectively (Fig. S1A in the Supplemental material). The kinetics of decay in CAMHB was more complex, but followed approximately exponential decay during the first 20 minutes of incubation; the half-life was approximately 6 min (Fig. S1B in the Supplemental material).

To follow reaction with cysteine, we dissolved G1 in minimal M9 medium (16 μg/ml, 0.054 mM) and added cysteine hydrochloride monohydrate to final concentrations of 20 μg/ml (0.114 mM), 50 μg/ml (0.285 mM), 100 μg/ml (0.569 mM) and 500 μg/ml (2.85 mM). In the presence of excess of thiol, the reaction reached a maximum extent in less than 10 seconds and displayed a pseudo-first order with no effect of the cysteine concentration on the initial slope (Fig. 2B). The absorbance maximum of the reaction mixtures shifted from 390 nm to 372 nm (Fig. 2A). However, a large excess of cysteine was required for the absorbance maximum between 350–400 nm to disappear, indicating that cystine formation is greatly enhanced in the reaction with G1 in M9 medium. In addition to UV-visible absorbance measurements, this behavior was also followed by using ^1^H NMR spectroscopy. NMR spectra also supported the findings that cysteine is converted rapidly to cystine, thus the cysteine amount needed to react with the G1 or its reaction products was not sufficient even at 100 μg/ml initial cysteine concentration. It was also found that the fast-forming products of G1 were slowly decomposing to 5-bromo-2-furaldehyde. Thus, G1 reacts very rapidly with reduced cysteine and the intermediate product(s) of this reaction seemingly catalyze the cysteine-to-cystine conversion.

Previous studies of the vinylfuran reactivity have demonstrated that, in the presence of constant initial concentration of thiol, the reaction rate increases with pH value of the reaction mixture^8^. We prepared a series of M9 media with different pH values and tested dependence of G1 reactivity on pH of the medium. The reaction mixtures contained 100 μg/ml (0.569 mM) cysteine hydrochloride monohydrate, G1 was added at the concentration of 16 μg/ml (0.054 mM) and discoloration was measured at 390 nm. As seen in Fig. 2C, reaction is indeed slower at a lower pH and reaches to different plateau levels at different pH values.

We hypothesized that these different plateaus that are formed at various cysteine concentrations (Fig. 2B) and pH values (Fig. 2C) may correspond to different reaction products or mixtures of those.

### Identification of the reaction products of G1

**Figure 3:**
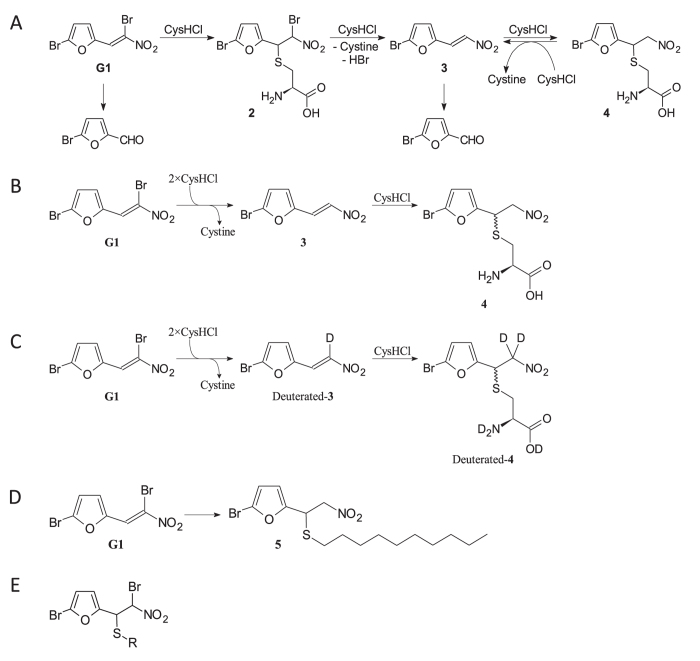
Reaction products of G1. (**A**) Structures of conversion products of G1 as detected using HPLC-HRMS analysis. (**B**) Reaction of G1 (1) with cysteine in CAMHB. (**C**) Reaction of G1 with cysteine in D_2_O/CD_3_OH (1:1). (**D**) Reaction of G1 with 1-decanethiol. (**E**) Intermediate Cys adduct (2) proposed by Scholz and coworkers^4^.

We determined and characterized the exact structure of the reaction products of G1 using NMR spectroscopy (Fig. 3, experimental details and NMR spectra in the Supplemental material). At pH > 6 and excess of cysteine the reaction was stepwise and proceeded over a previously reported intermediate product 2-bromo-5-(-2-nitroethenyl)furan **3**, where one bromine atom had been substituted with hydrogen atom^4^. At least three equivalents of cysteine were required to convert G1 to the product **4**. In the presence of remaining cysteine the product **4** was transforming back into compound **3**, giving cystine (unreactive product in the reaction scheme) and looping the **3**-to-**4** conversion. In the course of hours compound **3** was found to decompose irreversibly to 5-bromo-2-furaldehyde.

The product **4** was obtained regardless which co-solvent was used in the reaction. When a protic deuterated solvent (for example D_2_O or CD_3_OD) was used in the reaction, a partly deuterated form of the product **4** was obtained (Fig. 3C). The deuterated product formation can be explained by H/D exchange between the solvent and cysteine’s sulfhydryl group, which then undergoes a thiol-ene reaction. This deuterated product was also forming when the non-deuterated **4** was dissolved in such protic deuterated solvent, indicating that the α-hydrogens next to the nitro-group are relatively acidic to undergo H/D exchange. Using CD_3_CN-H_2_O 1:1 mixture as the solvent for the NMR studies was beneficial for determination of all the NMR signals of the compound **4** as the water signal was not overlapping with any of the other signals, also H/D exchange was not an issue, which had resulted in disappearance of several NMR signals when other solvents (CD_3_OD, D_2_O) were used.

Product **4** was obtained as 1:1 diastereoisomeric mixture due to a new chiral center. When using an aqueous bacterial growth medium CAMHB for studying the reaction between G1 and cysteine, same intermediate **3** and final product **4** were identified. Using just a small amount of **G1** (15 μg) was sufficient to produce clearly detectable ^1^H NMR signals of the product **4**. Although most of the product **4**^1^H NMR signals were overlapping with the signals from the CAMHB medium, the characteristic furane-ring hydrogens’ signals were well-separated from all other signals (see Supplemental material).

When cysteine hydrochloride was not added to CAMHB aqueous solution containing small amount of **G1** (10 μg), no reaction occurred according to ^1^H NMR spectra – there were no detectable signals for compounds **3** or **4** or their analogues, indicating that the amount of sulfhydryl groups in CAMHB medium is very low.

For comparison, **G1** was reacted with 1-decanethiol instead of cysteine, giving an analogous product **5** (Fig. 3D). This time the product **5** only has one chiral center, thus the product is just a mixture of two enantiomers giving exactly the same NMR signals. Apart from the product **5**′s thiol-part NMR signals, the G1-side signals were similar to the compound **4** signals, indicating that the NMR signals of furane-ring hydrogens (between 6.25 and 6.45 ppm) can be used as characteristic references to identify the analogues in more complex mixtures.

Scholz *et al*. proposed that conversion of G1 into compound **3** occurs over intermediate **2** (Fig. 3E). However, our NMR analysis did not reveal the presence of intermediate **2**, which may indicate that this intermediate is short-lived under used conditions. We therefore attempted to study the reactivity of G1 towards cysteine by HPLC-HRMS analysis. We reacted 10 μl of G1 stock solution (10 mM in DMSO) with 10 μl of cysteine hydrochloride stock solution (10 mM in H_2_O) in 980 μl sodium citrate buffer (50 mM, pH 6.0). Slightly acidic conditions were chosen as the reaction occurs slower at pH < 7 as seen in Fig. 2C, which in turn could increase the chances to detect intermediate **2**. Aliquots of the reaction mixture were sampled after every 60 s over a period of 7 min and the reaction in these aliquots was stopped immediately by quenching with orthophosphoric acid (85%). Indeed, HPLC analysis was able to detect the presence of intermediate **2** in stopped reaction aliquots together with G1 and compounds **3** and **4** (Fig. 1 in Supplemental material). Then, we collected HPLC fractions corresponding to compounds of interest and analyzed their contents by HRMS. The results obtained by HRMS help to interpret the results presented in Fig. 2A. The peak with maximum absorbance at 372 nm appears to belong to compound **3**. Compounds **2** and **4** cannot be detected in our colorimetric measurements as their corresponding absorption maxima are in ultraviolet wavelengths. The maximum values of absorbance in colorimetric measurements were slightly different from those in HRMS analysis which is in our opinion caused by different solvent systems (compare Fig. 2A and figures in pages S9 and S10 in Supplemental material).

### Bacterial growth inhibition by G1 and its conversion products

In order to make a distinction between the antibacterial properties of G1 and its conversion products, we supplemented G1 to different growth media and scored MIC after preincubation of these mixtures for different time periods prior to inoculation. Only minor differences between two *E. coli* strains were observable in our test (Table 1). As expected, we saw that in a minimal medium that is devoid of cysteine, G1 has much lower MIC when compared to complex medium (CAMHB). Preincubation of G1 for an hour in the growth media resulted in two-fold loss of activity. Addition of cysteine hydrochloride reduced considerably the antimicrobial potency of G1 in both CAMHB and M9 medium. However, reaction with cysteine does not render the compound inactive, indicating that the reaction products have antimicrobial properties. Scoring MIC after 24 h gave consistently higher readings then after 12 h (≈2× effect), most likely due to conversion of the most active compounds and bacterial re-growth. Similar results were also obtained in case of yeast *Candida albicans* (Table 2).

### Bactericidal activity of G1 and its conversion products

**Figure 4:**
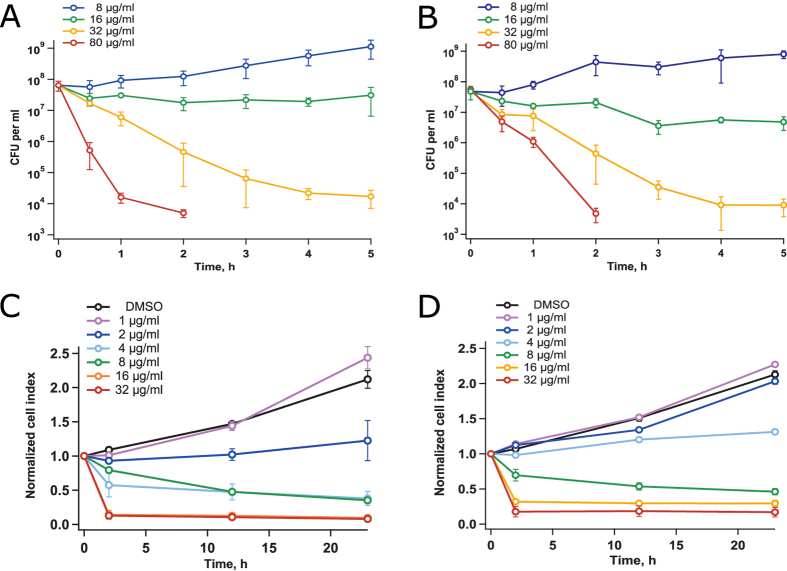
Toxicity of G1 against bacterial and mammalian cells as a function of incubation time. Effect of G1 on *E. coli* strains CFT073 (panel A) and BW25113 (panel B) was performed in CAMHB medium and determined by colony counting. Cytotoxicity against U2OS human bone osteosarcoma epithelial cells (panel C) and HeLa cells (panel D) was determined using xCELLigence RTCA DP Instrument. All experiments were repeated at least three times. The error bars represent standard deviation.

It is principally thinkable that G1 kills all inoculated bacteria and the results of antimicrobial susceptibility testing, which are interpreted in terms of growth inhibition, actually reflect sterilization. In order to delineate the bacteriostatic and bactericidal effects of G1 we performed a time-dependent killing experiment with different concentrations of the drug in CAMHB. When added to a log phase aerated culture, G1 at the concentration of 8 μg/ml slowed down but did not completely stop growth. At the concentration of 16 μg/ml, the drug was bacteriostatic and at 32 μg/ml it killed about 2.5 logs of bacteria during first 3 hours of incubation. At 80 μg/ml, the 3 h treatment sterilized the culture meaning that the number of surviving bacteria dropped below the limit of detection (100 CFU/ml). There was no remarkable difference between the behavior of two studied *E. coli* strains (Fig. 4A,B).

**Figure 5:**
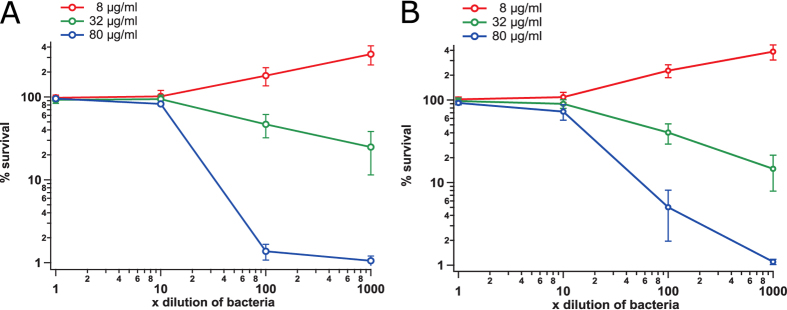
Survival of G1-treated stationary phase *E. coli* strains CFT073 (panel A) and BW25113 (panel B) as a function of cell density. Experiments were performed in CAMHB medium and percent of survival was determined by colony counting. The experiments were repeated at least three times and the error bars represent standard deviation.

Antimicrobial efficiency depends a lot on the physiological state, particularly on the growth phase and culture density, of the targeted microbe^19^,^20^. Since activity of G1 can be attributed to its rather nonselective chemical reactivity, we wanted to test if this compound is efficient against non-growing stationary phase *E. coli* that defies most antibiotics^21^. Killing of the non-growing bacteria was considerably less efficient than killing of growing *E. coli*, and occurred only at the highest tested concentration (80 μg/ml) of the drug (Fig. 5). Moreover, killing of the stationary phase bacteria required at least 100-fold dilution (to 10^7^ CFU/ml) of the culture. At a concentration of 32 μg/ml, the compound was bactericidal against the proliferating culture but remained bacteriostatic against non-growing bacteria. Non-growing BW25113 and uropathogenic CFT073 strains did not reveal any significant difference in their susceptibility to G1. In conclusion, we can say that G1 is inefficient against non-growing *E. coli*.

### Cytotoxic effect of G1 and its conversion products

In previous publications, cytotoxicity of G1 was scored after 72 h-long incubation^4^. To distinguish between the immediate cytotoxicity of G1 and the toxic effects of its conversion products, we tested cytotoxicity of G1 on cell cultures in a time-dependent manner using xCELLigence RTCA DP Instrument. U2OS human bone osteosarcoma epithelial cells and HeLa cells were included to the test (Fig. 4C,D). When G1 was applied at the concentrations of 1 μg/ml and 2 μg/ml, the growth was initially inhibited but the cells recovered later and were capable of regrowth. When the drug was present at concentrations of 8 μg/ml, 16 μg/ml and 32 μg/ml, cells could not overcome the toxic effect. Measurements at the concentrations above 32 μg/ml were impossible since G1 precipitated in the cell culture medium. Thus, when tested in thiol-containing media, G1 and its conversion products had pronounced long-term effects against the cultured mammalian cells.

## Discussion

Although G1 is used in human therapy, our understanding of the mode of action of the drug is incomplete. For example, authors of a recent publication have proposed that the compound inhibits bacterial cells via binding to the P site of the 30S ribosomal subunit and interfering with translation initiation^3^. That is definitely true *in vitro*, as the authors show, but based on multiple reports and our current experimental findings, activity of vinylfurans, including G1, is based on rather non-specific modification of thiol groups^1^,^4^,^8^. It has to be noted that although many proteins in the cell could be modified by the thiol specific reactions, the result is not always protein inactivation but sometimes rather specific change in properties. For example, modified ribosomes are still able to synthesize proteins but can translocate without EF-G^22^,^23^.

G1 transforms rapidly into an array of compounds with diverse half-lives and biological properties^4^. It is not self-evident, how such short-lived compound can retain any long-term activity. Using pre-incubation of the drug in thiol-containing media and time resolved assays we demonstrate that conversion products of G1 display bacteriostatic and bactericidal effects which are dependent on the composition of the medium as well as the cell density and growth phase of bacteria. The products of reaction between G1 and thiols (Cys·HCl) were identified and characterized by both NMR spectroscopy and HPLC-HRMS analysis. In agreement with the study by Scholz and colleagues, an adduct molecule of G1 (compound **2**) forms during reaction (Fig. 3A). This intermediate product is unstable and reacts quickly with next thiol-containing molecule. As a result, 2-bromo-5-(2-nitrovinyl)furan (compound **3**) is formed. Compound **3** reacts further to produce a covalently bonded cysteine conjugate **4** (Fig. 3A–C). Our study shows that the reaction products of G1 inhibit bacterial growth. Compound **3** is a thiol-reactive molecule and is, most likely, the main antibacterial and cytotoxic conversion product in thiol-containing environments. Immediately after G1 is applied, it firstly causes rapid oxidation of reduced cysteine. That leads to formation of non-native disulfide bonds and is a part of its antibacterial and cytotoxic effect. The de-bromo derivative (compound **3**) forms as a result of the redox reaction, and will further react with sulfhydryl groups to form adducts (compound **4**). In the presence of reduced cysteine, the product **4** is oxidizing it to cystine similarly to intermediate **2** and transforming back to compound **3**. Thanks to looping of the the **3**-to-**4** conversion, one molecule of G1 can react with more thiol groups in thiol-rich environment. Irreversible decomposition of compound **3** to 5-bromo-2-furaldehyde, which lacks antibacterial and biocidal activity, terminates this cycling reactivity.

In media that are poor in cysteine, e.g. assay in M9 minimal medium, decomposition of G1 produces bromonitromethane as an end product with biocidal activity^4^. Thus, we can conclude that the full effect of G1 is a sum of its immediate reactivity and effects of its conversion products. In spite of its indiscriminate mechanism, activity of the drug depends on bacterial growth and G1 is ineffective against non-growing microbes.

## Additional Information

**How to cite this article**: Allas, Ü. *et al*. Antibacterial activity of the nitrovinylfuran G1 (Furvina) and its conversion products. *Sci. Rep*. **6**, 36844; doi: 10.1038/srep36844 (2016).

**Publisher's note:** Springer Nature remains neutral with regard to jurisdictional claims in published maps and institutional affiliations.

## Supplementary Material

Supplementary Information

## Figures and Tables

**Table 1:** Effects of pre-incubation and MIC scoring time on antibacterial potency of G1 as determined in *E. coli* strains BW25113 and CTF073.

Medium	Preincubation without bacteria (min)	MIC (μg/ml)			
		Scored after 12 h		Scored after 24 h	
		BW25113	CFT073	BW25113	CFT073
MHB	5	8	8	8	8
	60	16	16	32	32
	120	16	16	32	32
MHB + Cys*	5	16	16	16	16
	60	32	32	>32	>32
	120	32	32	>32	>32
M9**	5	1	1	2	2
	60	2	2	4	4
	120	2	2	4	4
M9** + Cys*	5	8	4	16	8
	60	16	8	32	16
	120	16	16	32	32

^*^Supplemented with Cys·HCl·H_2_O at 100 μg/ml.

^**^M9 media supplemented with Phe, Ile, Gly, Lys·HCl and Met at 100 μg/ml.

**Table 2:** Effects of pre-incubation and MIC scoring time on toxicity of G1 for *Candida albicans* (strain CBS562^NT^).

Medium	Preincubation without bacteria (min)	MIC (μg/ml)	
		Scored after 48 h	Scored after 72 h
RPMI 1640	5	2	4
	60	4	16
	120	4	16
