# Supplementary material for: Antibacterial activity of the nitrovinylfuran G1 (Furvina) and its conversion products
Source: Sci Rep. 2016 Nov 10;6:36844. doi: 10.1038/srep36844 (PMC5103279; doi:10.1038/srep36844)
Supplement: Supplementary Information [file srep36844-s1.pdf]

Supporting information

Antibacterial activity of the nitrovinylfuran G1 (Furvina) and its conversion products

Ülar Allas[1], Lauri Toom[1], Anastasia Selyutina[1], Uno Mäeorg[2], Ricardo Medina[3], Andres Merits[1], Ago Rinken[2], Vasili Hauryliuk[1,4,5], Niilo Kaldalu[1], Tanel Tenson[1,α]

[1] University of Tartu, Institute of Technology, Nooruse 1, Tartu 50411, Estonia

[2] Institute of Chemistry, University of Tartu, Ravila 14a, Tartu 50411, Estonia

[3] Universidad Central "Marta Abreu" de Las Villas, Santa Clara, Cuba

[4] Department of Molecular Biology, Umeå University, Umeå, Sweden

[5] Laboratory for Molecular Infection Medicine Sweden, Umeå University, Umeå, Sweden

[α] corresponding author: tanel.tenson@ut.ee, +372 737 4844

Table of contents

General details of the experimental part 5

Synthetic procedure 6

2-Bromo-5-(2-bromo-2-nitroethenyl)furan (Furvina or G1) 6

Identification of the conversion products of G1 7

HPLC-HRMS analysis of reactivity of G1 towards cysteine 7

Ultraviolet-visible spectra of compounds G1, 2, 3 and 4 9

HRMS spectrum of 2 11

HRMS spectrum of 4 12

2-Bromo-5-(2-nitroethenyl)furan (3) 13

2-Bromo-5-[2-nitro(2-^{2}H)ethenyl]furan 13

Diastereoisomeric 1:1 mixture of *S*-[(1*R*)-1-(5-bromofuran-2-yl)-2-nitroethyl]-*L*-cysteine and *S*-[(1*S*)-1-(5-bromofuran-2-yl)-2-nitroethyl]-*L*-cysteine (4a and 4b) 14

Diastereoisomeric 1:1 mixture of *S*-[(1*R*)-1-(5-bromofuran-2-yl)-2-nitro(2,2-$^2H_2$)ethyl]-*L*-(*N*,*N*,*O*-$^2H_3$)cysteine and *S*-[(1*S*)-1-(5-bromofuran-2-yl)-2-nitro(2,2-$^2H_2$)ethyl]-*L*-(*N*,*N*,*O*-$^2H_3$)cysteine 15

2-Bromo-5-[1-(decylsulfanyl)-2-nitroethyl]furan (5) 15

NMR spectra and analytical data 16

Spectra of 2-bromo-5-(2-bromo-2-nitroethenyl)furan (G1) solution in $CDCl_3$ 17

^{1}H NMR spectrum (700.1 MHz) of G1 in $CDCl_3$ 18

^{13}C{^{1}H} and DEPT-135 NMR spectra (176.0 MHz) of G1 in $CDCl_3$ 19

^{13}C{^{1}H} and DEPT-135 NMR spectra (176.0 MHz) of G1 in $CDCl_3$ – expansion from +113.0 ppm to +152.0 ppm 20

^{1}H-^{15}N HMBC NMR spectrum (700.1 MHz, 70.9 MHz) of G1 in $CDCl_3$ 21

Stacked ^{1}H NMR spectra: titration of G1 with CysHCl in the presence of $NaHCO_3$ in CD_3CN/H_2O (1:1) 22

^{1}H NMR spectra (700.1 MHz): titration of G1 with CysHCl in the presence of $NaHCO_3$ in CD_3CN/H_2O (1:1) – expansion from -0.5 ppm to +11 ppm 23

^{1}H NMR spectra (700.1 MHz): titration of G1 with CysHCl in the presence of $NaHCO_3$ in CD_3CN/H_2O (1:1) – expansion from +6.2 ppm to +8.7 ppm 24

^{1}H NMR spectra (700.1 MHz): titration of G1 with CysHCl in the presence of $NaHCO_3$ in CD_3CN/H_2O (1:1) – expansion from +2.8 ppm to +5.1 ppm 25

Stacked ^{1}H NMR spectra: addition of G1 to MHB solution containing CysHCl in H_2O/D_2O (5:1)...... 26

^{1}H NMR spectra (700.1 MHz): addition of G1 solution to aq. cysteine solution in aq. MHB solution – expansion from -0.5 ppm to +11 ppm 27

^{1}H NMR spectra (700.1 MHz): addition of G1 solution to aq. cysteine solution in aq. MHB solution – expansion from -0.5 ppm to +11 ppm, vertical scale ×8 28

^{1}H NMR spectra (700.1 MHz): addition of G1 solution to aq. cysteine solution in aq. MHB solution – expansion from +6.1 ppm to +9.4 ppm, vertical scale ×32 29

^{1}H NMR spectra (700.1 MHz): addition of G1 solution to aq. cysteine solution in aq. MHB solution – Expansion from +6.1 ppm to +6.8 ppm, vertical scale ×32 30

^{1}H NMR spectra (700.1 MHz): addition of G1 solution to aq. cysteine solution in aq. MHB solution – expansion from +0.5 ppm to +5.5 ppm, vertical scale ×1 31

Stacked ^{1}H NMR spectra: addition of CysHCl to MHB solution containing G1 in H_2O/D_2O (1:1)...... 32

^{1}H NMR spectra (700.1 MHz): addition of CysHCl to MHB solution containing G1 in H_2O/D_2O (1:1) – expansion from -0.5 ppm to +11.0 ppm 33

^{1}H NMR spectra (700.1 MHz): addition of CysHCl to MHB solution containing G1 in H_2O/D_2O (1:1) – expansion from 6.1 ppm to 9.4 ppm, vertical scale ×4 34
^{1}H NMR spectra (700.1 MHz): addition of CysHCl to MHB solution containing G1 in H_2O/D_2O (1:1) – expansion from 5.5 ppm to 0.5 ppm 35
^{1}H NMR spectra (700.1 MHz): addition of CysHCl to MHB solution containing G1 in H_2O/D_2O (1:1) – expansion from 4.4 ppm to 2.4 ppm 36
^{1}H NMR spectra (700.1 MHz): addition of CysHCl to MHB solution containing G1 in H_2O/D_2O (1:1) – expansion from 2.45 ppm to 0.6 ppm 37
Spectra of 2-bromo-5-(2-nitroethenyl)furan (3) solution in CD_3CN/H_2O 38
^{1}H NMR spectrum (700.1 MHz) of G1 and 2-bromo-5-(2-nitroethenyl)furan (3) mixture in CD_3CN/H_2O (1:1) 39
$^{13}C\{^{1}H\}$ and DEPT-135 NMR spectra (176.0 MHz) of G1 and 2-bromo-5-(2-nitroethenyl)furan (3) mixture in CD_3CN/H_2O (1:1) 40
$^{13}C\{^{1}H\}$ and DEPT-135 NMR spectra (176.0 MHz) of G1 and 2-bromo-5-(2-nitroethenyl)furan (3) mixture in CD_3CN/H_2O (1:1) – expansion from +115.0 ppm to +152.0 ppm 41
^{1}H-^{15}N HMBC NMR spectrum (700.1 MHz, 70.9 MHz) of G1 and 2-bromo-5-(2-nitroethenyl)furan (3) mixture in CD_3CN/H_2O (1:1) 42
Spectra of 2-bromo-5-(2-nitroethenyl)furan (3) solution in CD_3OD 43
^{1}H NMR spectrum (700.1 MHz) of 2-bromo-5-(2-nitroethenyl)furan (3) solution in CD_3OD 44
$^{13}C\{^{1}H\}$ and DEPT-135 NMR spectra (176.0 MHz) of 2-bromo-5-(2-nitroethenyl)furan (3) solution in CD_3OD 45
$^{13}C\{^{1}H\}$ and DEPT-135 NMR spectra (176.0 MHz) of 2-bromo-5-(2-nitroethenyl)furan (3) solution in CD_3OD – expansion from +112.0 ppm to +155.0 ppm 46
^{1}H-^{15}N HMBC NMR spectrum (700.1 MHz, 70.9 MHz) of 2-bromo-5-(2-nitroethenyl)furan (3) solution in CD_3OD 47
Spectra of 2-bromo-5-[2-nitro(2-^{2}H)ethenyl]furan solution in CD_3CN/D_2O 48
^{1}H NMR spectrum (700.1 MHz) of G1 and 2-bromo-5-[2-nitro(2-^{2}H)ethenyl]furan solution in CD_3CN/D_2O (1:1) 49
$^{13}C\{^{1}H\}$ and DEPT-135 NMR spectra (176.0 MHz) of G1 and 2-bromo-5-[2-nitro(2-^{2}H)ethenyl]furan solution in CD_3CN/D_2O (1:1) 50
$^{13}C\{^{1}H\}$ and DEPT-135 NMR spectra (176.0 MHz) of G1 and 2-bromo-5-[2-nitro(2-^{2}H)ethenyl]furan solution in CD_3CN/D_2O (1:1) – expansion from +115.0 ppm to +152.0 ppm 51
^{1}H-^{15}N HMBC NMR spectrum (700.1 MHz, 70.9 MHz) of G1 and 2-bromo-5-[2-nitro(2-^{2}H)ethenyl]furan solution in CD_3CN/D_2O (1:1) 52
Spectra of diastereoisomeric 1:1 mixture of *S*-[(1*R*)-1-(5-bromofuran-2-yl)-2-nitroethyl]-*L*-cysteine and *S*-[(1*S*)-1-(5-bromofuran-2-yl)-2-nitroethyl]-*L*-cysteine (4a and 4b) solution in CD_3CN/H_2O 53
HRMS of *S*-[(1*R*)-1-(5-bromofuran-2-yl)-2-nitroethyl]-*L*-cysteine and *S*-[(1*S*)-1-(5-bromofuran-2-yl)-2-nitroethyl]-*L*-cysteine (4) 55
^{1}H NMR spectrum (700.1 MHz) of *S*-[(1*R*)-1-(5-bromofuran-2-yl)-2-nitroethyl]-*L*-cysteine and *S*-[(1*S*)-1-(5-bromofuran-2-yl)-2-nitroethyl]-*L*-cysteine (4) mixture in CD_3CN/H_2O (1:1) 56
$^{13}C\{^{1}H\}$ and DEPT-135 NMR spectra (176.0 MHz) of *S*-[(1*R*)-1-(5-bromofuran-2-yl)-2-nitroethyl]-*L*-cysteine and *S*-[(1*S*)-1-(5-bromofuran-2-yl)-2-nitroethyl]-*L*-cysteine (4) mixture in CD_3CN/H_2O (1:1) 57
$^{13}C\{^{1}H\}$ and DEPT-135 NMR spectra (176.0 MHz) of *S*-[(1*R*)-1-(5-bromofuran-2-yl)-2-nitroethyl]-*L*-cysteine and *S*-[(1*S*)-1-(5-bromofuran-2-yl)-2-nitroethyl]-*L*-cysteine (4) mixture in CD_3CN/H_2O (1:1) – expansion from +110.0 ppm to +175.0 ppm 58
$^{13}C\{^{1}H\}$ and DEPT-135 NMR spectra (176.0 MHz) of *S*-[(1*R*)-1-(5-bromofuran-2-yl)-2-nitroethyl]-*L*-cysteine and *S*-[(1*S*)-1-(5-bromofuran-2-yl)-2-nitroethyl]-*L*-cysteine (4) mixture in CD_3CN/H_2O (1:1) – expansion from +22.0 ppm to +80.0 ppm 59
^{1}H-^{15}N HMBC NMR spectrum (700.1 MHz, 70.9 MHz) of *S*-[(1*R*)-1-(5-bromofuran-2-yl)-2-nitroethyl]-*L*-cysteine and *S*-[(1*S*)-1-(5-bromofuran-2-yl)-2-nitroethyl]-*L*-cysteine (4) mixture in CD_3CN/H_2O (1:1) 60
Spectra of diastereoisomeric 1:1 mixture of *S*-[(1*R*)-1-(5-bromofuran-2-yl)-2-nitro(2,2-$^{2}H_2$)ethyl]-*L*-(*N*,*N*,*O*-$^{2}H_3$)cysteine and *S*-[(1*S*)-1-(5-bromofuran-2-yl)-2-nitro(2,2-$^{2}H_2$)ethyl]-*L*-(*N*,*N*,*O*-$^{2}H_3$)cysteine solution in CD_3CN/D_2O 61
^{1}H NMR spectrum (700.1 MHz) of *S*-[(1*R*)-1-(5-bromofuran-2-yl)-2-nitro(2,2-$^{2}H_2$)ethyl]-*L*-(*N*,*N*,*O*-$^{2}H_3$)cysteine and *S*-[(1*S*)-1-(5-bromofuran-2-yl)-2-nitro(2,2-$^{2}H_2$)ethyl]-*L*-(*N*,*N*,*O*-$^{2}H_3$)cysteine solution in CD_3CN/D_2O (1:1) 63

$^{13}C\{^{1}H\}$ and DEPT-135 NMR spectra (176.0 MHz) of *S*-[(1*R*)-1-(5-bromofuran-2-yl)-2-nitro(2,2-$^{2}H_2$)ethyl]-*L*-(*N*,*N*,*O*-$^{2}H_3$)cysteine and *S*-[(1*S*)-1-(5-bromofuran-2-yl)-2-nitro(2,2-$^{2}H_2$)ethyl]-*L*-(*N*,*N*,*O*-$^{2}H_3$)cysteine solution in CD_3CN/D_2O (1:1) 64
$^{13}C\{^{1}H\}$ and DEPT-135 NMR spectra (176.0 MHz) of *S*-[(1*R*)-1-(5-bromofuran-2-yl)-2-nitro(2,2-$^{2}H_2$)ethyl]-*L*-(*N*,*N*,*O*-$^{2}H_3$)cysteine and *S*-[(1*S*)-1-(5-bromofuran-2-yl)-2-nitro(2,2-$^{2}H_2$)ethyl]-*L*-(*N*,*N*,*O*-$^{2}H_3$)cysteine solution in CD_3CN/D_2O (1:1) – expansion from +110.0 ppm to +175.0 ppm 65
$^{13}C\{^{1}H\}$ and DEPT-135 NMR spectra (176.0 MHz) of *S*-[(1*R*)-1-(5-bromofuran-2-yl)-2-nitro(2,2-$^{2}H_2$)ethyl]-*L*-(*N*,*N*,*O*-$^{2}H_3$)cysteine and *S*-[(1*S*)-1-(5-bromofuran-2-yl)-2-nitro(2,2-$^{2}H_2$)ethyl]-*L*-(*N*,*N*,*O*-$^{2}H_3$)cysteine solution in CD_3CN/D_2O (1:1) – expansion from +22.0 ppm to +80.0 ppm 66

Spectra of 2-bromo-5-[1-(decylsulfanyl)-2-nitroethyl]furan (5) solution in $CDCl_3$ 67
^{1}H NMR spectrum (700.1 MHz) of 2-bromo-5-[1-(decylsulfanyl)-2-nitroethyl]furan (5) solution in $CDCl_3$ ... 68
$^{13}C\{^{1}H\}$ and DEPT-135 NMR spectra (176.0 MHz) of 2-bromo-5-[1-(decylsulfanyl)-2-nitroethyl]furan (5) solution in $CDCl_3$ 69
$^{13}C\{^{1}H\}$ and DEPT-135 NMR spectra (176.0 MHz) of 2-bromo-5-[1-(decylsulfanyl)-2-nitroethyl]furan (5) solution in $CDCl_3$ – expansion from +105.0 ppm to +155.0 ppm 70
$^{13}C\{^{1}H\}$ and DEPT-135 NMR spectra (176.0 MHz) of 2-bromo-5-[1-(decylsulfanyl)-2-nitroethyl]furan (5) solution in $CDCl_3$ – expansion from +12.0 ppm to +81.0 ppm 71
^{1}H-^{15}N HMBC NMR spectrum (700.1 MHz, 70.9 MHz) of 2-bromo-5-[1-(decylsulfanyl)-2-nitroethyl]furan (5) solution in $CDCl_3$ 72

Figure S1. Conversion of G1 in cell culture medium and bacterial complete medium 73

General details of the experimental part

All reagents were purchased from commercial sources (Sigma-Aldrich, Lach-Ner) and used without further purification.

Column chromatography was performed on silica gel (Merck Kieselgel 70-230 mesh). TLC analyses were carried out on TLC plates from Macherey-Nagel (Alugram SIL G/UV254) and visualized via UV-light (254 nm) or in standard coloring solution of 1% phosphomolybdic acid in EtOH. Preparative HPLC purification was carried out using a Shimadzu Prominence LC-MS chromatograph and a Phenomenex Luna 5µ C_{18}-column (250×10 mm) with a C_{18} Phenomenex SecurityGuard pre-column cartridge (10×10 mm), with the mobile phase of 10-100% gradient H_2O (MilliQ grade) methanol (LC-MS grade, Scharlau) mixture containing 10 mM ammonium acetate (analytical grade, BioTop), flow rate 2.0 mL/min, column oven at 35 °C.

For compound characterization, the ^{1}H, ^{13}C and ^{15}N NMR spectra were recorded at 700.1 (^{1}H), 176.0 (^{13}C) and 70.9 (^{15}N) MHz on a Bruker AVANCE III 700 NMR spectrometer. The ^{15}N NMR chemical shifts were indirectly obtained from ^{1}H detected ^{1}H-^{15}N gs-HMBC or ^{1}H-^{15}N gs-HSQC spectra. All NMR measurements were carried out at +20 °C. For ^{1}H NMR spectra, water-suppression pulse sequences using excitation sculpting or presaturation were used. Chemical shifts (^{1}H and ^{13}C) were indirectly referenced to TMS *via* the residual solvent signal ($CDCl_3$: 7.26 and 77.0 ppm, respectively; CD_3CN: 1.94 and 1.32 ppm, respectively; CD_3OD: 3.31 and 49.0 ppm, respectively). The ^{15}N chemical shifts were referenced externally to the signal of neat nitromethane (381.7 ppm). NMR signals were assigned from gs-HSQC, gs-HMBC, gs-NOESY and TOCSY spectra. The reported ^{13}C NMR data corresponds to the ^{1}H-decoupled ^{13}C NMR data (noted on spectra as "$^{13}C\{^{1}H\}$"), where the ^{1}H-^{13}C couplings have been removed. Abbreviations used to define NMR spectral multiplicities are as follows: d = doublet; m = multiplet; bs = broad signal; vt = triplet with virtual couplings.

HRMS measurements were performed on a Thermo Electron LTQ Orbitrap mass spectrometer.

The mammalian cytotoxicity was assayed using the xCELLigence RTCA DP Instrument (Roche).

OD_{390} were recorded with 1 s intervals using spectrophotometer Ultrospec 7000 (GE Healthcare Life Sciences).

Synthetic procedure

2-Bromo-5-(2-bromo-2-nitroethenyl)furan (Furvina or G1)

We have chemically synthesized G1 according to the synthetic scheme of Scholz and colleagues (Scholz *et al*, 2013) with minor modifications.

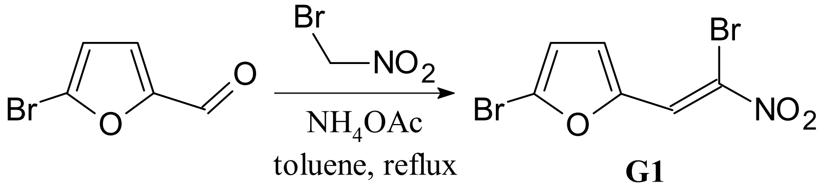

The starting material, 5-bromo-2-furaldehyde (6.00 g, 34.29 mmol), was dissolved in toluene (60 mL). Bromonitromethane (2.40 mL, 34.30 mmol) and ammonium acetate (420 mg, 5.45 mmol) were added, and the reaction mixture was refluxed using Dean-Stark apparatus for 4 hours. After that, more ammonium acetate (0.12 g, 1.56 mmol) and bromonitromethane (1.00 mL, 14.34 mmol) were added. Reaction mixture refluxed for additional 18 hours, cooled to room temperature and the solvent was evaporated. The desired compound was purified by column chromatography on silica in PE:EtOAc (20:1), resulting in 1.20 g (4.04 mmol, 12% yield) of **G1** in a form of orange solid. The chemical purity of the compound was confirmed by TLC and NMR.

Identification of the conversion products of G1

HPLC-HRMS analysis of reactivity of G1 towards cysteine

In order to study the reactivity of **G1** towards cysteine at pH 6.0, 10 µL **G1** stock solution (10 mM in DMSO) and 10 µL cysteine hydrochloride stock solution (10 mM in H_2O) were dissolved in 980 µL sodium citrate buffer (50 mM, pH 6.0). Aliquots (100 µL) of the reaction mixture were withdrawn every 60 s over a period of 7 min and the reaction in these aliquots was then stopped by quenching with 1 µL of orthophosphoric acid (85%). Samples were analyzed by HPLC (Figure 1).

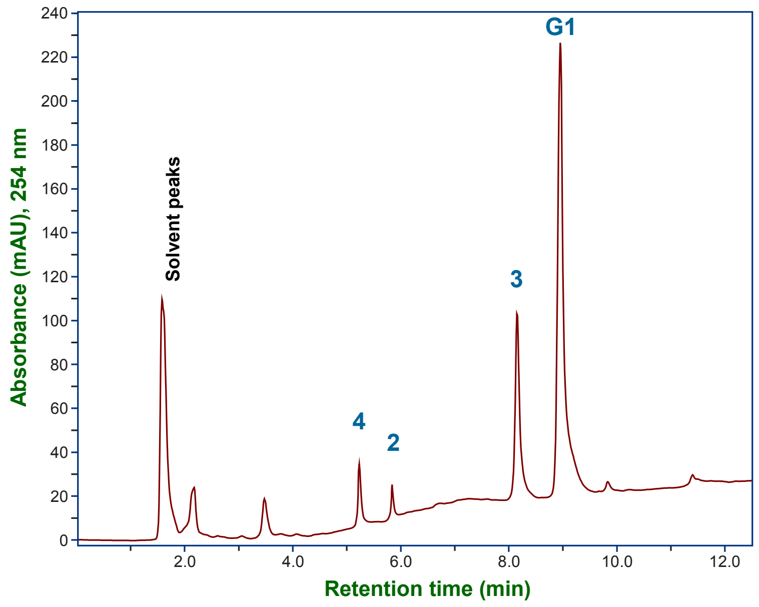

Analytical HPLC (C_{18} column, 150×4.6 mm, particle size 3 µm; mobile phase: 10% to 95% gradient over 9 minutes of 0.1% formic acid in H_2O and 0.1% formic acid in acetonitrile; flow-rate 1.0 mL/min) chromatogram after 4 minutes incubation with cysteine.

The fractions corresponding to te peaks **2** and **4** were collected separately and the contents were analyzed by HRMS (FTMS+pNSI).

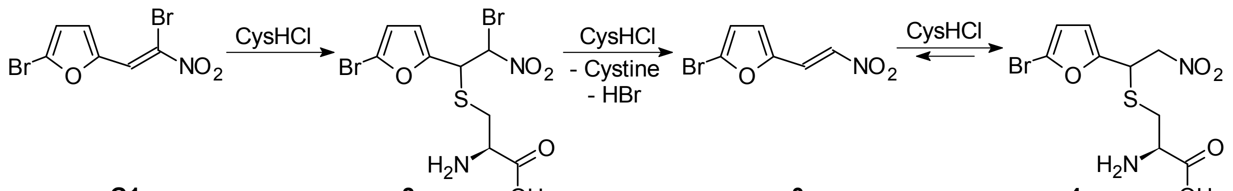

G1 **2** **3** **4**

Structures of conversion products of **G1** at pH 6.0 as detected using HPLC-HRMS analysis.

G1: R_t=8.94 min, λ_{max}=383 nm.

2: R_t=5.84 min, λ_{max}=216 nm, HRMS (m/z) calcd for $C_9H_{10}Br_2N_2O_5S+H^+$ $(M+H)^+$ 416.8750, found 416.8742.

3: R_t=8.14 min, λ_{max}=359 nm.

4: R_t=5.22 min, λ_{max}=212 nm, HRMS (m/z) calcd for $C_9H_{11}BrN_2O_5S+H^+$ $(M+H)^+$ 338.9645, found 338.9634.

Ultraviolet-visible spectra of compounds G1, 2, 3 and 4

The UV-vis spectra are the photo diode array detector readouts between 190 and 800 nm at the corresponding peaks' retention times in the mobile phase (0.1% formic acid in H_2O and acetonitrile mixture).

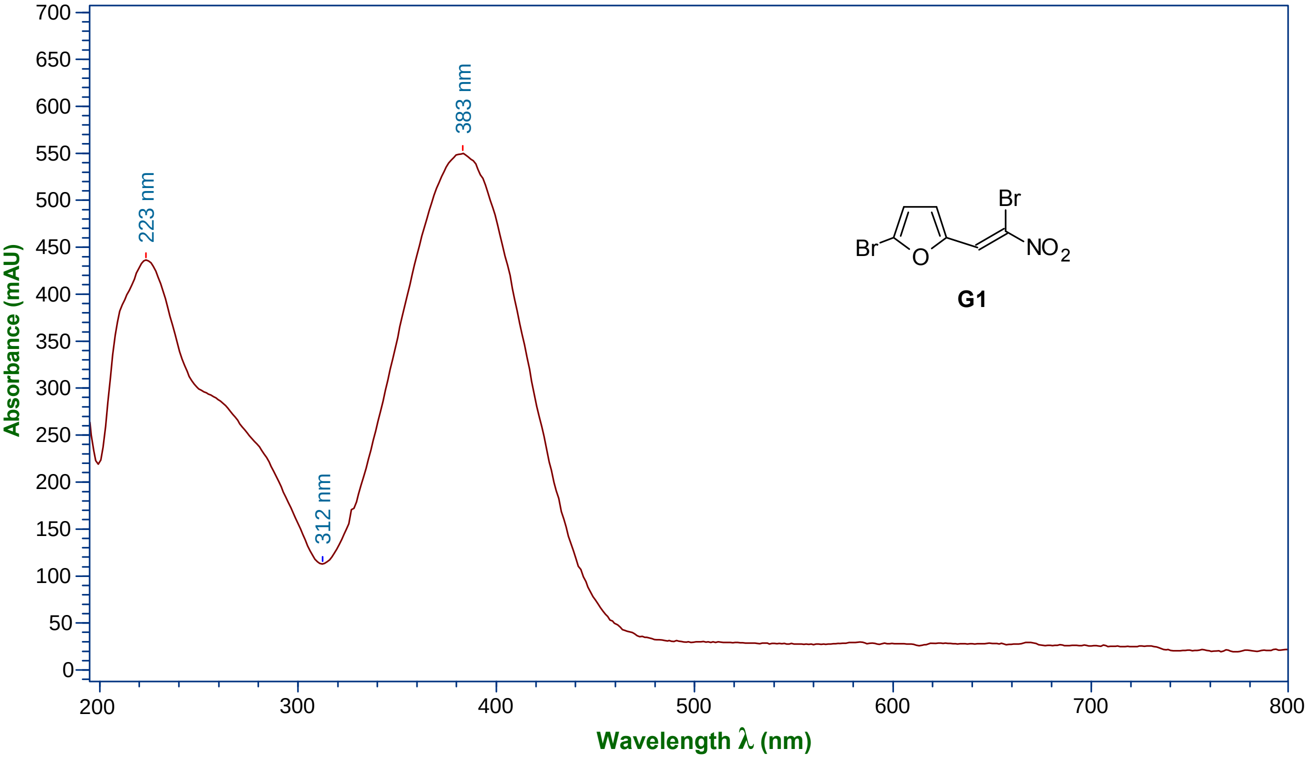

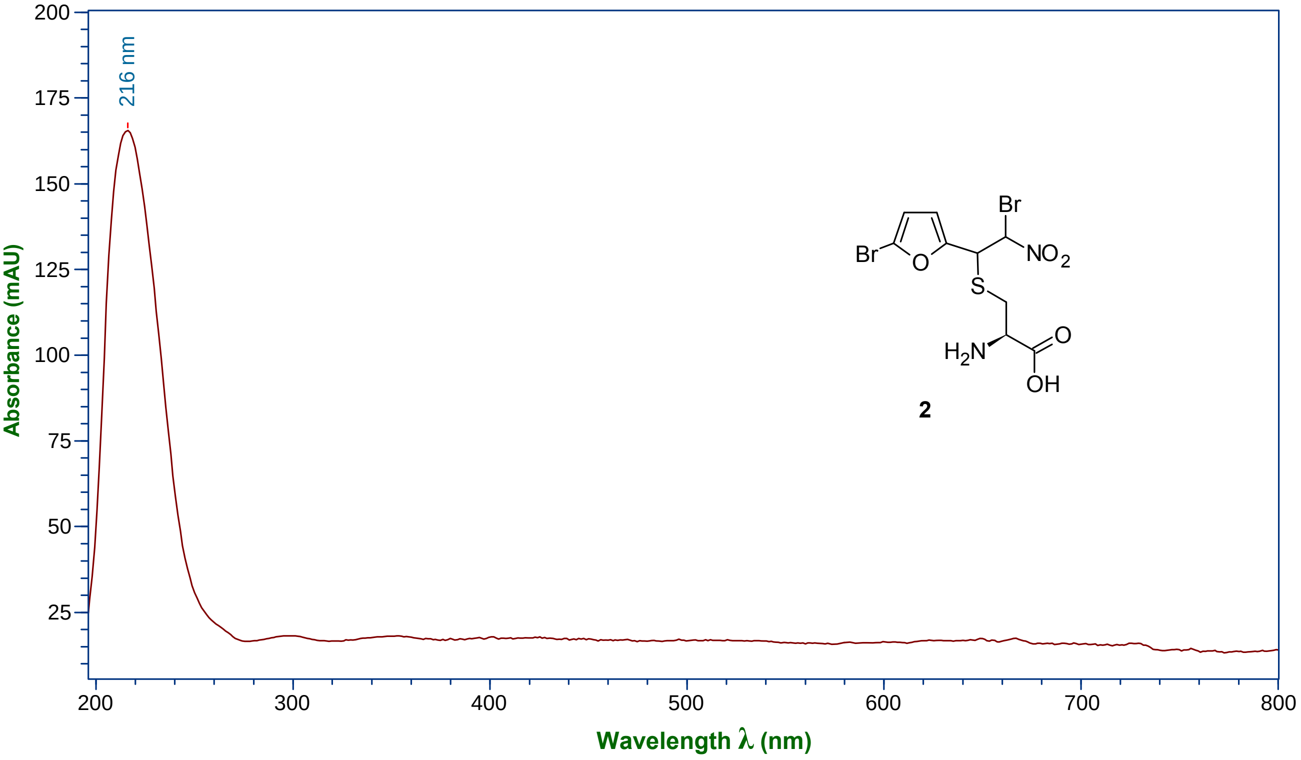

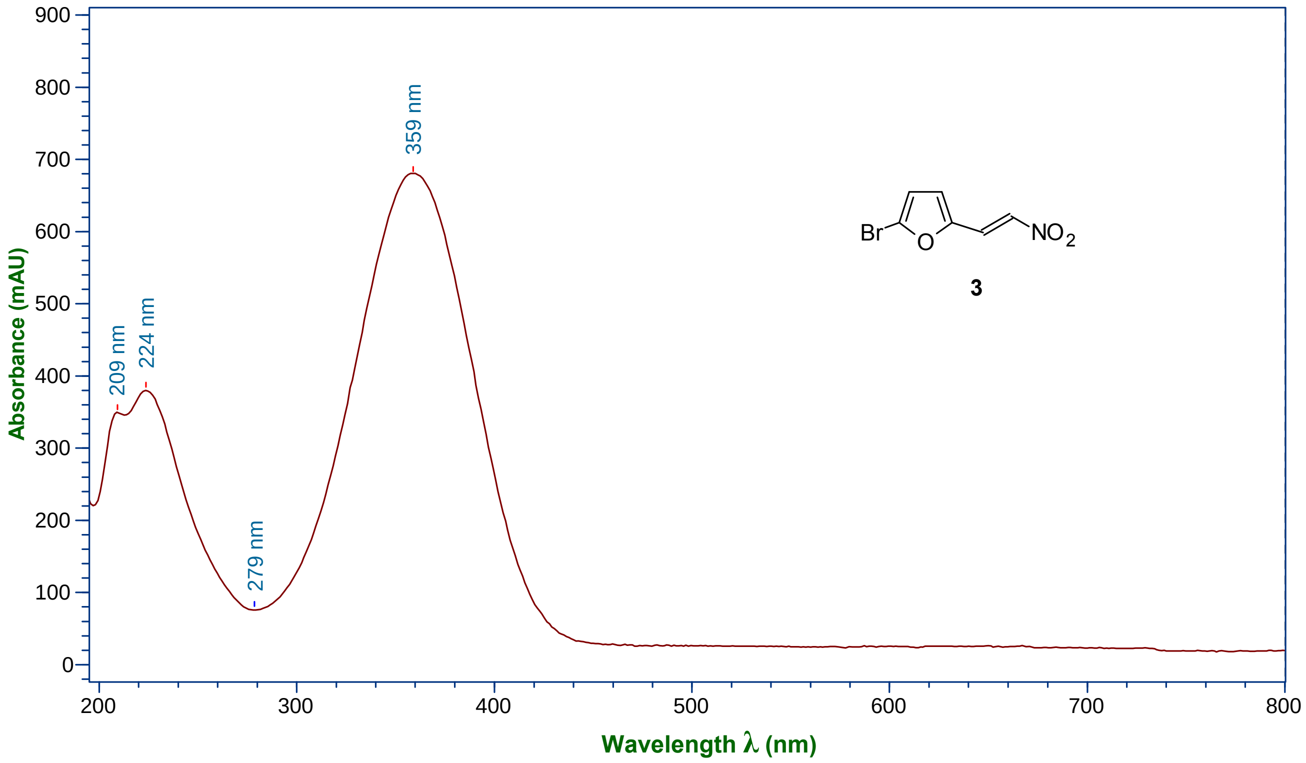

Absorbance (mAU)
Wavelength λ (nm)
209 nm
224 nm
279 nm
359 nm
Br
O
NO2
3

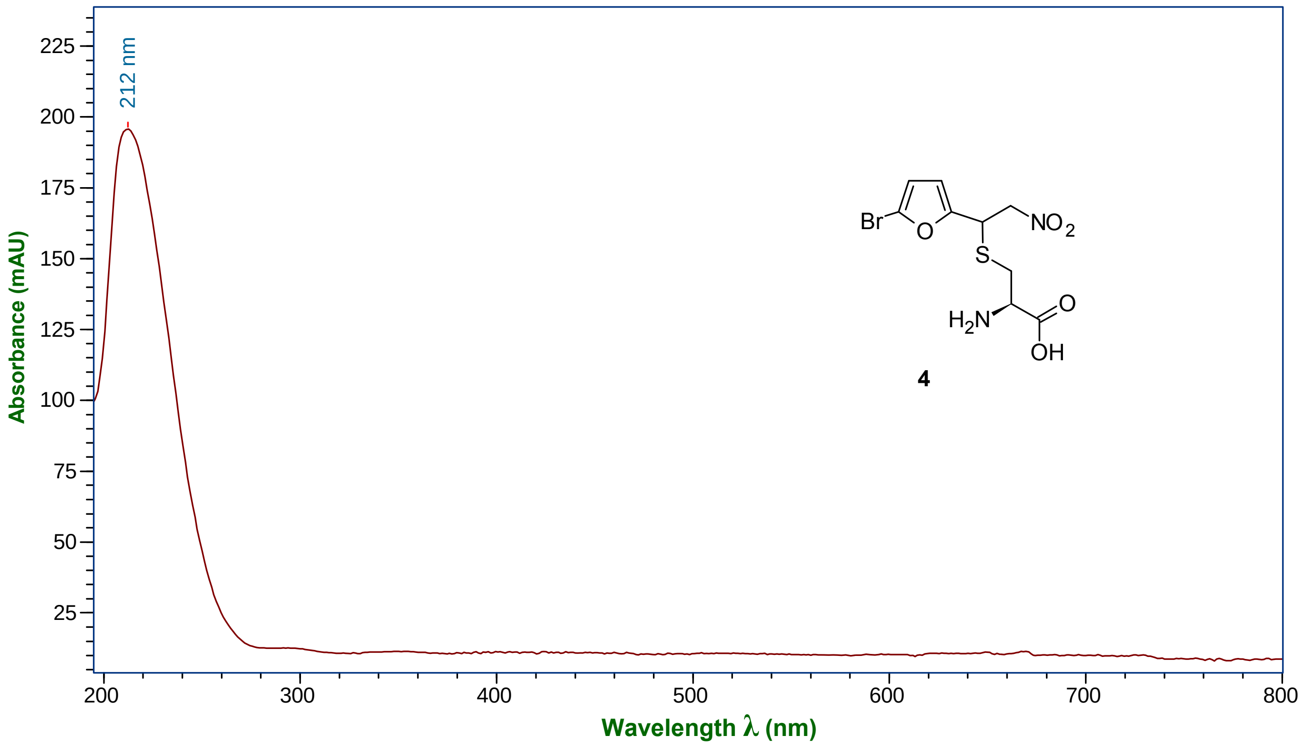

Absorbance (mAU)
Wavelength λ (nm)
212 nm
Br
O
NO2
S
H2N
O
OH
4

HRMS spectrum of 2

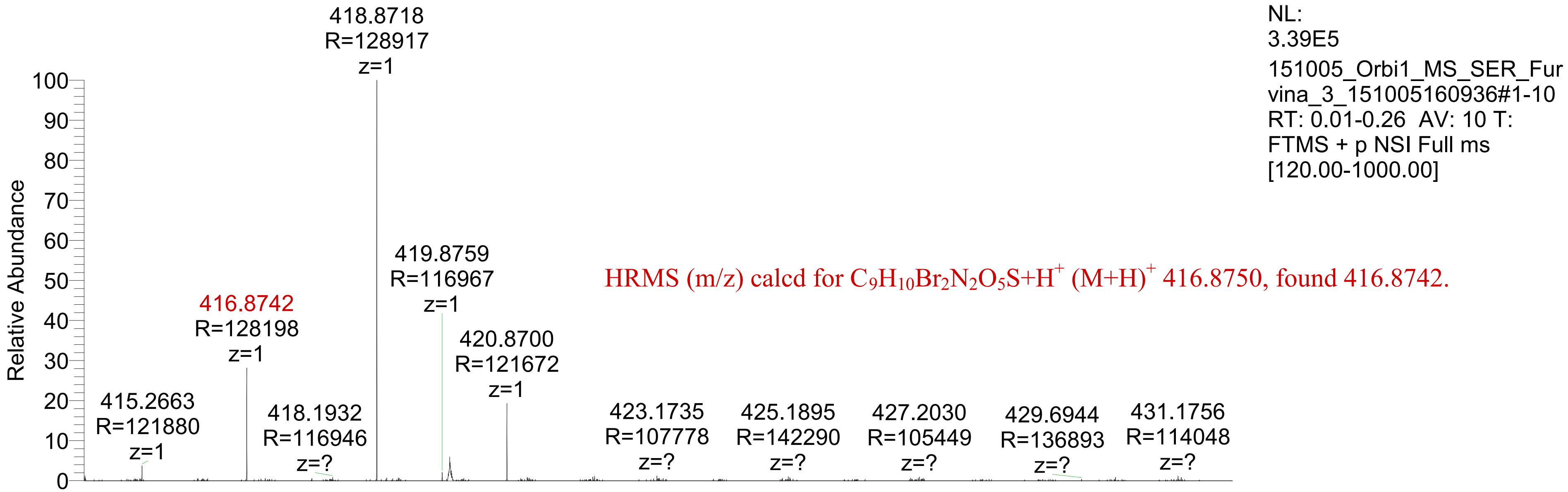

HRMS (m/z) calcd for $C_9H_{10}Br_2N_2O_5S+H^+$ $(M+H)^+$ 416.8750, found 416.8742.

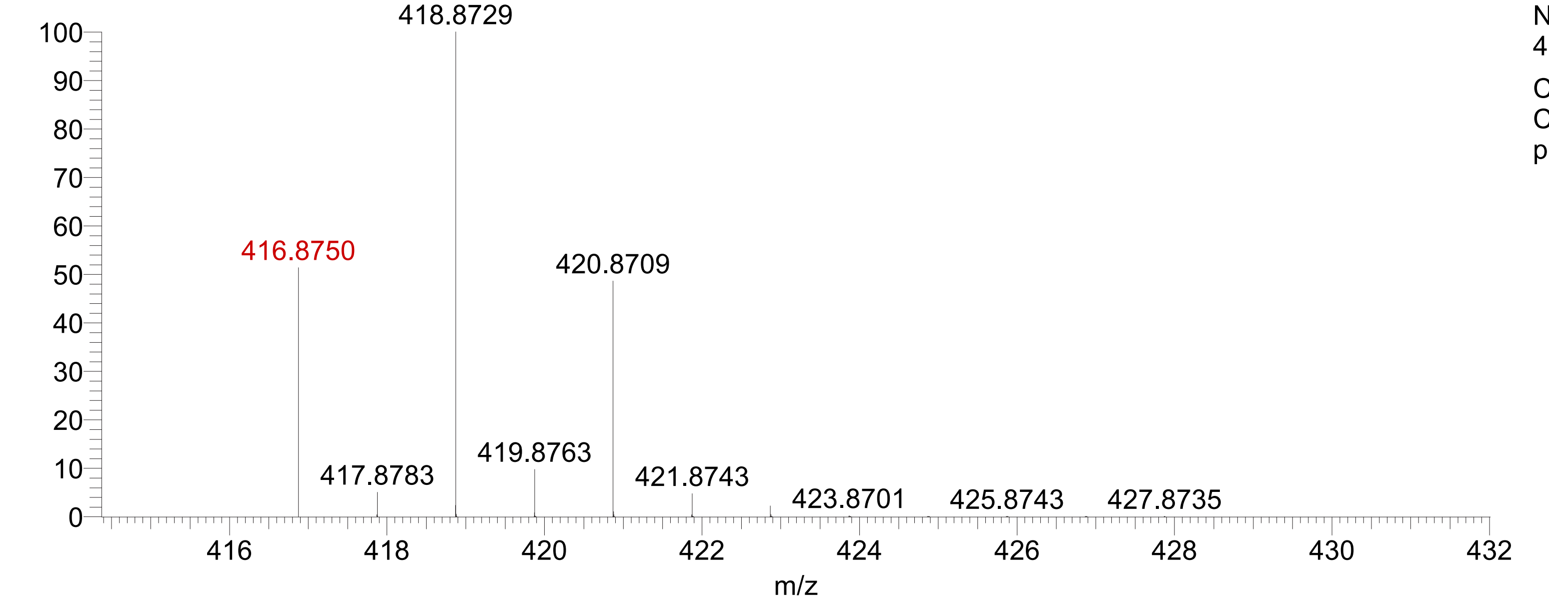

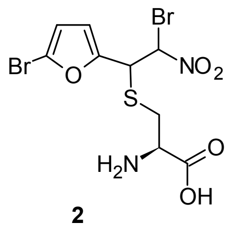

2

HRMS spectrum of 4

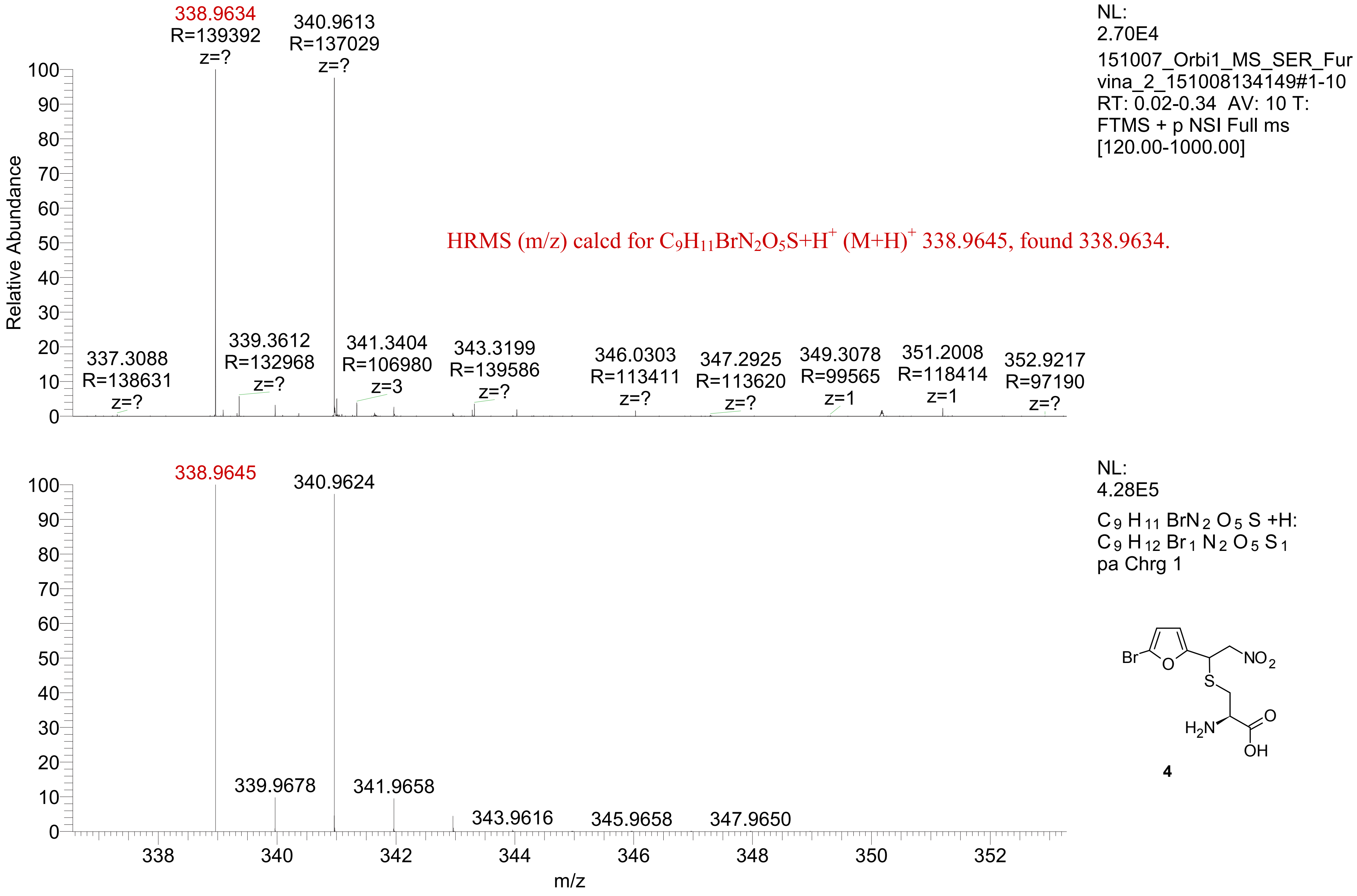

HRMS (m/z) calcd for $C_9H_{11}BrN_2O_5S+H^+$ $(M+H)^+$ 338.9645, found 338.9634.

2-Bromo-5-(2-nitroethenyl)furan (3)

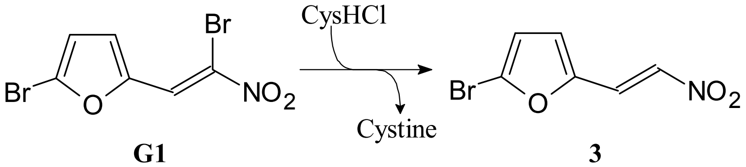

G1 **3**

Case I:

G1 (3.4 mg, 11.5 μmol) was dissolved in CD_3CN (0.3 mL), followed by addition of H_2O (0.3 mL). Saturated solution of $NaHCO_3$ in H_2O (13 μL) was added via a syringe, followed by addition of cysteine hydrochloride (1.6 mg, 10.2 μmol) solution in H_2O (22 μL). The formation of the compound **2** was followed by measuring ^1H NMR spectra. Using this amount of CysHCl gave approximately 0.7:1.0 mixture of unreacted G1 and the product **3**.

After separation of the insoluble cystine by filtration using a plug of cotton wool, an analytical sample of **3** was obtained by purification with preparative HPLC (R_t=21.5-23.5 min, λ_{max}=350 nm).

Case II:

In a cation-adjusted Mueller-Hinton II broth (MHB, 20 mg) aqueous (0.4 mL H_2O and 0.4 mL D_2O) medium, **G1** (0.40 mg, 1.3 μmol) solution in DMSO-d_6 (40 μL) was added, followed by addition of cysteine hydrochloride (0.46 mg, 2.9 μmol) solution in H_2O (20 μL). Appearance of products **3** and **4** signals were observed by ^1H NMR spectroscopy. The appeared ^1H NMR signals observed in the MHB medium were the same as were present in the reaction mixture, where inorganic base and organic co-solvent were used.

2-Bromo-5-[2-nitro(2-^{2}H)ethenyl]furan

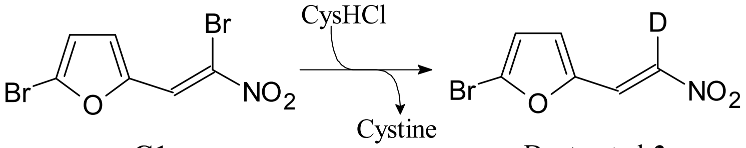

G1 Deuterated-**3**

G1 (3.4 mg, 11.5 μmol) was dissolved in CD_3CN (0.3 mL), followed by addition of D_2O (0.3 mL). Saturated solution of $NaHCO_3$ in D_2O (10 μL) was added via a syringe, followed by addition of cysteine hydrochloride (1.5 mg, 9.5 μmol) solution in D_2O (20 μL). The formation of

the monodeuterated product **3** was followed by measuring ^1H NMR spectra. Using this amount of CysHCl gave approximately 1:1 mixture of unreacted **G1** and the monodeuterated product **3**.

Diastereoisomeric 1:1 mixture of *S*-[(1*R*)-1-(5-bromofuran-2-yl)-2-nitroethyl]-*L*-cysteine and *S*-[(1*S*)-1-(5-bromofuran-2-yl)-2-nitroethyl]-*L*-cysteine (4a and 4b)

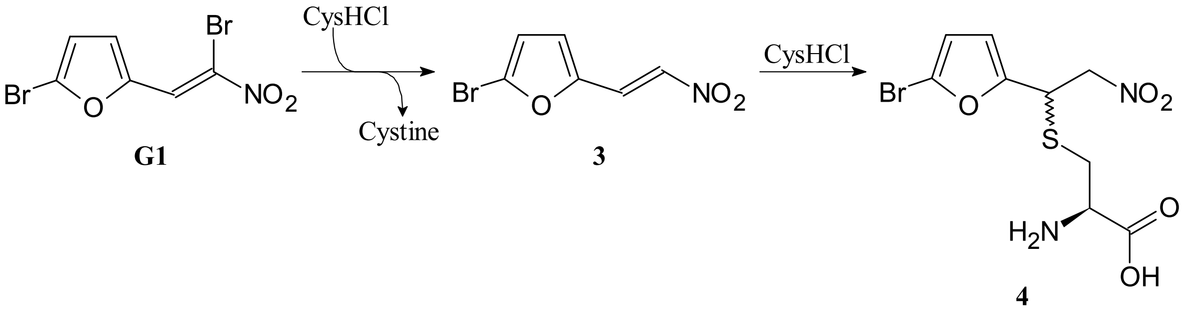

G1 **3** **4**

Case I:

To the same reaction mixture in CD_3CN/H_2O (1:1), where compound **3** was obtained and determined, excess of cysteine hydrochloride solution in H_2O was added until there were no signals in the ^1H NMR spectrum corresponding to **G1** or compound **3**. Diastereoisomeric 1:1 mixture of the obtained product **4** was determined by NMR.

Case II:

In a cation-adjusted Mueller-Hinton II broth (MHB, 20 mg) aqueous (0.5 mL H_2O and 0.1 mL D_2O) medium, cysteine hydrochloride (1.8 mg, 11.4 μmol) solution in H_2O (100 μL) was added, followed by addition of G1 (15 μg, 0.050 μmol) solution in DMSO-d_6 (10 μL). Appearance of product **4** signals were observed by ^1H NMR spectroscopy, where the characteristic signals at 6.31 and 6.40 ppm, corresponding to the product's furane-ring hydrogens, were clearly visible and well-separated from all other signals in the ^1H NMR spectrum.

Case III:

In a cation-adjusted Mueller-Hinton II broth (MHB, 20 mg) aqueous (0.4 mL H_2O and 0.4 mL D_2O) medium, G1 (0.40 mg, 1.3 μmol) solution in DMSO-d_6 (40 μL) was added, followed by addition of cysteine hydrochloride (1.4 mg, 8.9 μmol) solution in H_2O (60 μL). Although an excess of CysHCl was used, this time a mixture of unreacted **G1** and product **4** was obtained, indicating that in an unbuffered solution and with higher **G1** loading, the product **4** formation can be slow.

Diastereoisomeric 1:1 mixture of *S*-[(1*R*)-1-(5-bromofuran-2-yl)-2-nitro(2,2-$^{2}H_2$)ethyl]-*L*-(*N*,*N*,*O*-$^{2}H_3$)cysteine and *S*-[(1*S*)-1-(5-bromofuran-2-yl)-2-nitro(2,2-$^{2}H_2$)ethyl]-*L*-(*N*,*N*,*O*-$^{2}H_3$)cysteine

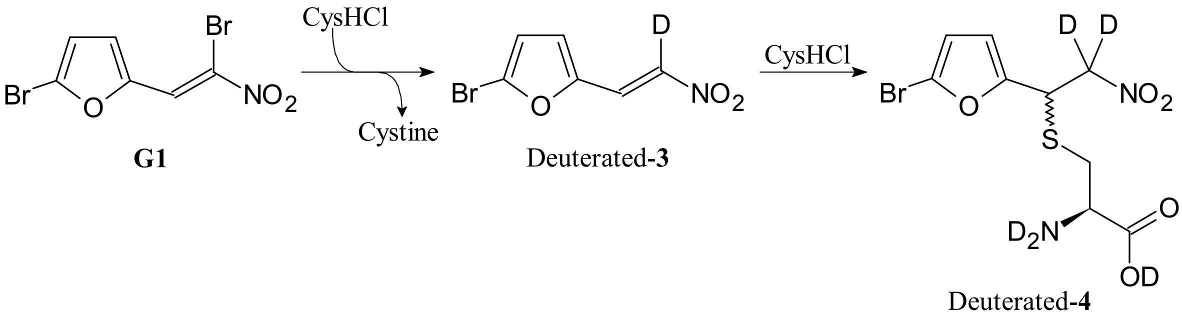

G1 (3.4 mg, 11.5 µmol) was dissolved in CD_3CN (0.3 mL), followed by addition of D_2O (0.3 mL). Saturated solution of $NaHCO_3$ in D_2O (15 µL) was added via a syringe, followed by addition of cysteine hydrochloride (7.3 mg, 46.3 µmol) solution in D_2O (105 µL). The formed cystine precipitate was filtered off using a plug of cotton wool. The filtrate composition was analyzed by NMR, which showed the presence of 1:1 diastereomeric mixture of the deuterated product **4** and cysteine.

2-Bromo-5-[1-(decylsulfanyl)-2-nitroethyl]furan (5)

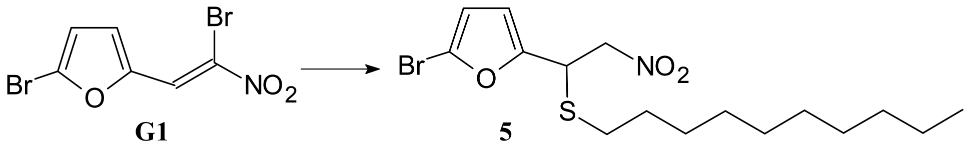

G1 (6.5 mg, 21.9 µmol) was dissolved in CH_3OH (0.3 mL), followed by addition of 1-decanethiol (18 mg, 0.10 mmol) and saturated aqueous solution of $NaHCO_3$ (80 µL). After mixing, the solvent was evaporated under vacuum and the product was dissolved in $CDCl_3$ for NMR studies.

NMR spectra and analytical data

Analytical data and copies of NMR and HRMS spectra.

Spectra of 2-bromo-5-(2-bromo-2-nitroethenyl)furan (G1) solution in $CDCl_3$

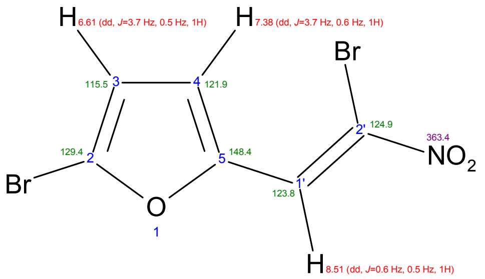

^{1}H NMR (700.1 MHz, $CDCl_3$, 20.0 °C) δ: 8.51 (dd, $^4J_{HH}$ = 0.6 Hz, $^5J_{HH}$ = 0.5 Hz, 1H, C*H*-1'); 7.38 (dd, $^3J_{HH}$ = 3.7 Hz, $^4J_{HH}$ = 0.6 Hz, 1H, C*H*-4); 6.61 (dd, $^3J_{HH}$ = 3.7 Hz, $^5J_{HH}$ = 0.5 Hz, C*H*-3). ^{13}C{^{1}H} NMR (176.0 MHz, $CDCl_3$, 20.0 °C) δ: 148.4 (*C*-5); 129.4 (*C*-2); 124.9 (*C*-2'); 123.8 (*C*H-1'); 121.9 (*C*H-4); 115.5 (*C*H-3). ^{15}N NMR (70.9 MHz, $CDCl_3$, 20.0 °C) δ: 363.4 (*N*O_2).

^{1}H NMR spectrum (700.1 MHz) of G1 in $CDCl_3$

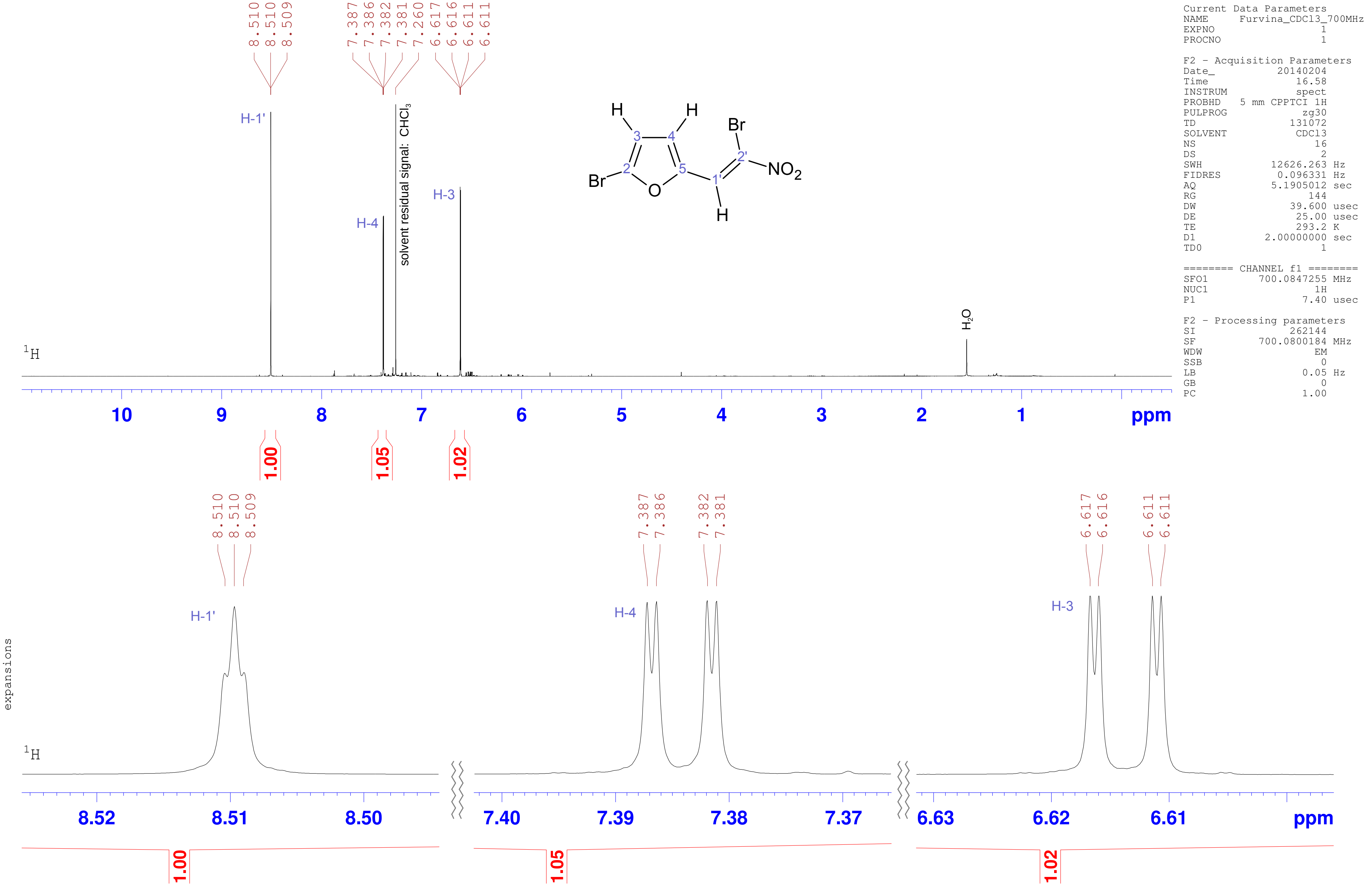

$^{13}C\{^1H\}$ and DEPT-135 NMR spectra (176.0 MHz) of G1 in $CDCl_3$

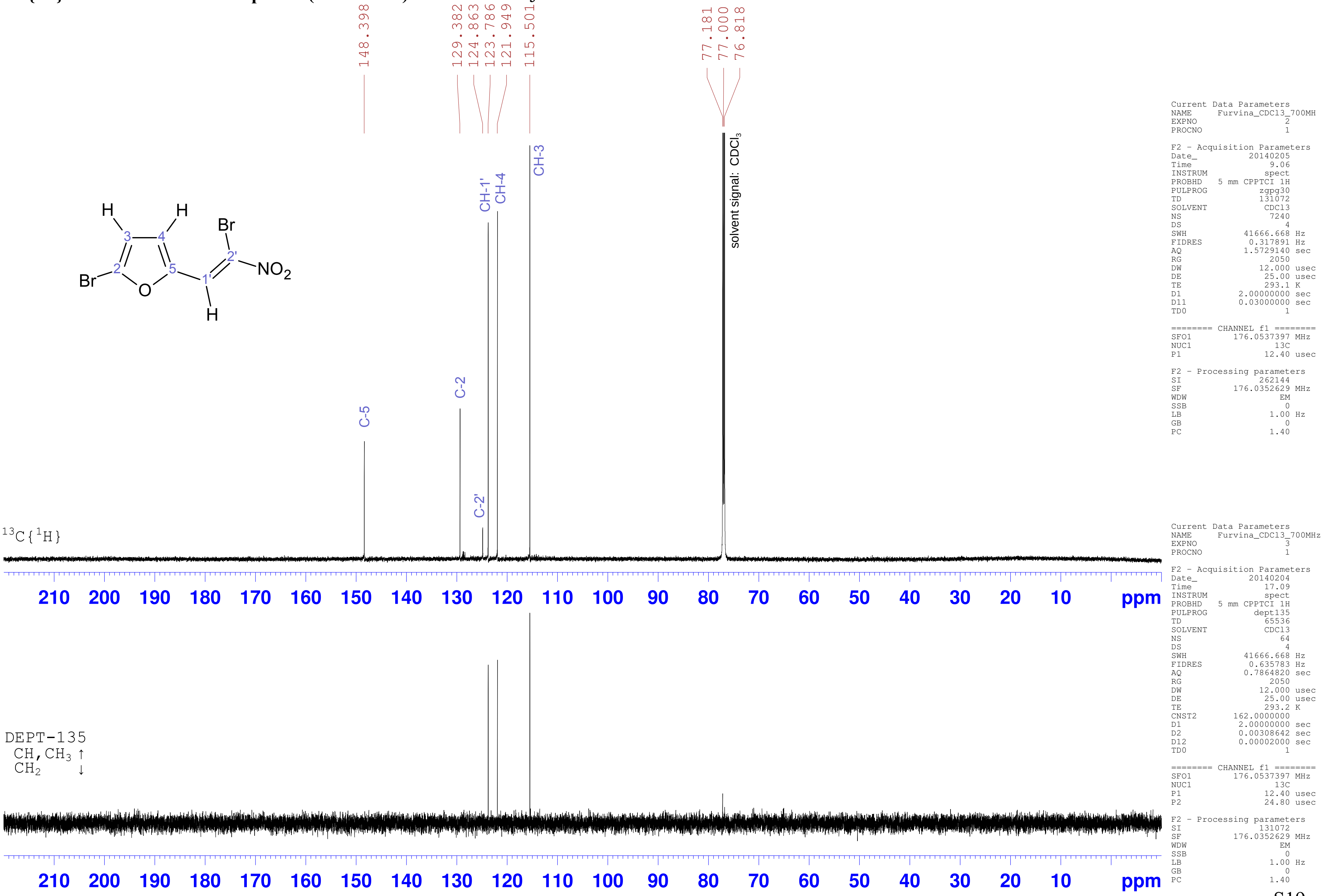

$^{13}C\{^1H\}$ and DEPT-135 NMR spectra (176.0 MHz) of G1 in $CDCl_3$ – expansion from +113.0 ppm to +152.0 ppm

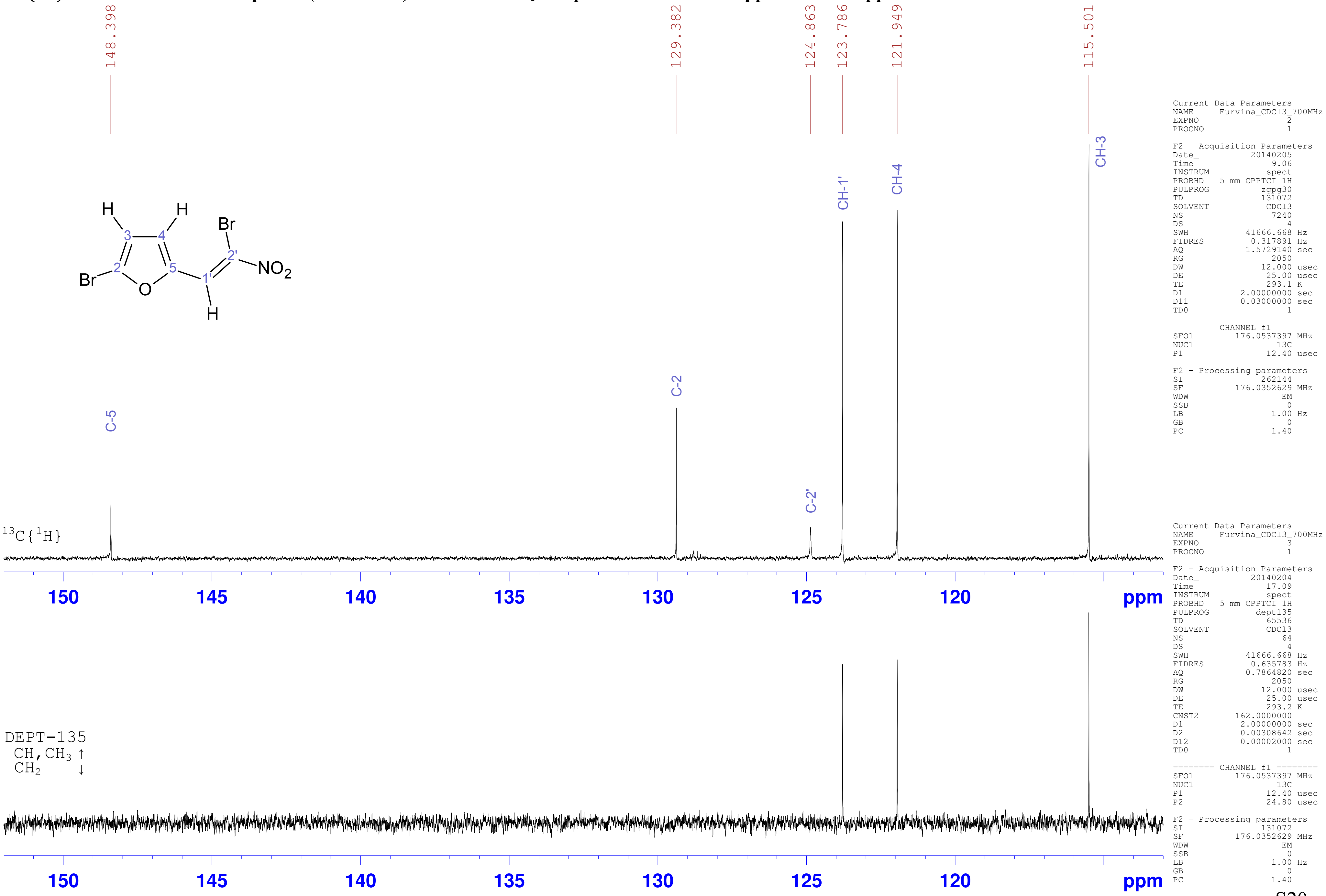

^{1}H-^{15}N HMBC NMR spectrum (700.1 MHz, 70.9 MHz) of G1 in $CDCl_3$

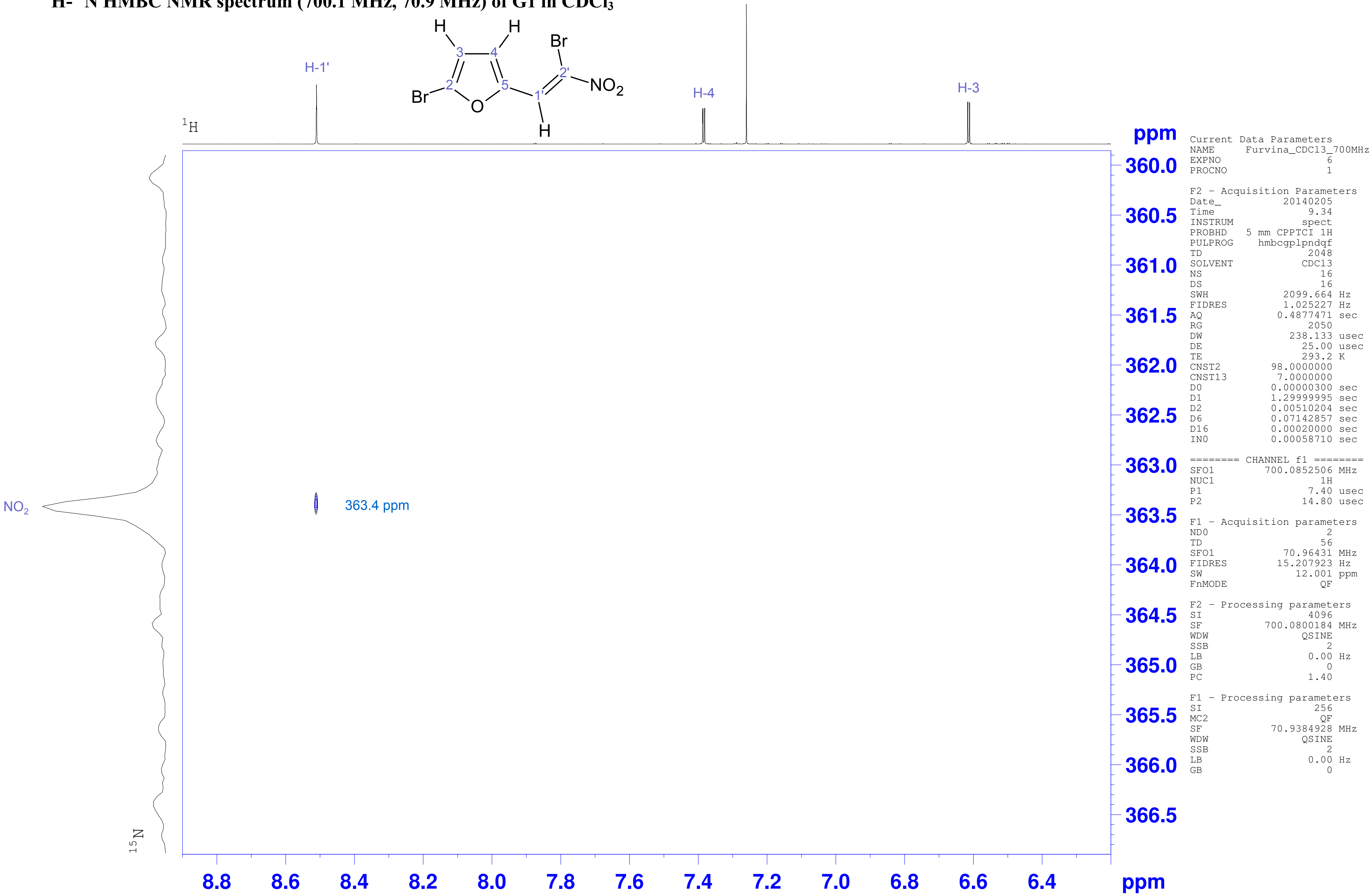

Stacked ^{1}H NMR spectra: titration of G1 with CysHCl in the presence of $NaHCO_3$ in CD_3CN/H_2O (1:1)

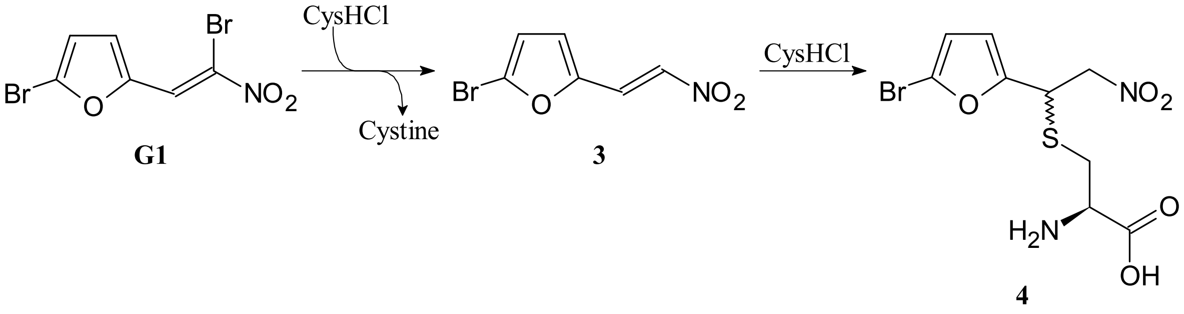

G1 (3.4 mg, 11.5 μmol) was dissolved in CD_3CN (0.3 mL), followed by addition of H_2O (0.3 mL). Saturated solution of $NaHCO_3$ in H_2O (13 μL) was added via a syringe, followed by addition of cysteine hydrochloride (1.6 mg, 10.2 μmol) solution in H_2O (22 μL).

Then, excess of cysteine hydrochloride solution in H_2O was added until there were no signals in the ^{1}H NMR spectrum corresponding to **G1** or compound **3**.

^{1}H NMR spectra (700.1 MHz): titration of G1 with CysHCl in the presence of $NaHCO_3$ in CD_3CN/H_2O (1:1) – expansion from -0.5 ppm to +11 ppm

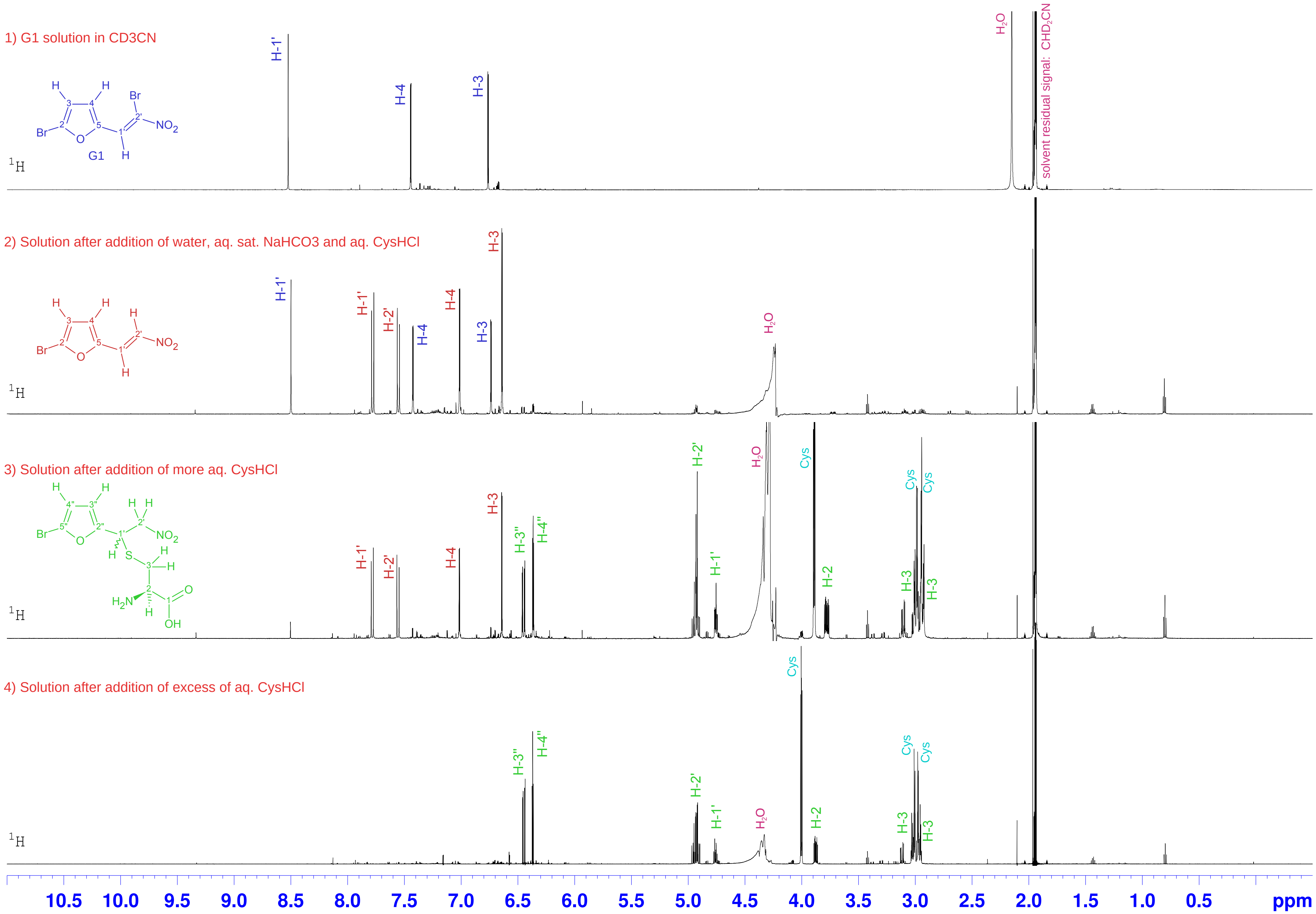

^{1}H NMR spectra (700.1 MHz): titration of G1 with CysHCl in the presence of $NaHCO_3$ in CD_3CN/H_2O (1:1) – expansion from +6.2 ppm to +8.7 ppm

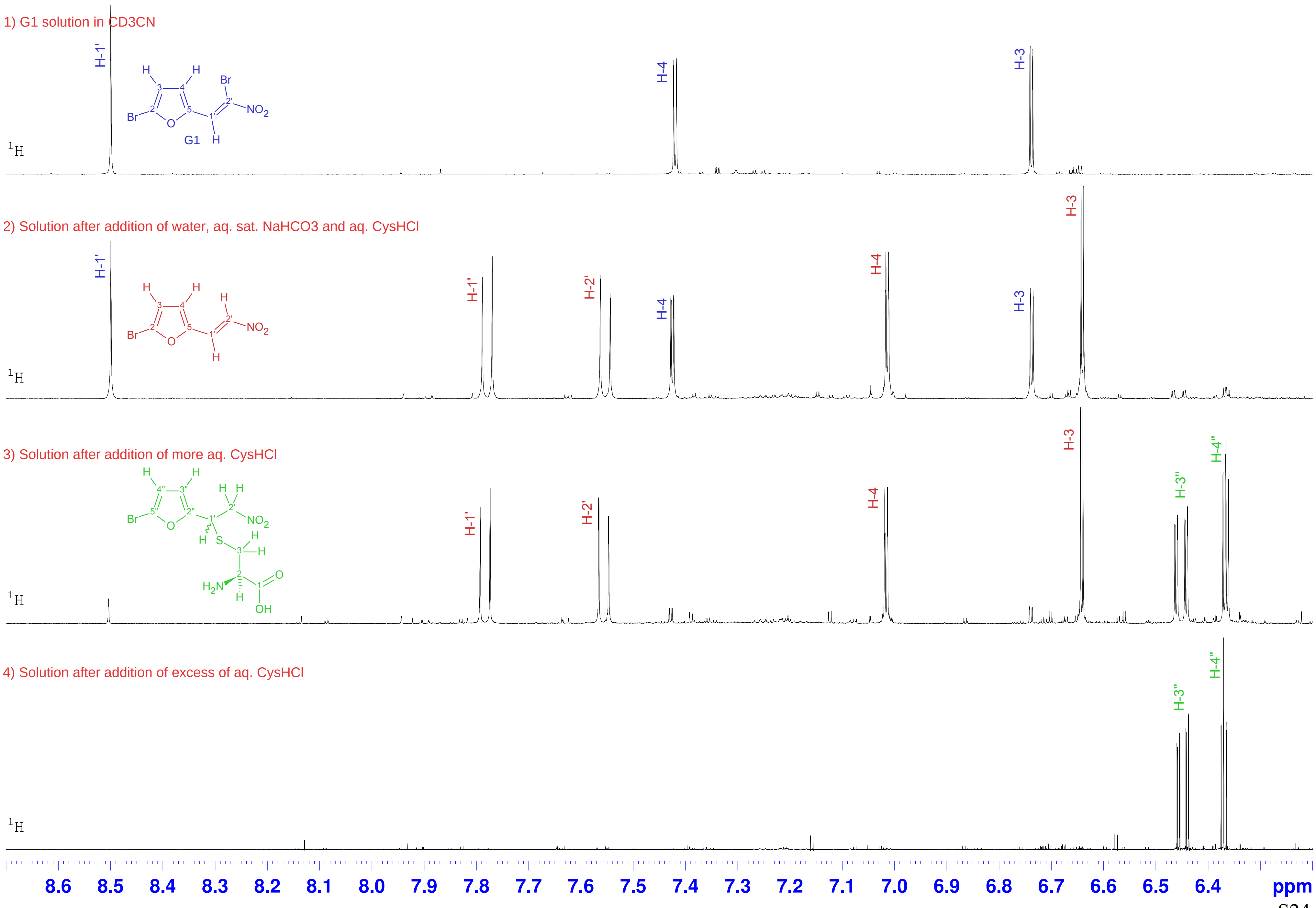

^{1}H NMR spectra (700.1 MHz): titration of G1 with CysHCl in the presence of $NaHCO_3$ in CD_3CN/H_2O (1:1) – expansion from +2.8 ppm to +5.1 ppm

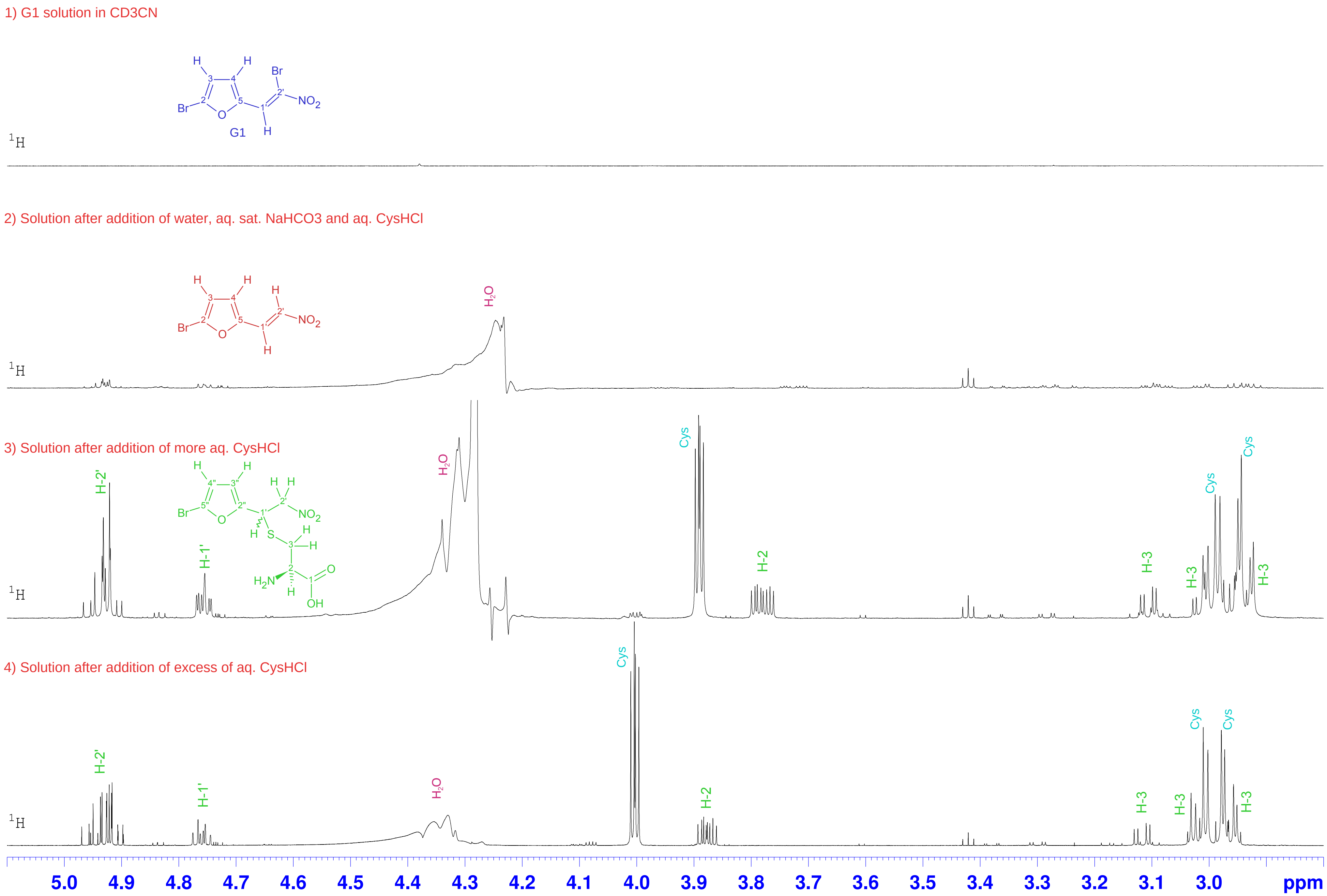

Stacked ^{1}H NMR spectra: addition of G1 to MHB solution containing CysHCl in H_2O/D_2O (5:1)

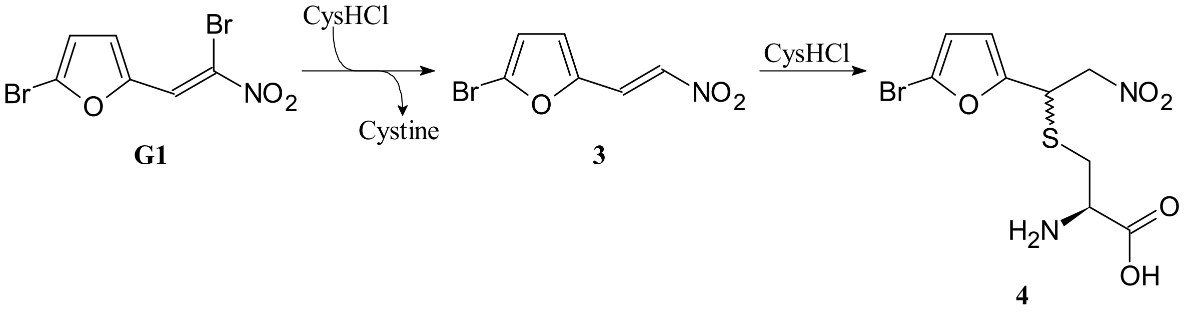

In a cation-adjusted Mueller-Hinton II broth (MHB, 20 mg) aqueous (0.5 mL H_2O and 0.1 mL D_2O) medium, cysteine hydrochloride (1.8 mg, 11.4 μmol) solution in H_2O (100 μL) was added, followed by addition of **G1** (15 μg, 0.050 μmol) solution in DMSO-d_6 (10 μL).

^{1}H NMR spectra (700.1 MHz): addition of G1 solution to aq. cysteine solution in aq. MHB solution – expansion from -0.5 ppm to +11 ppm

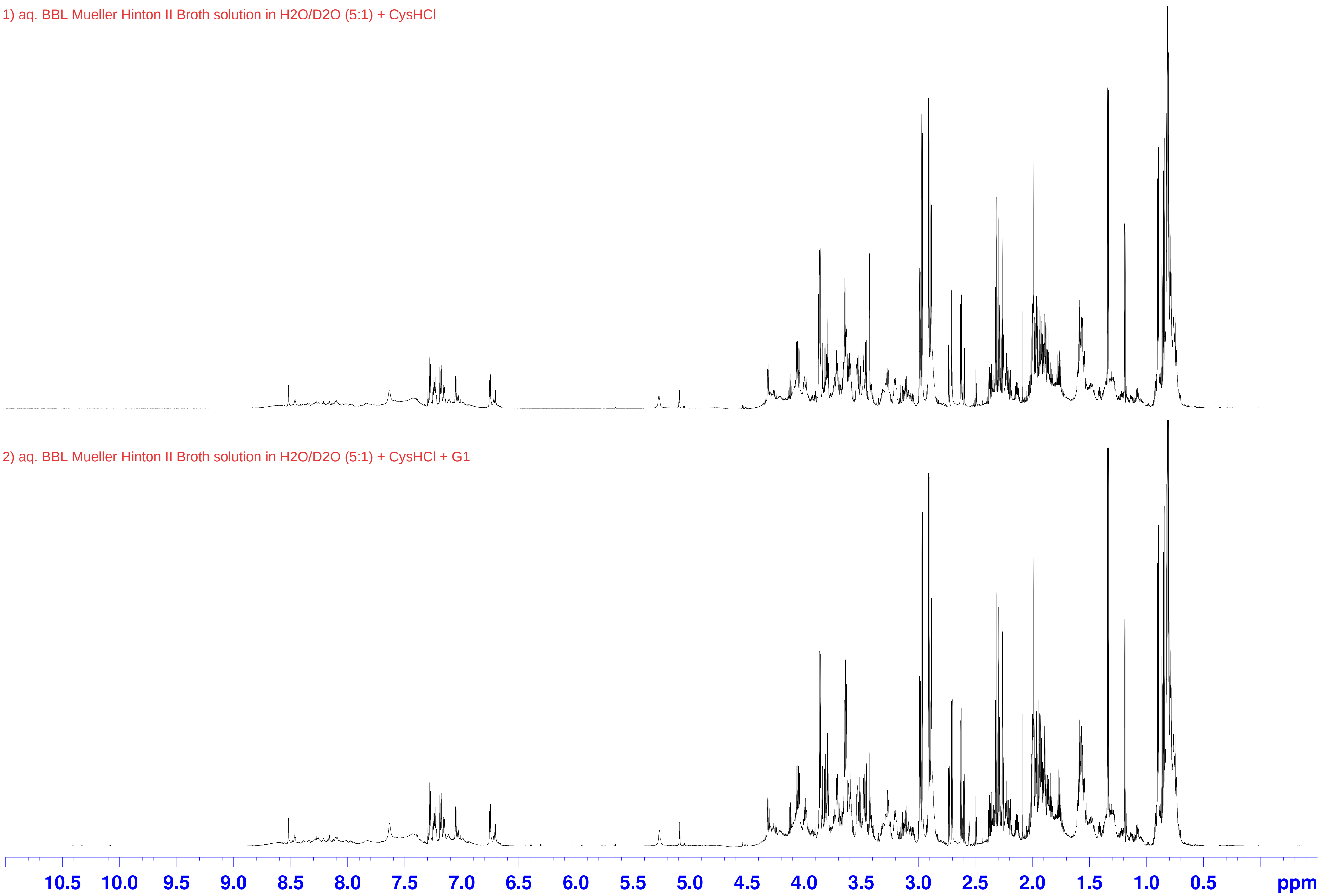

^{1}H NMR spectra (700.1 MHz): addition of G1 solution to aq. cysteine solution in aq. MHB solution – expansion from -0.5 ppm to +11 ppm, vertical scale ×8

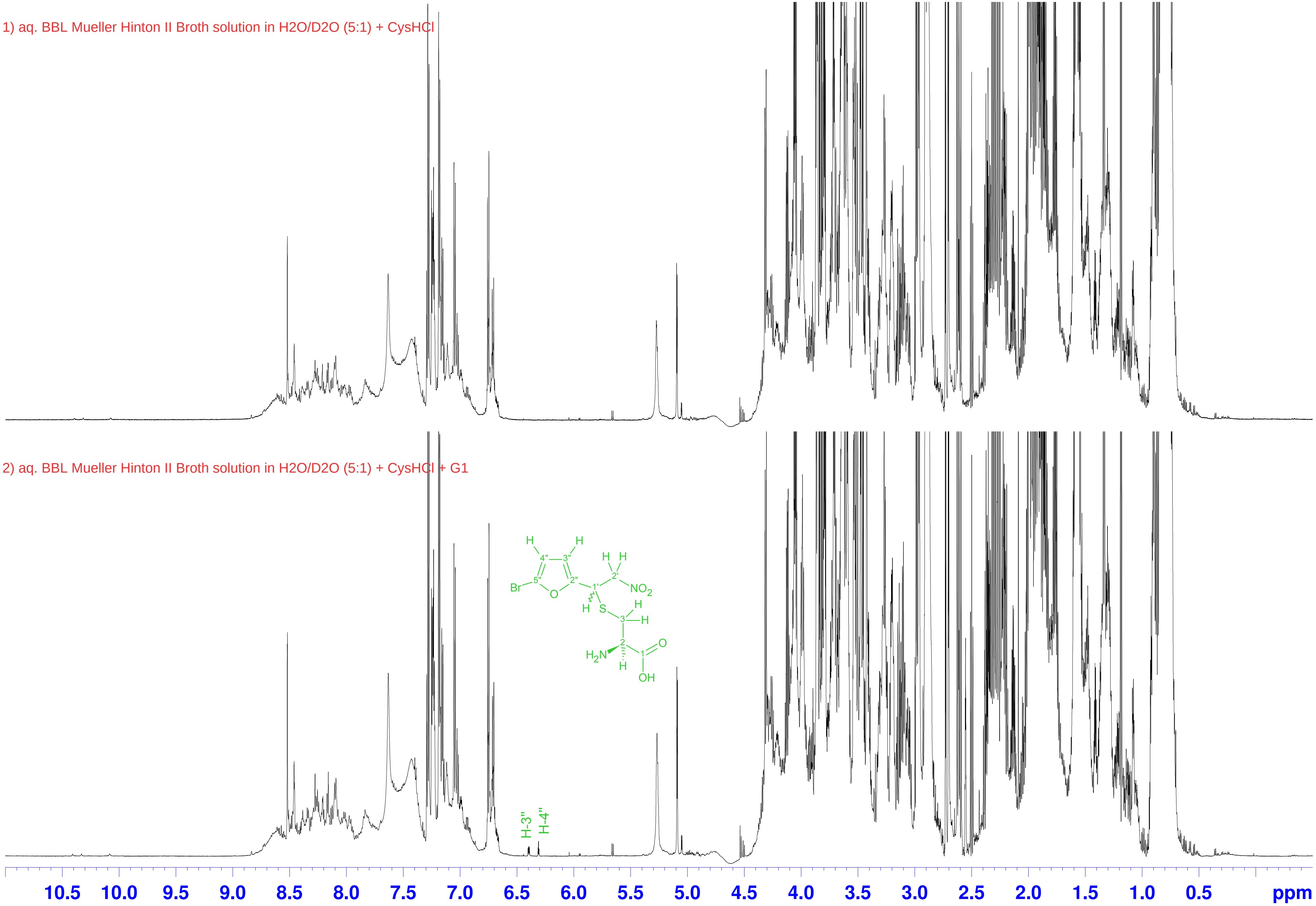

^{1}H NMR spectra (700.1 MHz): addition of G1 solution to aq. cysteine solution in aq. MHB solution – expansion from +6.1 ppm to +9.4 ppm, vertical scale ×32

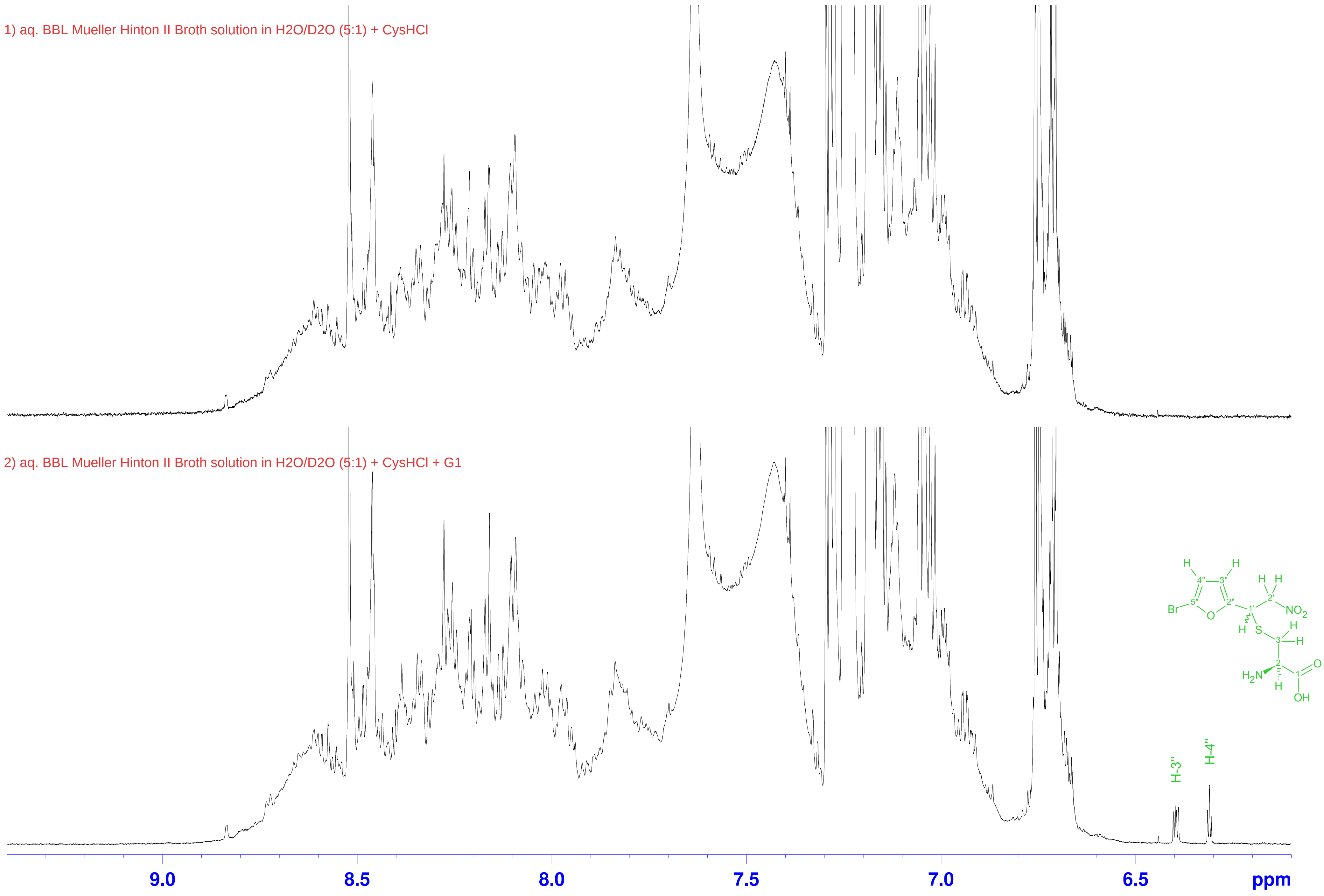

^{1}H NMR spectra (700.1 MHz): addition of G1 solution to aq. cysteine solution in aq. MHB solution – expansion from +6.1 ppm to +6.8 ppm, vertical scale ×32

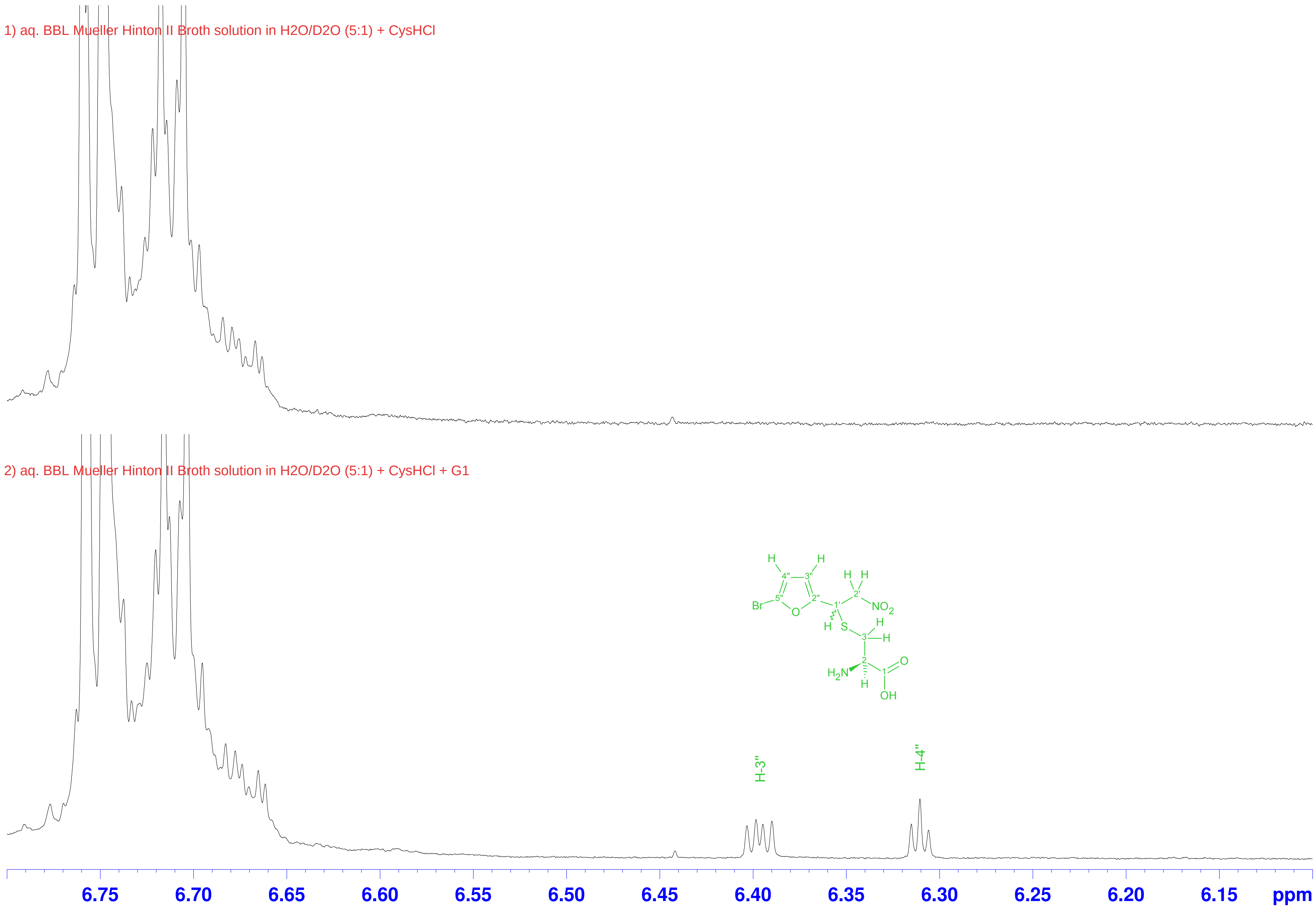

^{1}H NMR spectra (700.1 MHz): addition of G1 solution to aq. cysteine solution in aq. MHB solution – expansion from +0.5 ppm to +5.5 ppm, vertical scale ×1

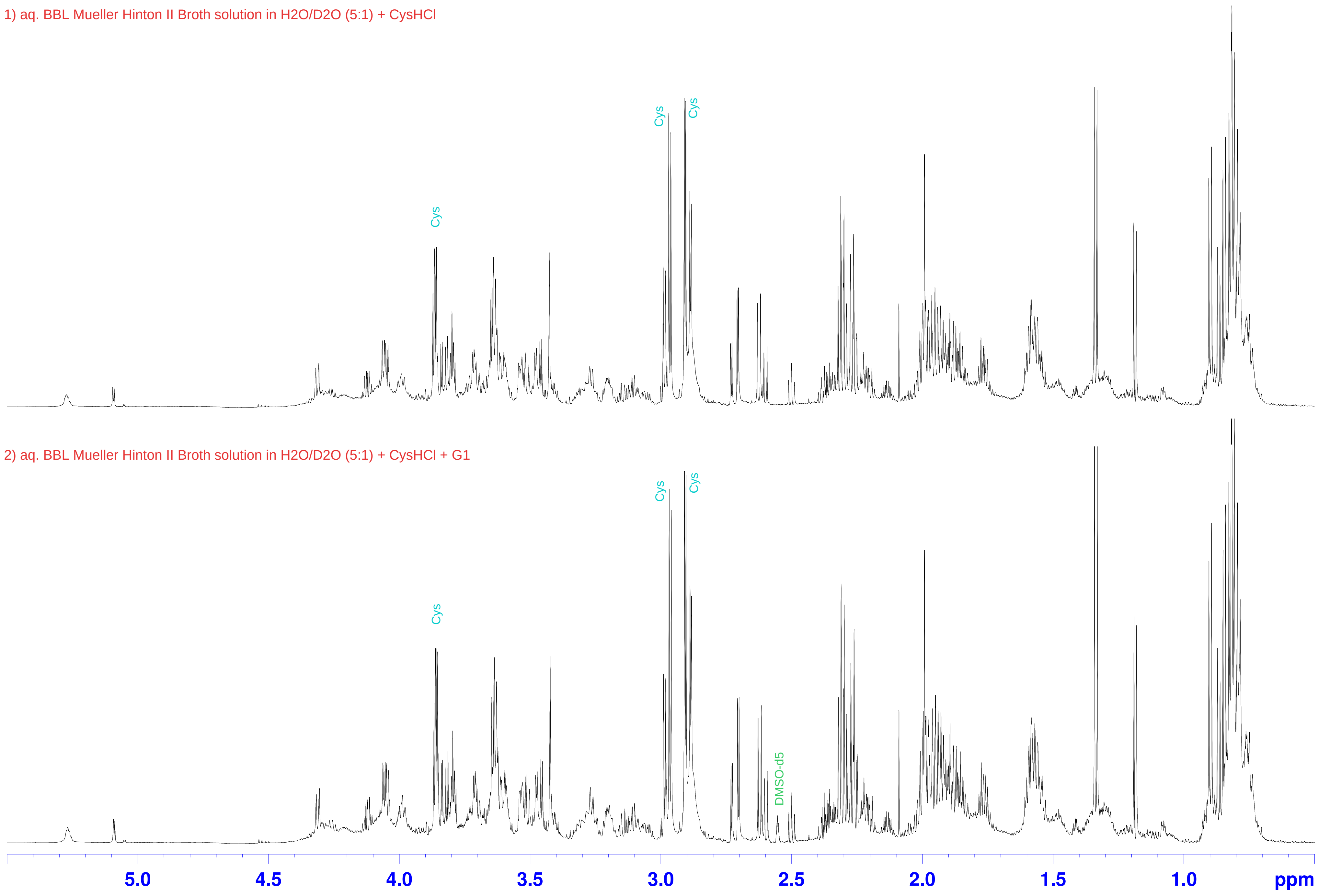

Stacked ^{1}H NMR spectra: addition of CysHCl to MHB solution containing G1 in H_2O/D_2O (1:1)

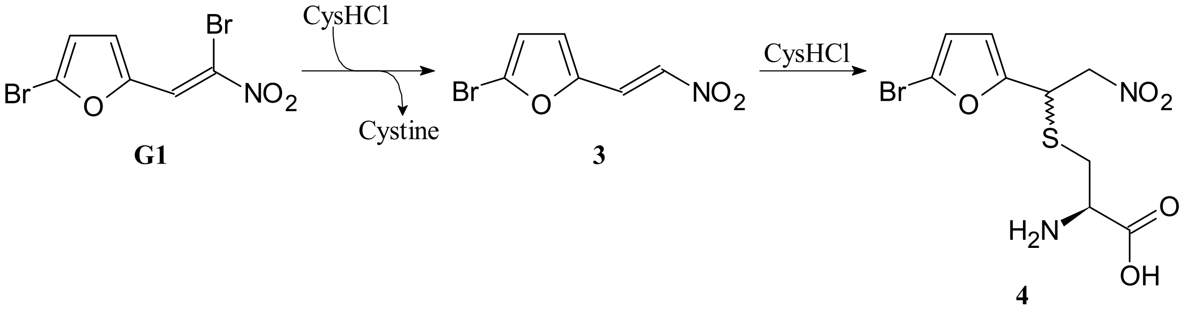

In a cation-adjusted Mueller-Hinton II broth (MHB, 20 mg) aqueous (0.4 mL H_2O and 0.4 mL D_2O) medium, **G1** (0.40 mg, 1.3 μmol) solution in DMSO-d_6 (40 μL) was added, followed by addition of cysteine hydrochloride (1.4 mg, 8.9 μmol) solution in H_2O (60 μL).

^{1}H NMR spectra (700.1 MHz): addition of CysHCl to MHB solution containing G1 in H_2O/D_2O (1:1) – expansion from -0.5 ppm to +11.0 ppm

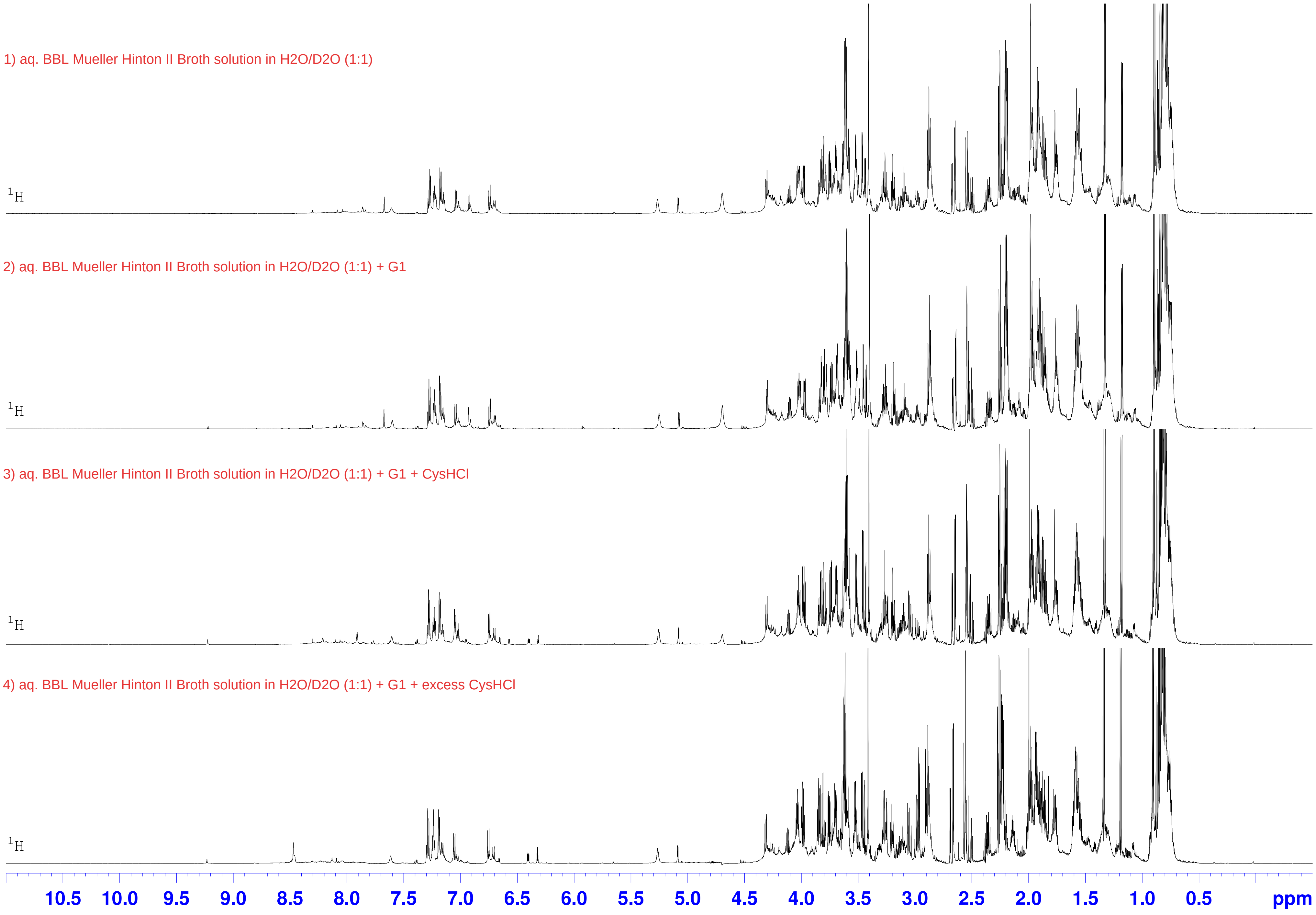

^{1}H NMR spectra (700.1 MHz): addition of CysHCl to MHB solution containing G1 in H_2O/D_2O (1:1) – expansion from 6.1 ppm to 9.4 ppm, vertical scale ×4

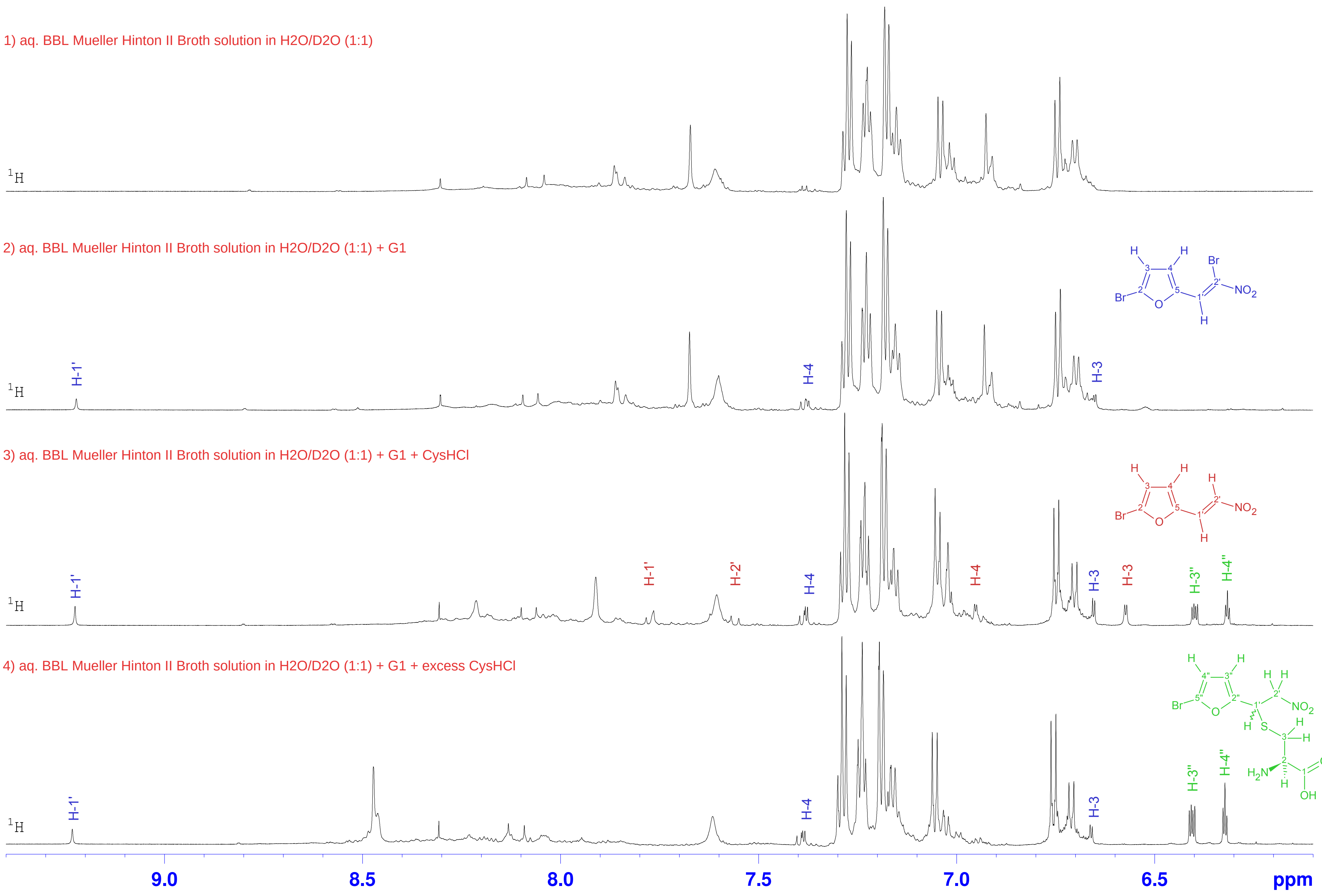

^{1}H NMR spectra (700.1 MHz): addition of CysHCl to MHB solution containing G1 in H_2O/D_2O (1:1) – expansion from 0.5 ppm to 5.5 ppm, vertical scale ×1

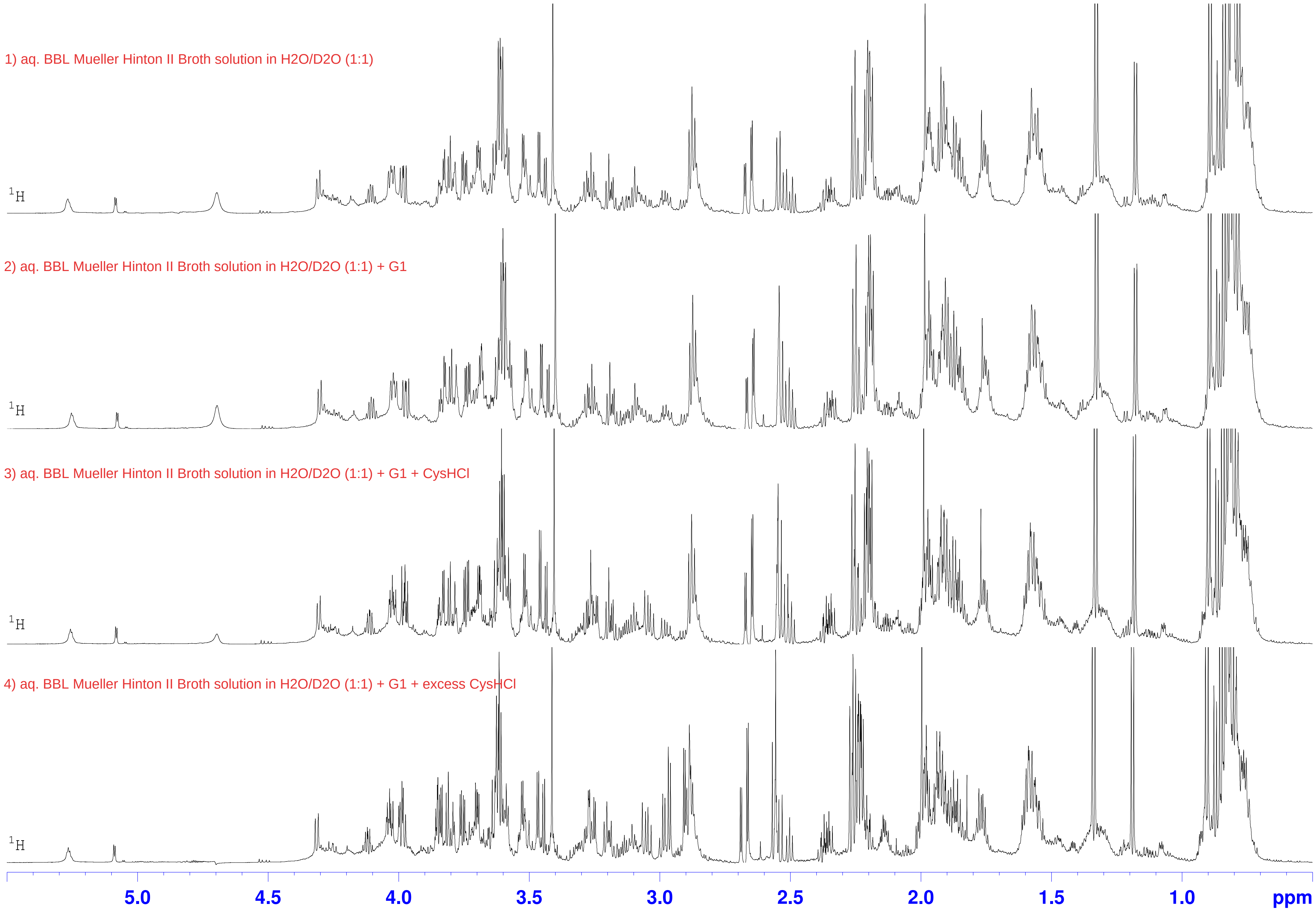

^{1}H NMR spectra (700.1 MHz): addition of CysHCl to MHB solution containing G1 in H_2O/D_2O (1:1) – expansion from 2.4 ppm to 4.4 ppm, vertical scale ×1

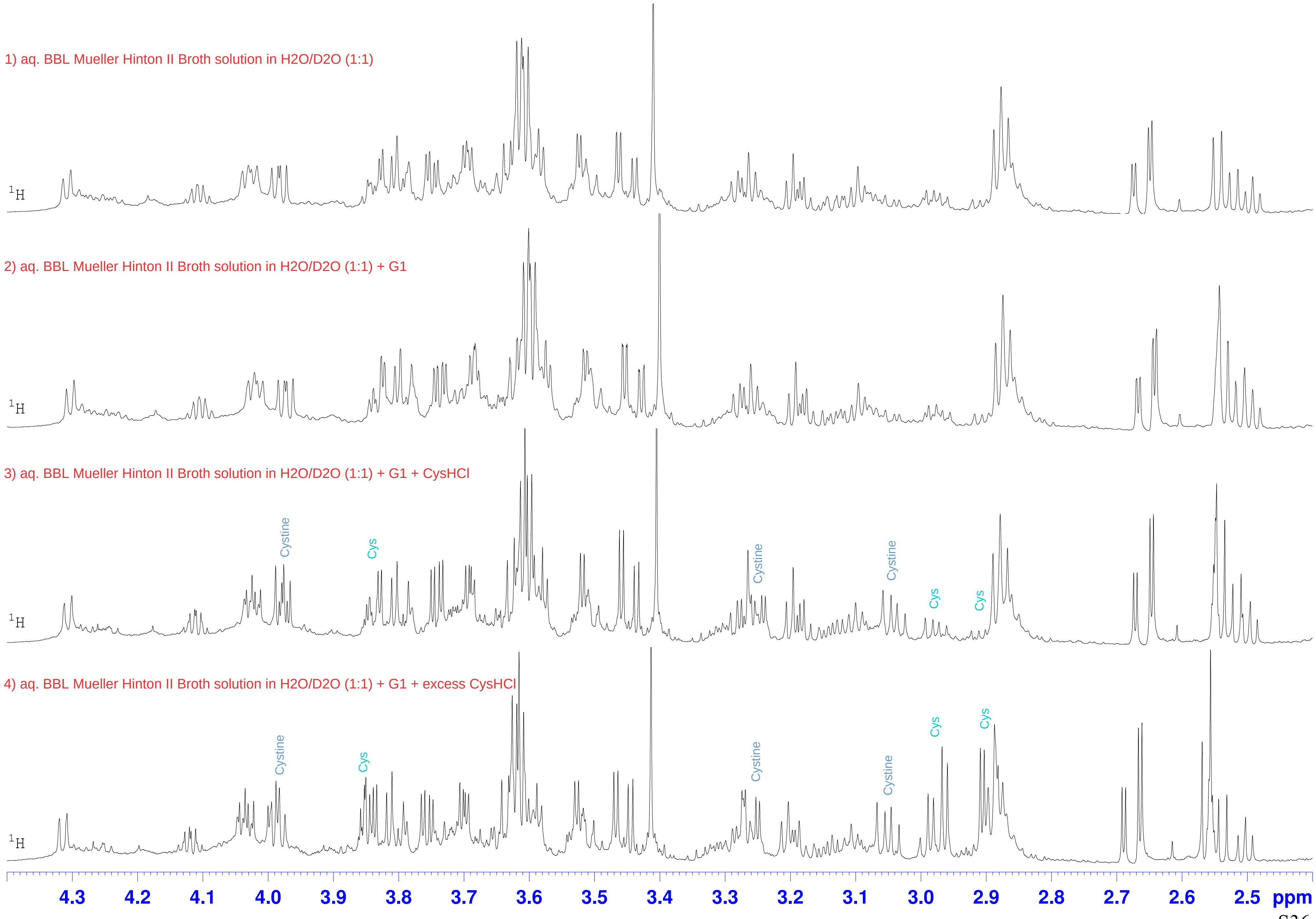

^{1}H NMR spectra (700.1 MHz): addition of CysHCl to MHB solution containing G1 in H_2O/D_2O (1:1) – expansion from 0.6 ppm to 2.45 ppm, vertical scale ×1

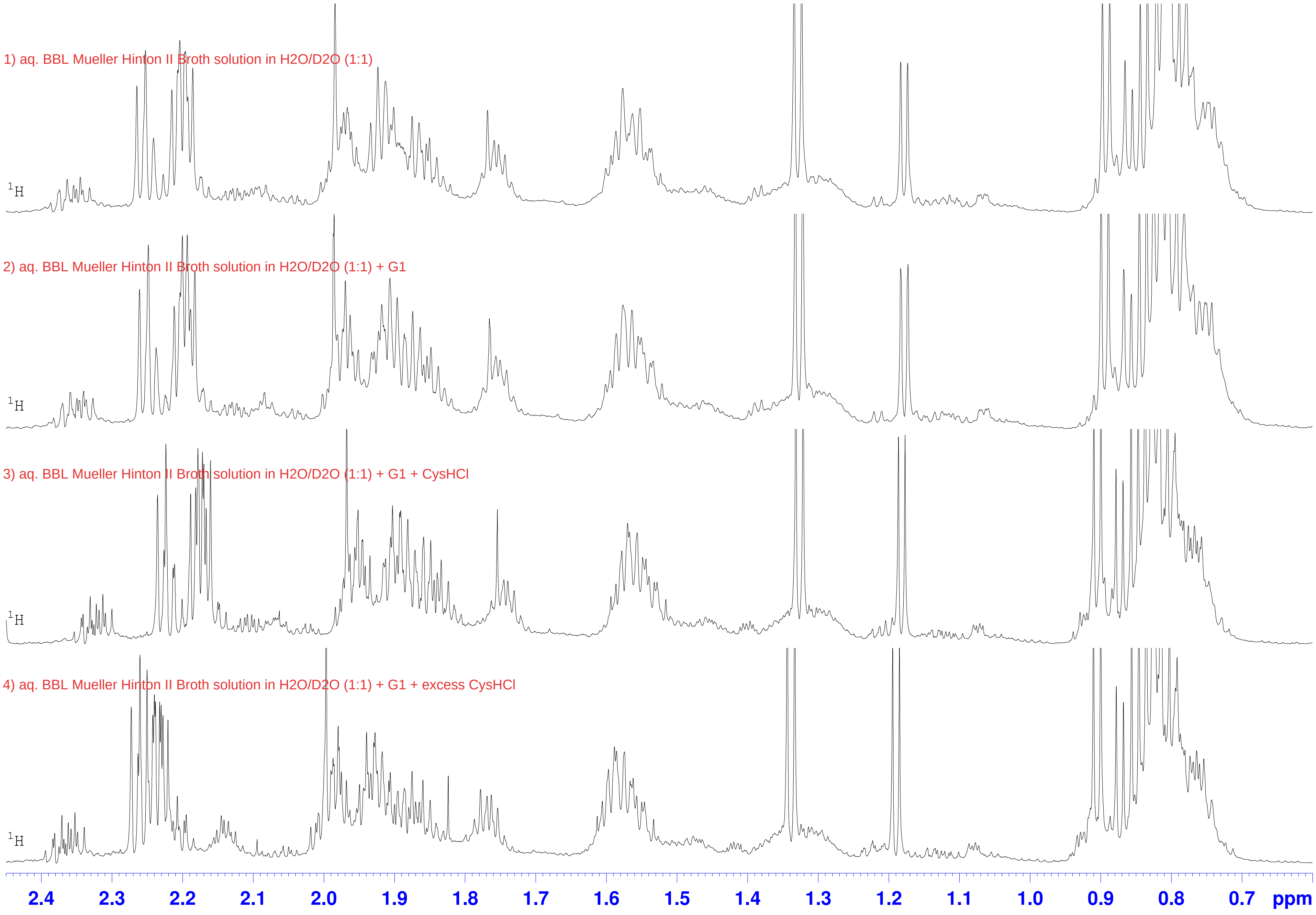

Spectra of 2-bromo-5-(2-nitroethenyl)furan (3) solution in CD_3CN/H_2O

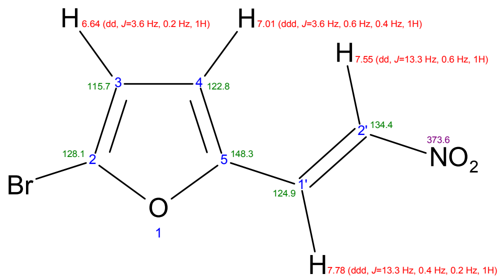

^{1}H NMR (700.1 MHz, CD_3CN/H_2O 1:1, 20.0 °C) δ: 7.78 (ddd, $^3J_{HH}$ = 13.3 Hz, $^4J_{HH}$ = 0.4 Hz, $^5J_{HH}$ = 0.2 Hz, 1H, C*H*-1'); 7.55 (dd, $^3J_{HH}$ = 13.3 Hz, $^5J_{HH}$ = 0.6 Hz, 1H, C*H*-2'); 7.01 (ddd, $^3J_{HH}$ = 3.6 Hz, $^5J_{HH}$ = 0.6 Hz, $^4J_{HH}$ = 0.4 Hz, 1H, C*H*-4); 6.64 (dd, $^3J_{HH}$ = 3.6 Hz, $^5J_{HH}$ = 0.2 Hz, C*H*-3). ^{13}C{^{1}H} NMR (176.0 MHz, CD_3CN/H_2O 1:1, 20.0 °C) δ: 148.3 (*C*-5); 134.4 (*C*H-2'); 128.1 (*C*-2); 124.9 (*C*H-1'); 122.8 (*C*H-4); 115.7 (*C*H-3). ^{15}N NMR (70.9 MHz, CD_3CN/H_2O 1:1, 20.0 °C) δ: 373.4 (*N*O_2).

^{1}H NMR spectrum (700.1 MHz) of G1 and 2-bromo-5-(2-nitroethenyl)furan (3) mixture in CD_3CN/H_2O (1:1)

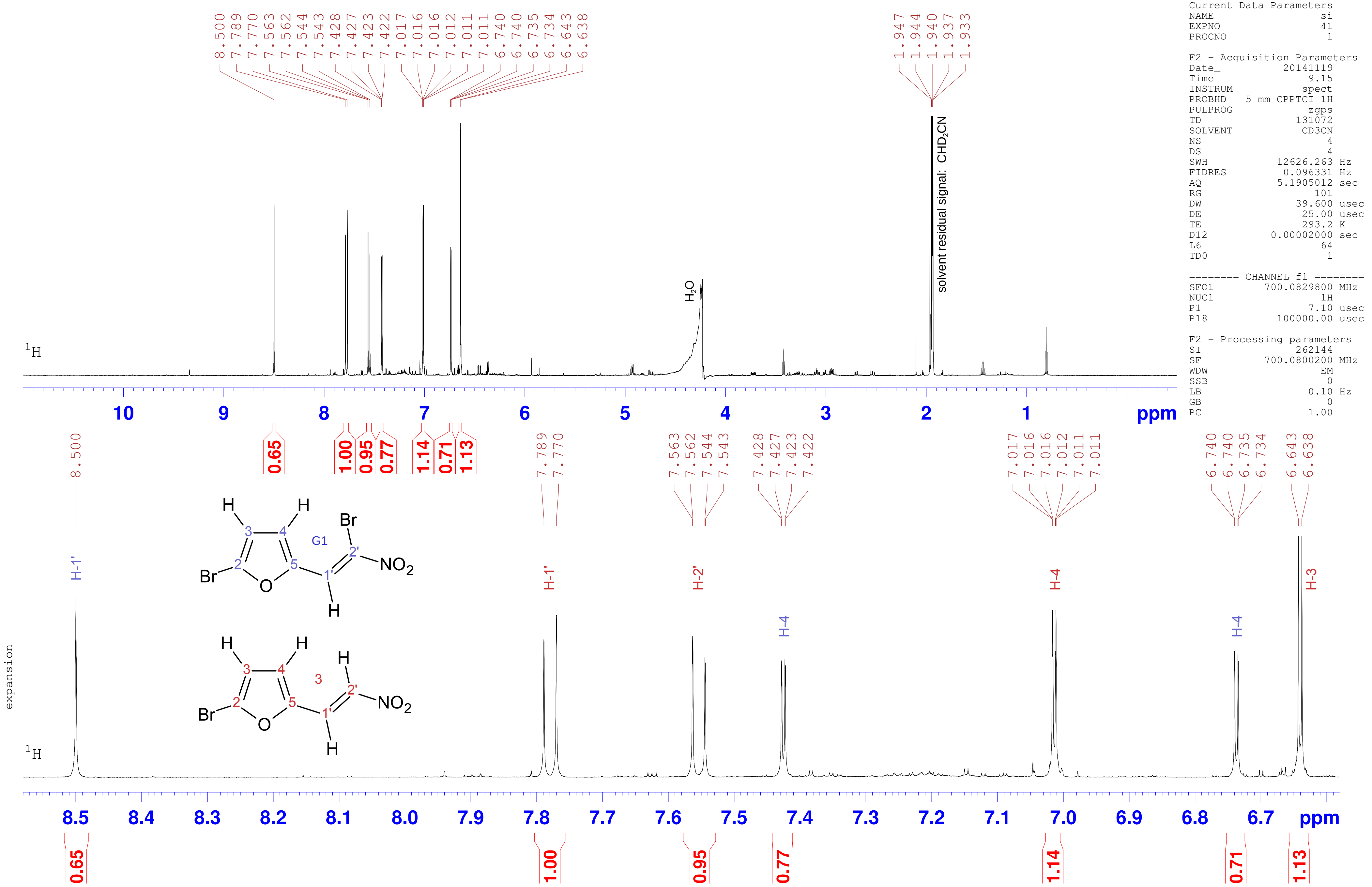

$^{13}C\{^{1}H\}$ and DEPT-135 NMR spectra (176.0 MHz) of G1 and 2-bromo-5-(2-nitroethenyl)furan (3) mixture in CD_3CN/H_2O (1:1)

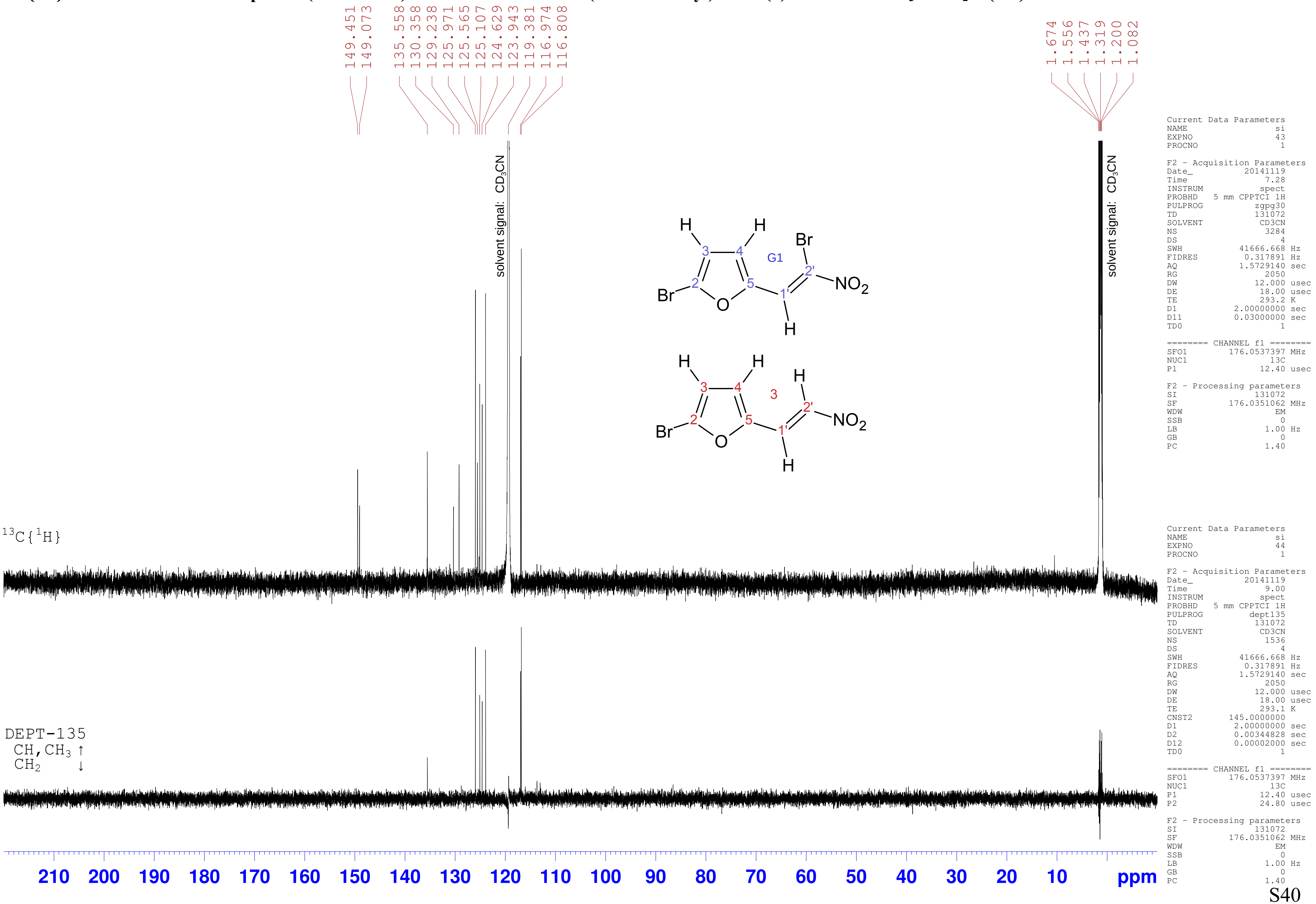

$^{13}C\{^1H\}$ and DEPT-135 NMR spectra (176.0 MHz) of G1 and 2-bromo-5-(2-nitroethenyl)furan (3) mixture in CD_3CN/H_2O (1:1) – expansion from +115.0 ppm to +152.0 ppm

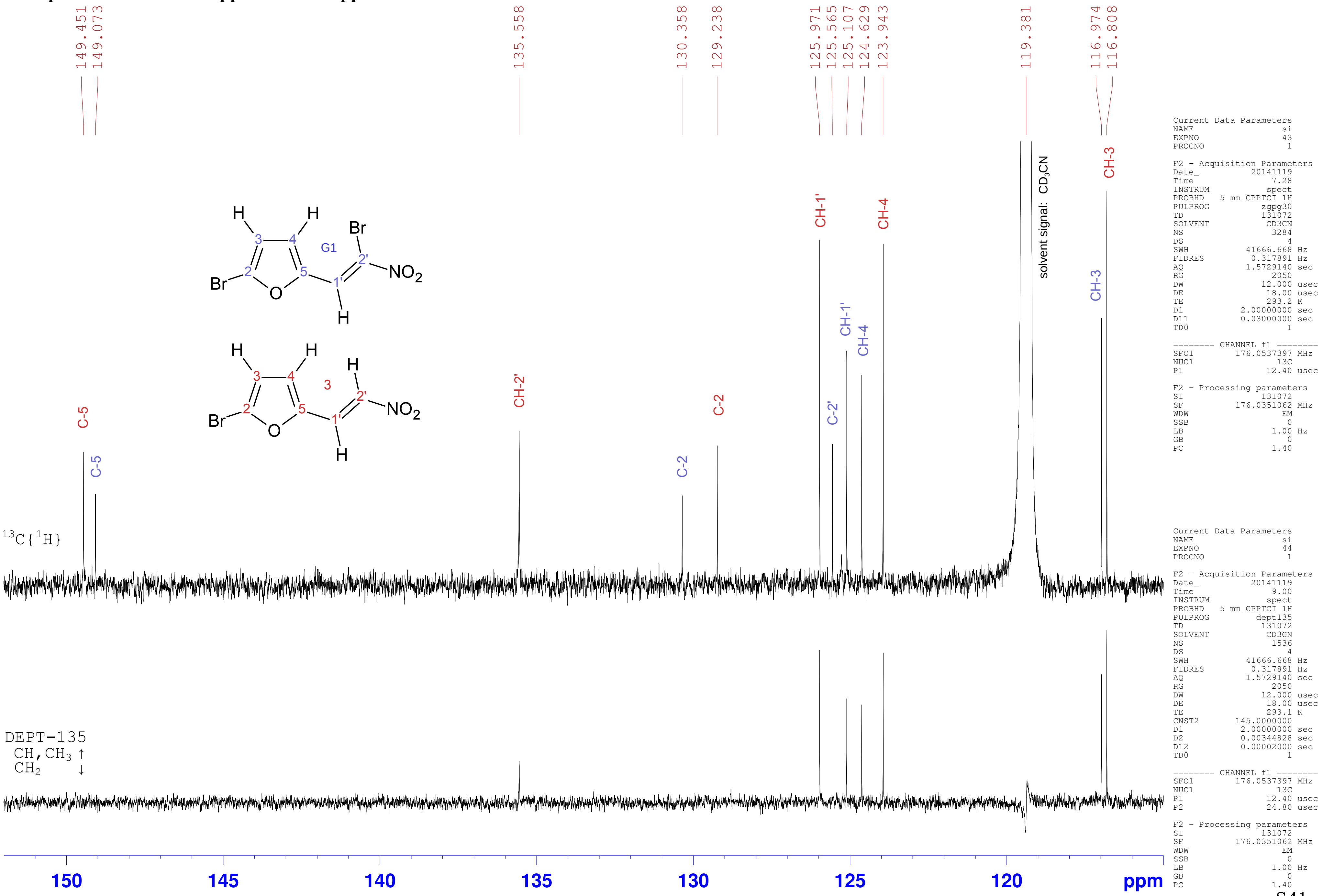

^{1}H-^{15}N HMBC NMR spectrum (700.1 MHz, 70.9 MHz) of G1 and 2-bromo-5-(2-nitroethenyl)furan (3) mixture in CD_3CN/H_2O (1:1)

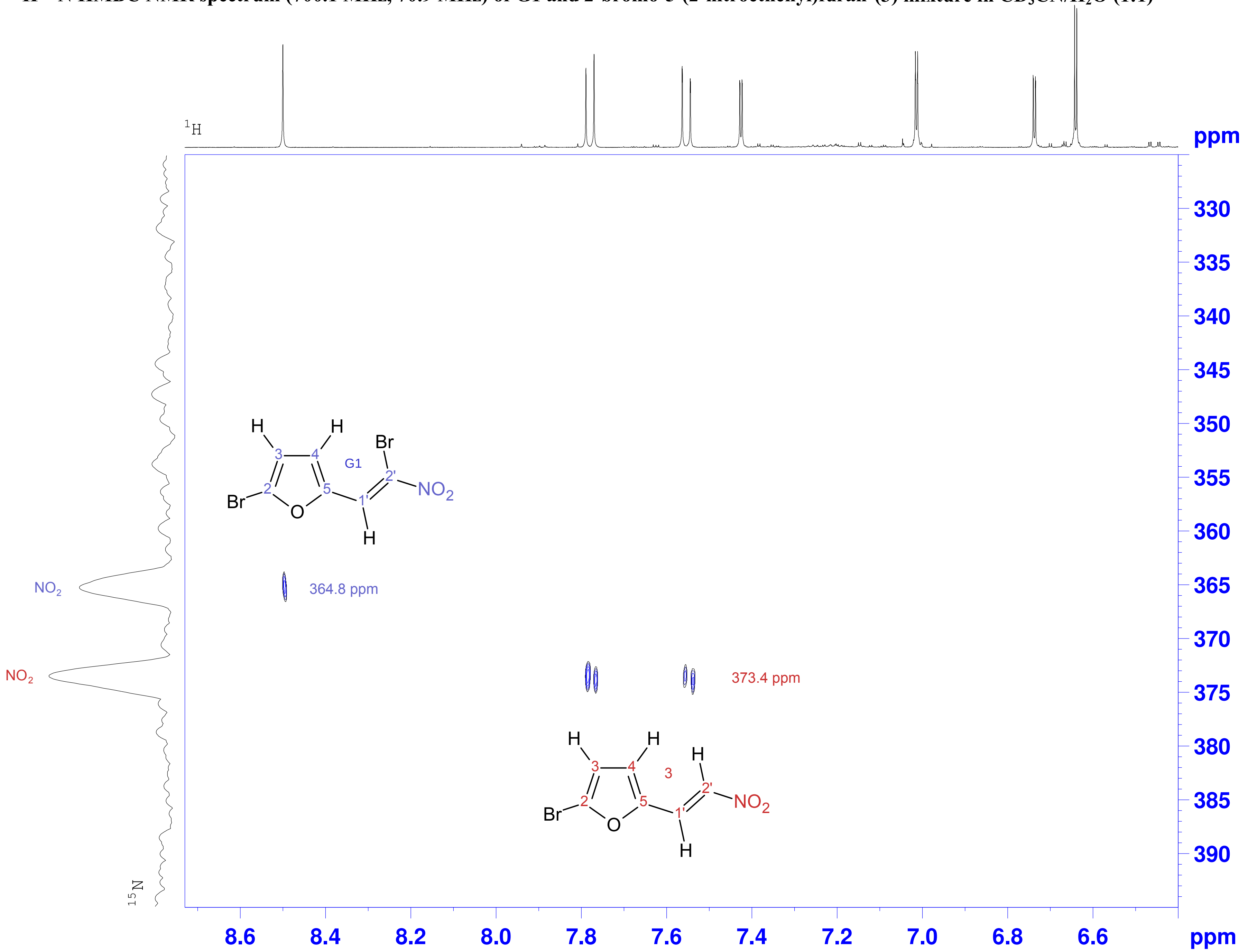

Current Data Parameters
NAME si
EXPNO 510
PROCNO 1

F2 - Acquisition Parameters
Date_ 20141119
Time 10.31
INSTRUM spect
PROBHD 5 mm CPPTCI 1H
PULPROG hmbcgplpndprqf
TD 4096
SOLVENT CD3CN
NS 160
DS 16
SWH 6313.131 Hz
FIDRES 1.541292 Hz
AQ 0.3244532 sec
RG 2050
DW 79.200 usec
DE 25.00 usec
TE 293.1 K
CNST2 95.0000000
CNST13 7.0000000
D0 0.00000300 sec
D1 1.29999995 sec
D2 0.00526316 sec
D6 0.07142857 sec
D11 0.03000000 sec
D12 0.00002000 sec
D16 0.00020000 sec
IN0 0.00008810 sec

======== CHANNEL f1 ========
SFO1 700.0829806 MHz
NUC1 1H
P1 7.10 usec
P2 14.20 usec

F1 - Acquisition parameters
ND0 2
TD 27
SFO1 70.96403 MHz
FIDRES 210.198853 Hz
SW 79.975 ppm
FnMODE QF

F2 - Processing parameters
SI 4096
SF 700.0800200 MHz
WDW QSINE
SSB 2
LB 0.00 Hz
GB 0
PC 1.40

F1 - Processing parameters
SI 1024
MC2 QF
SF 70.9384928 MHz
WDW QSINE
SSB 2
LB 0.00 Hz
GB 0

Spectra of 2-bromo-5-(2-nitroethenyl)furan (3) solution in CD_3OD

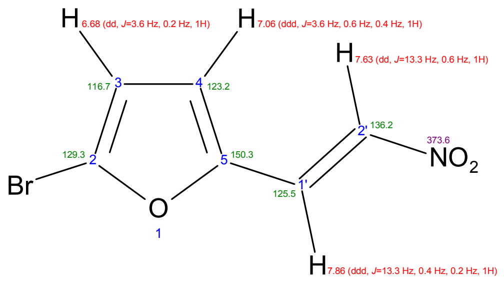

^{1}H NMR (700.1 MHz, CD_3OH, 20.0 °C) δ: 7.86 (ddd, $^3J_{HH}$ = 13.3 Hz, $^4J_{HH}$ = 0.4 Hz, $^5J_{HH}$ = 0.2 Hz, 1H, C<u>*H*</u>-1'); 7.63 (dd, $^3J_{HH}$ = 13.3 Hz, $^5J_{HH}$ = 0.6 Hz, 1H, C<u>*H*</u>-2'); 7.06 (ddd, $^3J_{HH}$ = 3.6 Hz, $^5J_{HH}$ = 0.6 Hz, $^4J_{HH}$ = 0.4 Hz, 1H, C<u>*H*</u>-4); 6.68 (dd, $^3J_{HH}$ = 3.6 Hz, $^5J_{HH}$ = 0.2 Hz, C<u>*H*</u>-3). ^{13}C{^{1}H} NMR (176.0 MHz, CD_3OH, 20.0 °C) δ: 150.3 (<u>*C*</u>-5); 136.2 (<u>*C*</u>H-2'); 129.3 (<u>*C*</u>-2); 125.5 (<u>*C*</u>H-1'); 123.2 (<u>*C*</u>H-4); 116.7 (<u>*C*</u>H-3). ^{15}N NMR (70.9 MHz, CD_3OH, 20.0 °C) δ: 373.6 (<u>*N*</u>O_2).

^{1}H NMR spectrum (700.1 MHz) of 2-bromo-5-(2-nitroethenyl)furan (3) solution in CD_3OD

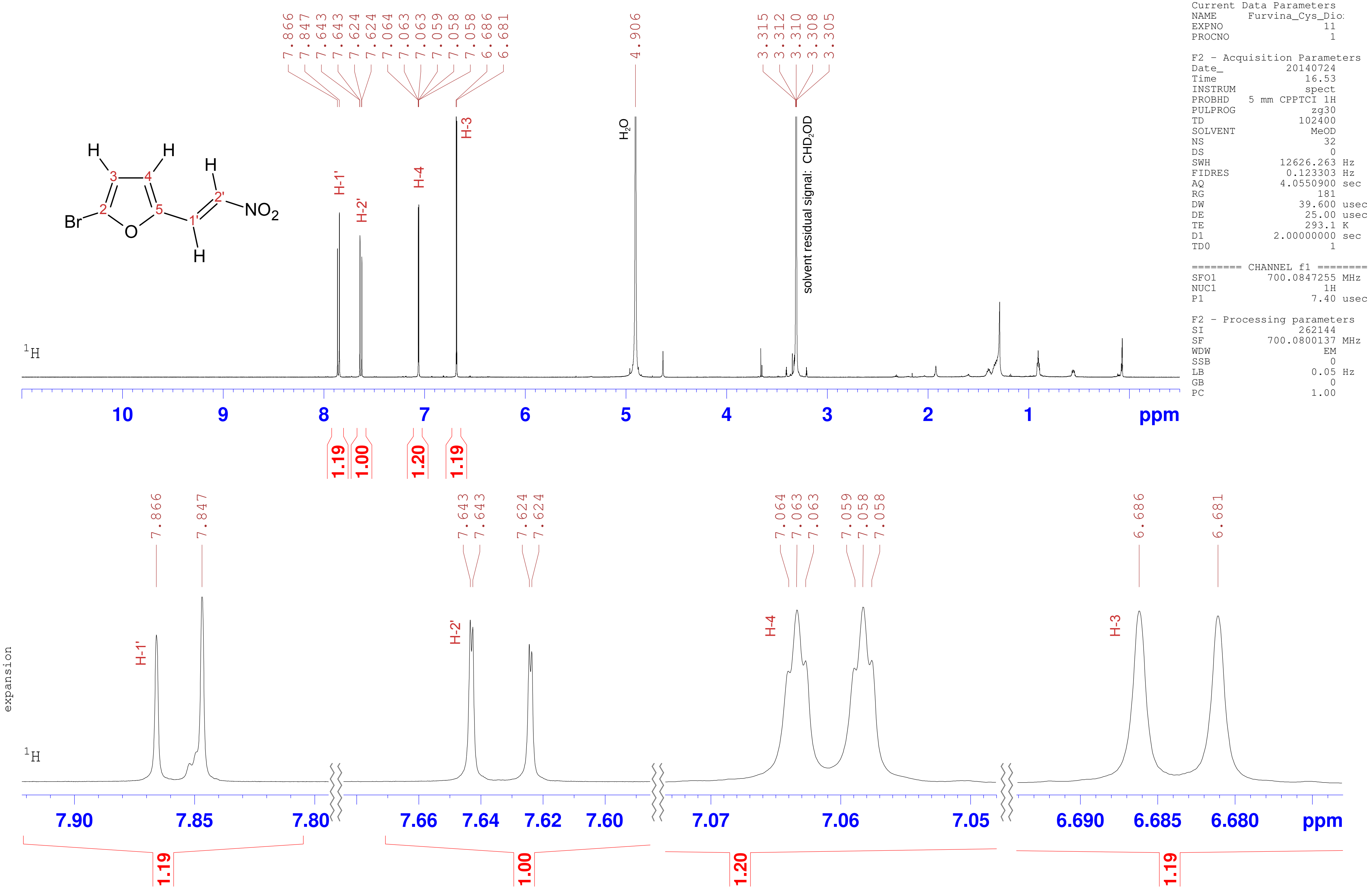

$^{13}C\{^1H\}$ and DEPT-135 NMR spectra (176.0 MHz) of 2-bromo-5-(2-nitroethenyl)furan (3) solution in CD_3OD

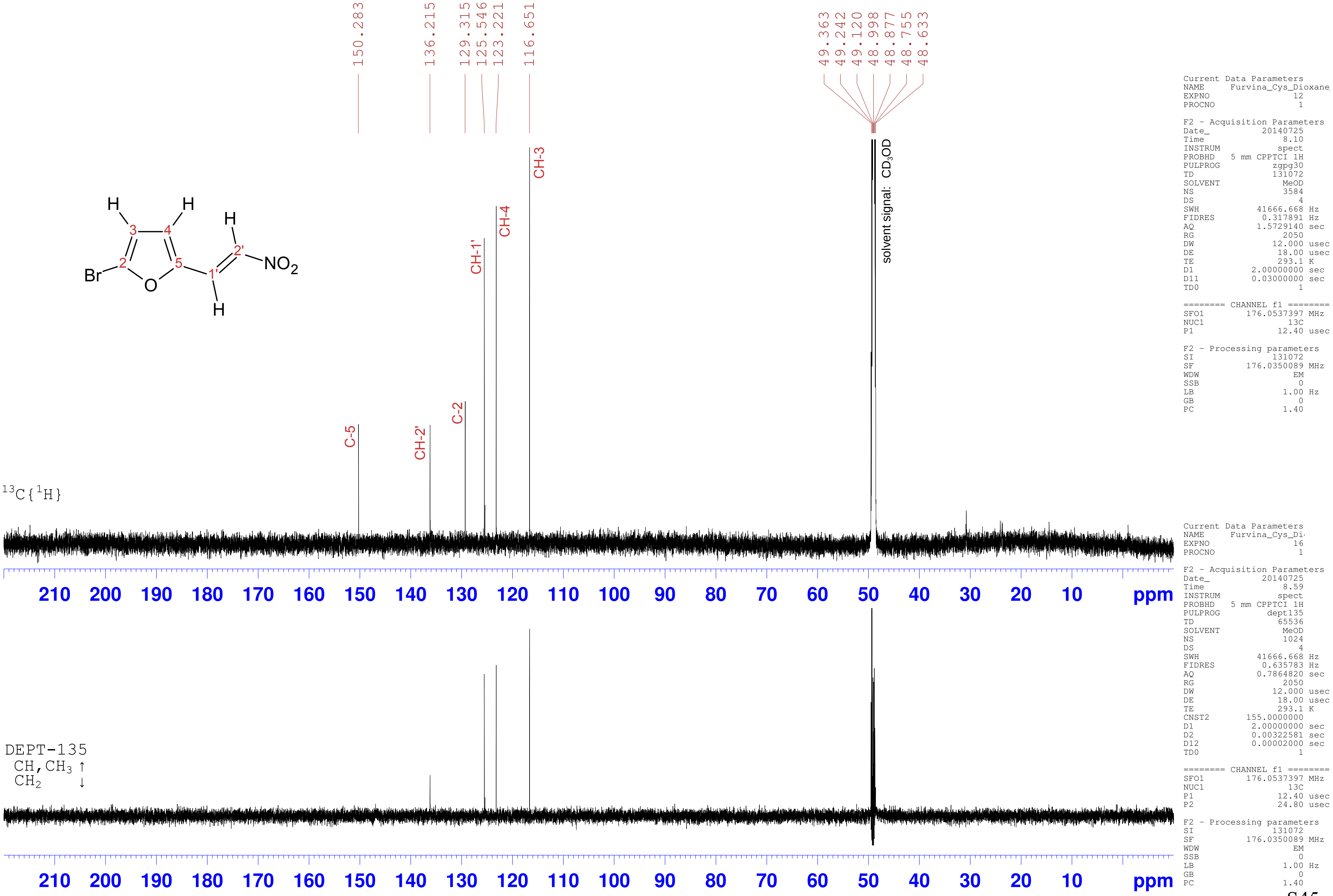

$^{13}C\{^{1}H\}$ and DEPT-135 NMR spectra (176.0 MHz) of 2-bromo-5-(2-nitroethenyl)furan (3) solution in CD_3OD – expansion from +112.0 ppm to +155.0 ppm

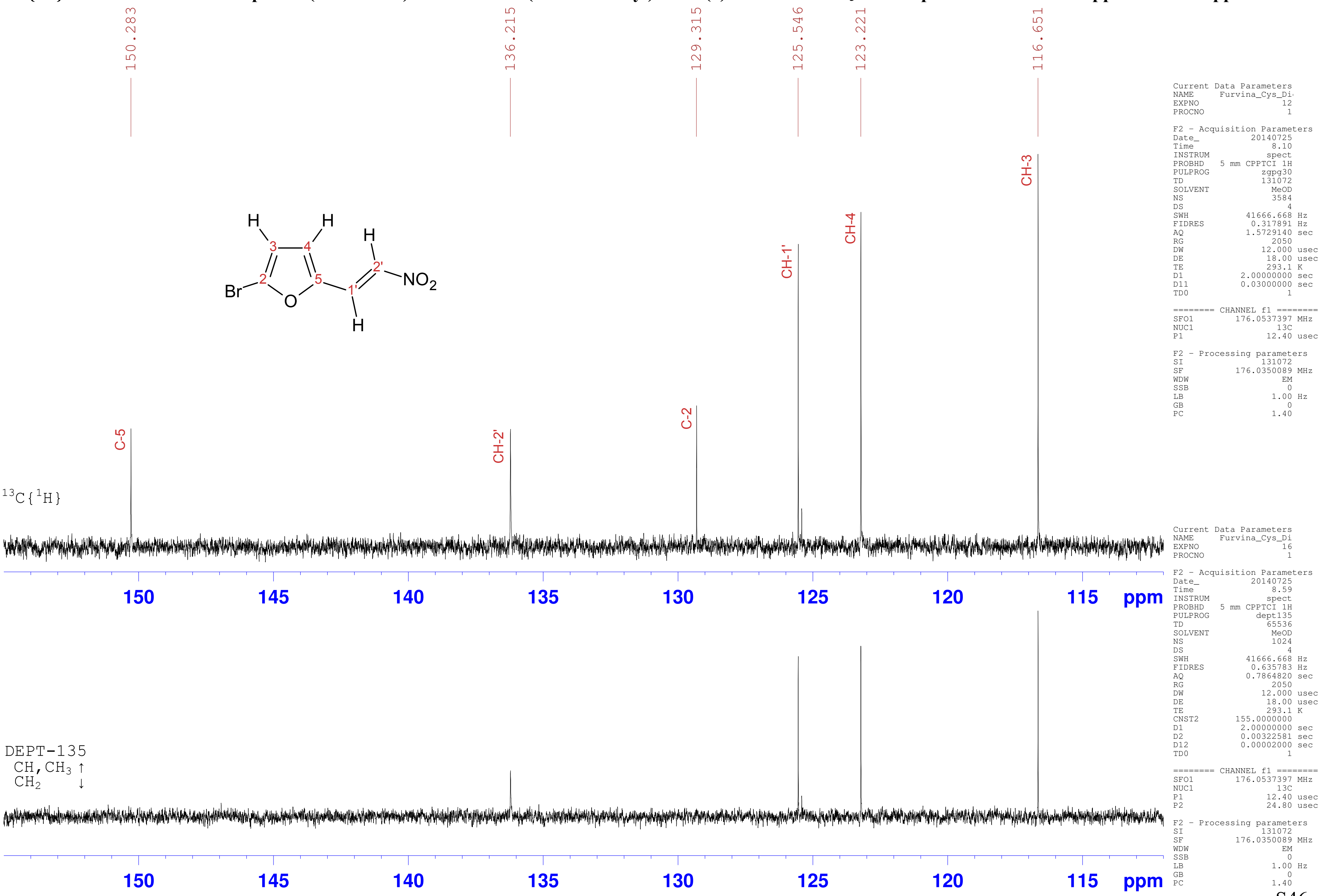

^{1}H-^{15}N HMBC NMR spectrum (700.1 MHz, 70.9 MHz) of 2-bromo-5-(2-nitroethenyl)furan (3) solution in CD_3OD

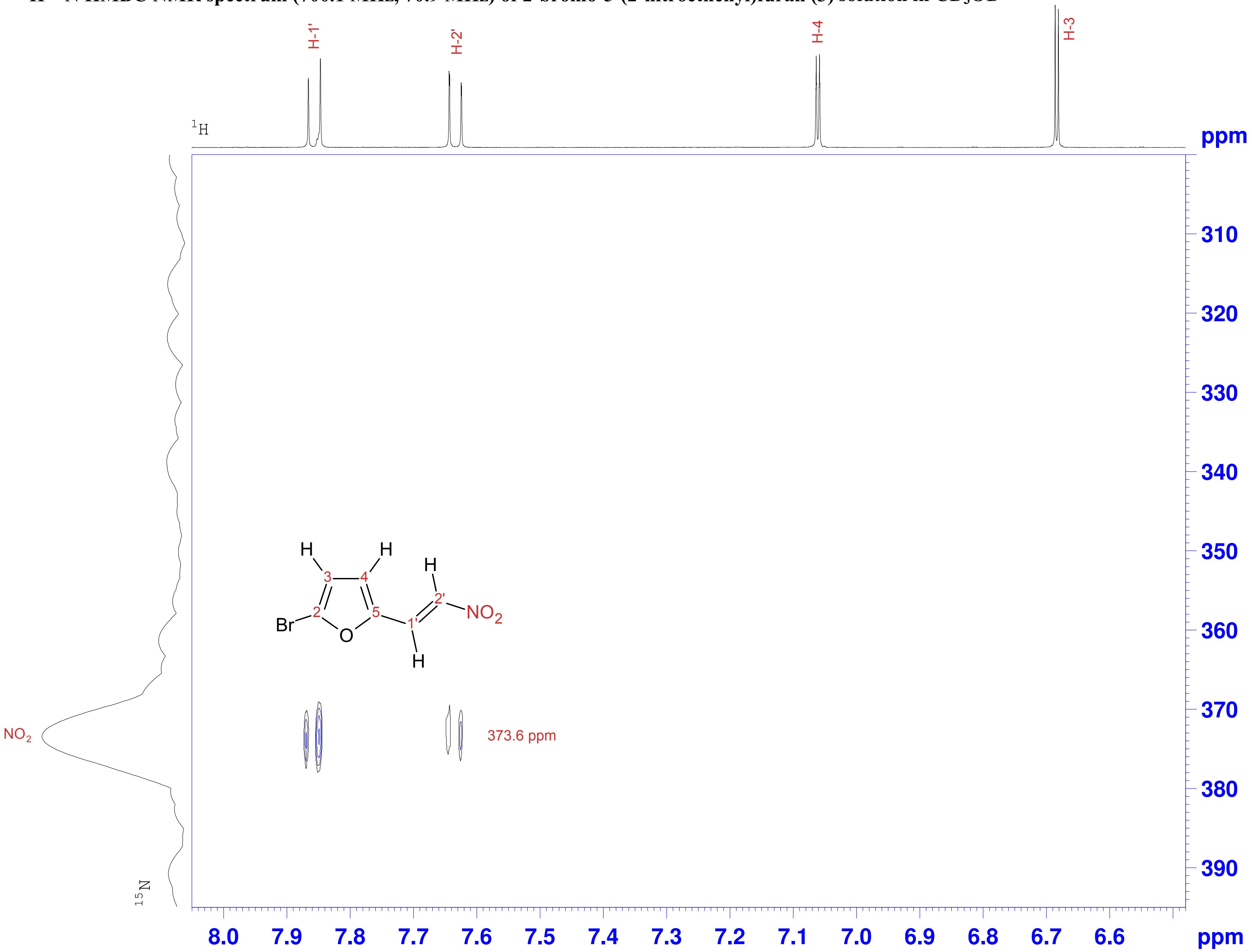


```
Current Data Parameters
NAME      Furvina_Cys_Di
EXPNO                  17
PROCNO                  1

F2 - Acquisition Parameters
Date_            20140725
Time                10.58
INSTRUM             spect
PROBHD    5 mm CPPTCI 1H
PULPROG     hmbcgplpndqf
TD                   1024
SOLVENT              MeOD
NS                    256
DS                     16
SWH              1539.409 Hz
FIDRES           1.503329 Hz
AQ              0.3326452 sec
RG                   2050
DW                324.800 usec
DE                  25.00 usec
TE                  293.1 K
CNST2          95.0000000
CNST13          7.0000000
D0             0.00000300 sec
D1             1.20000005 sec
D2             0.00526316 sec
D6             0.07142857 sec
D16            0.00020000 sec
IN0            0.00007050 sec

======== CHANNEL f1 ========
SFO1          700.0851421 MHz
NUC1                   1H
P1                   7.40 usec
P2                  14.80 usec

F1 - Acquisition parameters
ND0                     2
TD                      9
SFO1             70.96332 MHz
FIDRES         788.022095 Hz
SW                 99.942 ppm
FnMODE                 QF

F2 - Processing parameters
SI                   2048
SF            700.0800138 MHz
WDW                 QSINE
SSB                     2
LB                   0.00 Hz
GB                      0
PC                   1.40

F1 - Processing parameters
SI                   1024
MC2                    QF
SF             70.9384928 MHz
WDW                 QSINE
SSB                     2
LB                   0.00 Hz
GB                      0
```

Spectra of 2-bromo-5-[2-nitro(2-^{2}H)ethenyl]furan solution in CD_3CN/D_2O

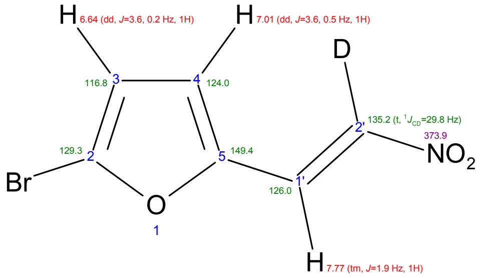

^{1}H NMR (700.1 MHz, CD_3CN/D_2O 1:1, 20.0 °C) δ: 7.77 (dm, $^{3}J_{HD}$ = 1.9 Hz, 1H, C*H*-1'); 7.01 (dd, $^{3}J_{HH}$ = 3.6 Hz, $^{4}J_{HH}$ = 0.5 Hz, 1H, C*H*-4); 6.64 (dd, $^{3}J_{HH}$ = 3.6 Hz, $^{5}J_{HH}$ = 0.2 Hz, C*H*-3). ^{13}C{^{1}H} NMR (176.0 MHz, CD_3CN/D_2O 1:1, 20.0 °C) δ: 149.4 (*C*-5); 135.2 ($^{1}J_{CD}$ = 29.8 Hz, *C*D-2'); 129.3 (*C*-2); 126.0 (*C*H-1'); 124.0 (*C*H-4); 116.8 (*C*H-3). ^{15}N NMR (70.9 MHz, CD_3CN/D_2O 1:1, 20.0 °C) δ: 373.9 (*N*O_2).

^{1}H NMR spectrum (700.1 MHz) of G1 and 2-bromo-5-[2-nitro(2-^{2}H)ethenyl]furan solution in CD_3CN/D_2O (1:1)

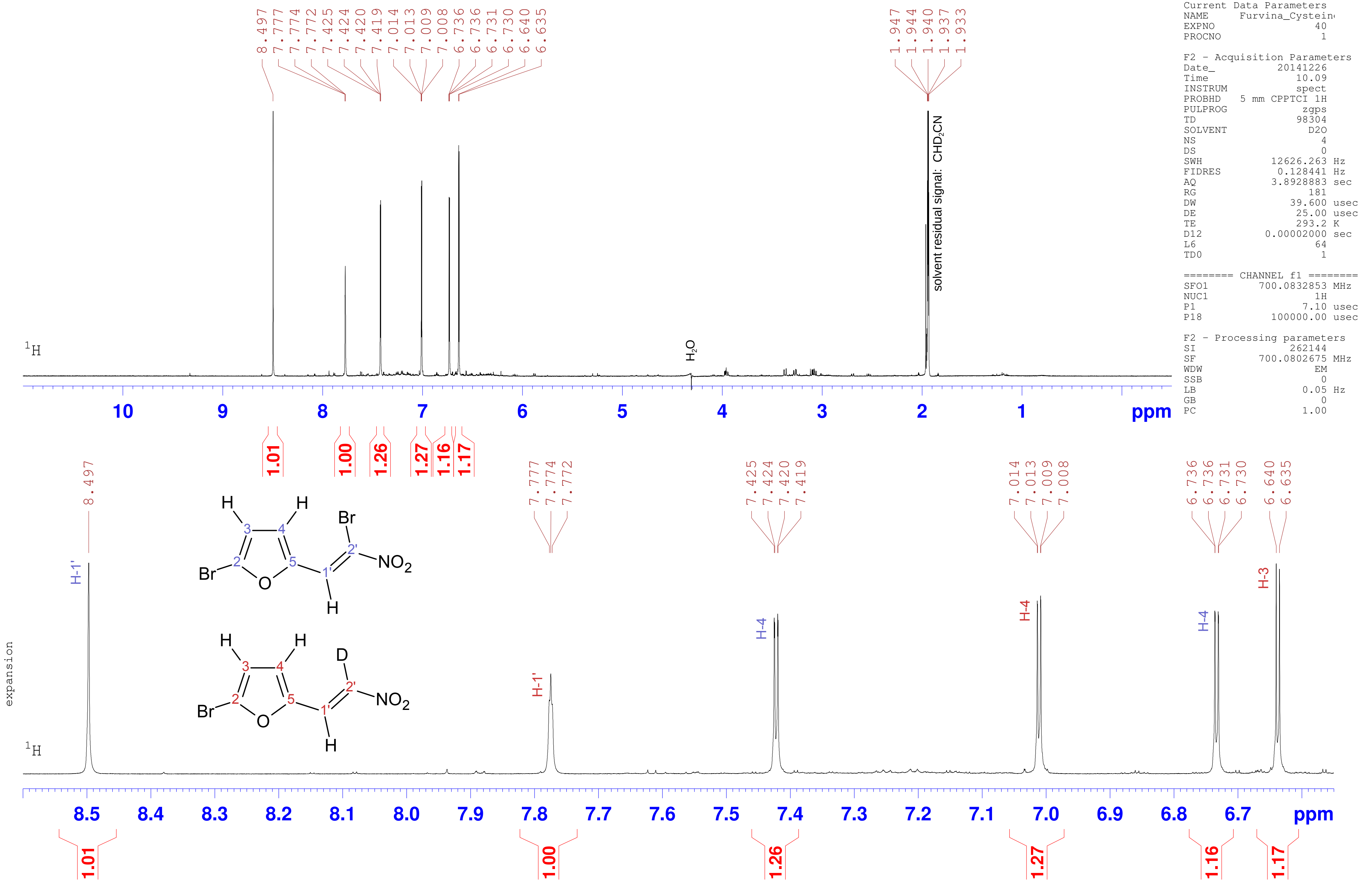

$^{13}C\{^1H\}$ and DEPT-135 NMR spectra (176.0 MHz) of G1 and 2-bromo-5-[2-nitro(2-^{2}H)ethenyl]furan solution in CD_3CN/D_2O (1:1)

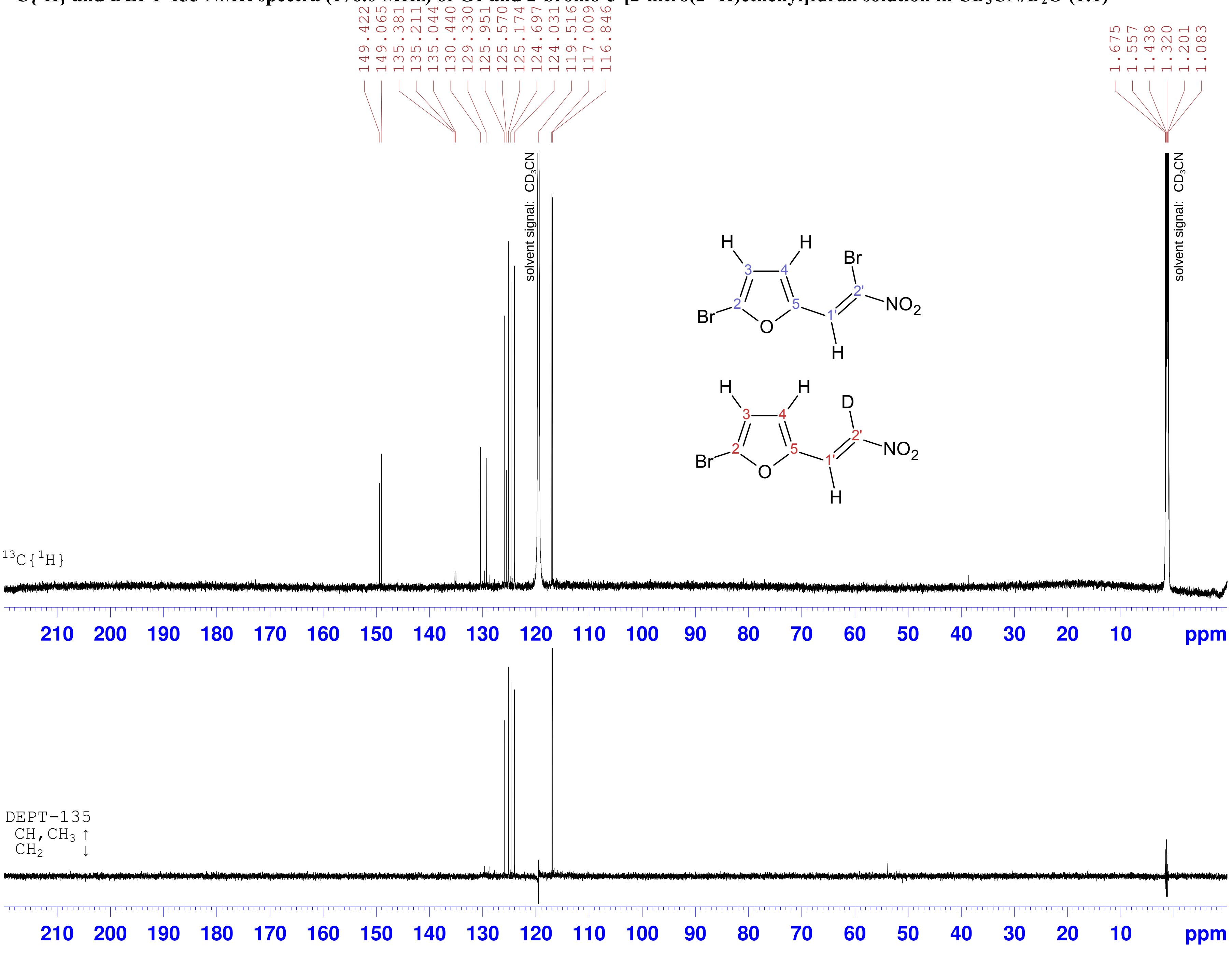

Current Data Parameters
NAME Furvina_Cysteine_CD
EXPNO 36
PROCNO 1

F2 - Acquisition Parameters
Date_ 20141224
Time 12.34
INSTRUM spect
PROBHD 5 mm CPPTCI 1H
PULPROG zgpg30
TD 131072
SOLVENT D2O
NS 32768
DS 4
SWH 41666.668 Hz
FIDRES 0.317891 Hz
AQ 1.5729140 sec
RG 2050
DW 12.000 usec
DE 18.00 usec
TE 293.1 K
D1 2.00000000 sec
D11 0.03000000 sec
TD0 1

======== CHANNEL f1 ========
SFO1 176.0537397 MHz
NUC1 13C
P1 12.40 usec

F2 - Processing parameters
SI 131072
SF 176.0351664 MHz
WDW EM
SSB 0
LB 1.00 Hz
GB 0
PC 1.40

Current Data Parameters
NAME Furvina_Cysteine_CD
EXPNO 38
PROCNO 1

F2 - Acquisition Parameters
Date_ 20141225
Time 22.02
INSTRUM spect
PROBHD 5 mm CPPTCI 1H
PULPROG dept135
TD 65536
SOLVENT D2O
NS 14336
DS 8
SWH 41666.668 Hz
FIDRES 0.635783 Hz
AQ 0.7864820 sec
RG 2050
DW 12.000 usec
DE 18.00 usec
TE 293.1 K
CNST2 160.0000000
D1 2.00000000 sec
D2 0.00312500 sec
D12 0.00002000 sec
TD0 1

======== CHANNEL f1 ========
SFO1 176.0537397 MHz
NUC1 13C
P1 12.40 usec
P2 24.80 usec

F2 - Processing parameters
SI 131072
SF 176.0351664 MHz
WDW EM
SSB 0
LB 1.00 Hz
GB 0
PC 1.40

$^{13}C\{^{1}H\}$ and DEPT-135 NMR spectra (176.0 MHz) of G1 and 2-bromo-5-[2-nitro(2-^{2}H)ethenyl]furan solution in CD_3CN/D_2O (1:1) – expansion from +115.0 ppm to +152.0 ppm

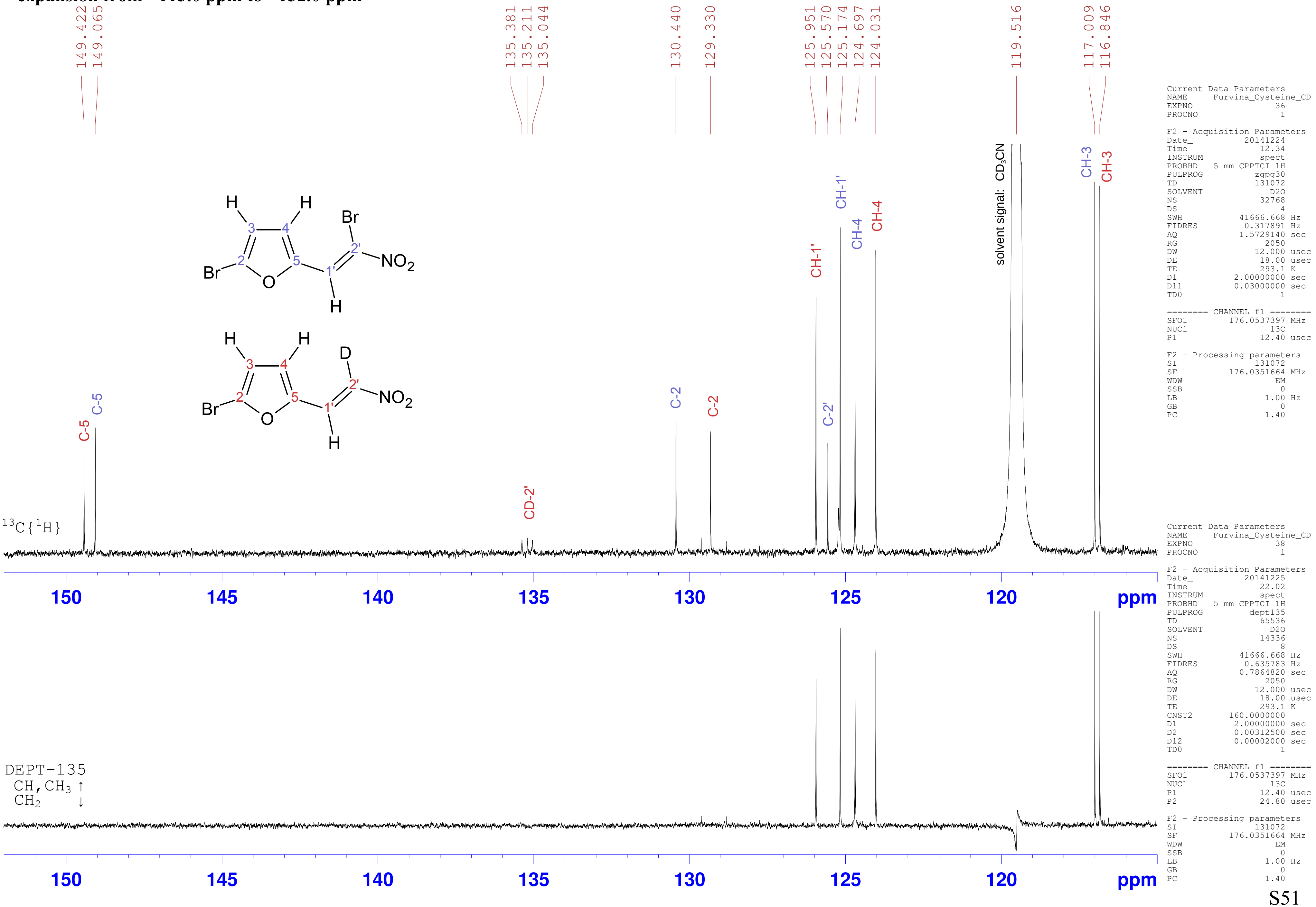

^{1}H-^{15}N HMBC NMR spectrum (700.1 MHz, 70.9 MHz) of G1 and 2-bromo-5-[2-nitro(2-^{2}H)ethenyl]furan solution in CD_3CN/D_2O (1:1)

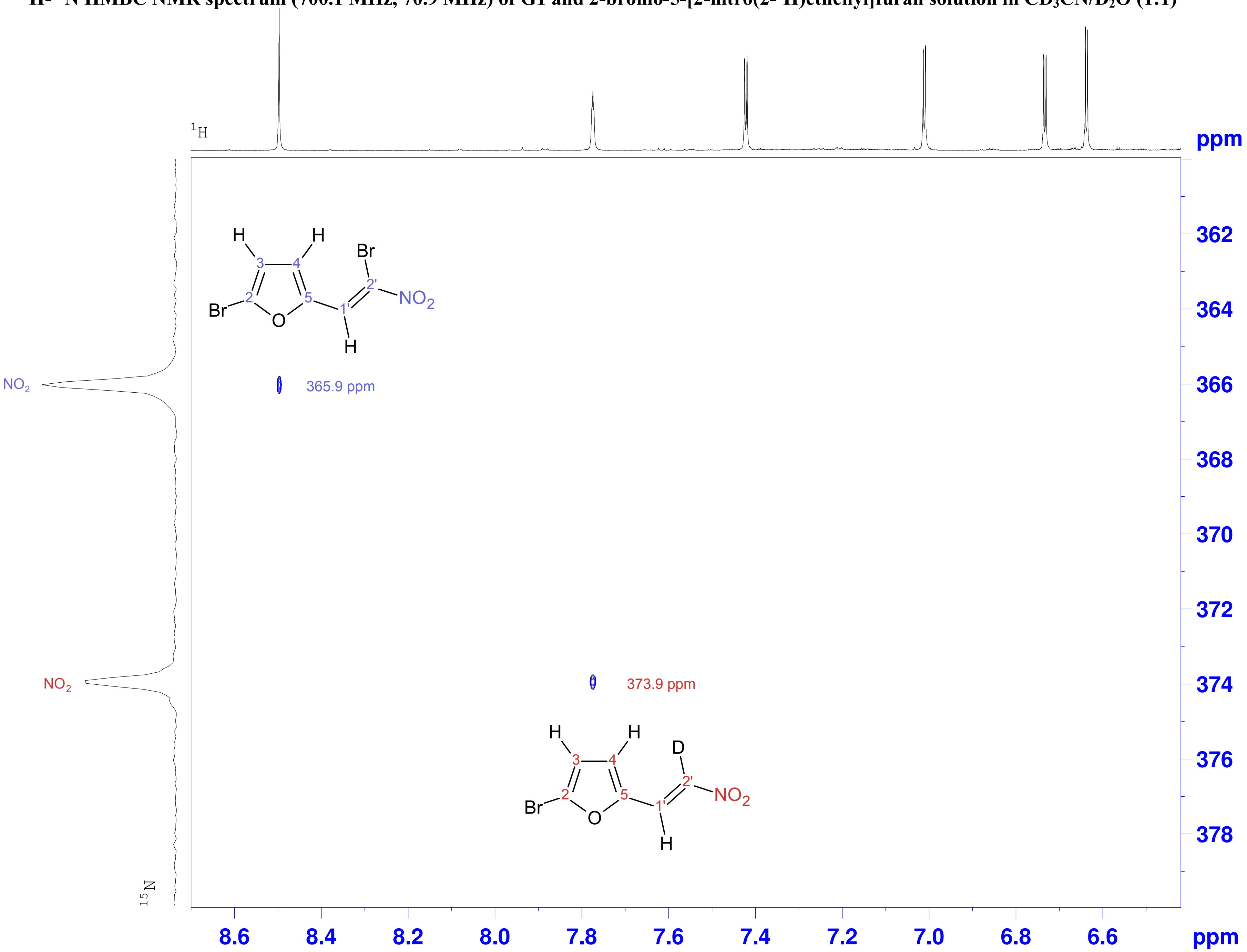


```
Current Data Parameters
NAME      Furvina_Cysteine_CD3CN-D2O
EXPNO                   41
PROCNO                   1

F2 - Acquisition Parameters
Date_             20141226
Time                 23.20
INSTRUM              spect
PROBHD   5 mm CPPTCI 1H
PULPROG    hmbcgplpndqf
TD                    2048
SOLVENT                D2O
NS                     400
DS                      16
SWH               1610.825 Hz
FIDRES            0.786536 Hz
AQ               0.6357492 sec
RG                    2050
DW                 310.400 usec
DE                   25.00 usec
TE                   293.1 K
CNST2           95.0000000
CNST13           7.0000000
D0              0.00000300 sec
D1              1.29999995 sec
D2              0.00526316 sec
D6              0.07142857 sec
D16             0.00020000 sec
IN0             0.00035240 sec

======== CHANNEL f1 ========
SFO1           700.0855656 MHz
NUC1                    1H
P1                    7.10 usec
P2                   14.20 usec

F1 - Acquisition parameters
ND0                      2
TD                      69
SFO1              70.96474 MHz
FIDRES           20.562931 Hz
SW                  19.994 ppm
FnMODE                  QF

F2 - Processing parameters
SI                    4096
SF             700.0802675 MHz
WDW                  QSINE
SSB                      2
LB                    0.00 Hz
GB                       0
PC                    1.40

F1 - Processing parameters
SI                     256
MC2                     QF
SF              70.9384928 MHz
WDW                  QSINE
SSB                      2
LB                    0.00 Hz
GB                       0
```

Spectra of diastereoisomeric 1:1 mixture of *S*-[(1*R*)-1-(5-bromofuran-2-yl)-2-nitro-ethyl]-*L*-cysteine and *S*-[(1*S*)-1-(5-bromofuran-2-yl)-2-nitroethyl]-*L*-cysteine (4a and 4b) solution in CD_3CN/H_2O

HRMS (m/z) calcd for $C_9H_{11}BrN_2NaO_5S$ $(M+Na)^+$ 360.9464, found 360.9458.

Diastereoisomer 4a:

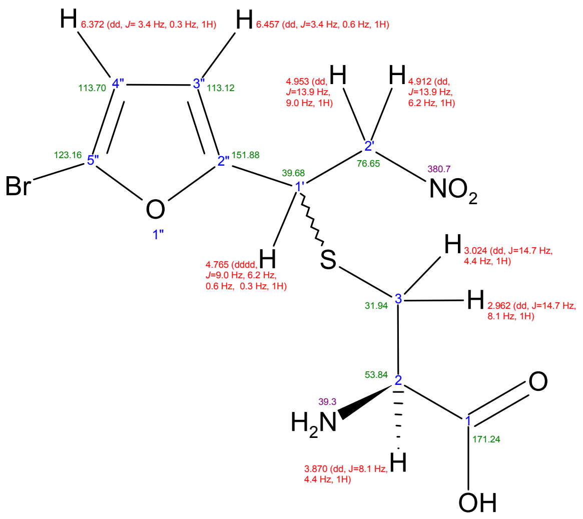

^{1}H NMR (700.1 MHz, CD_3CN/H_2O 1:1, 20.0 °C) δ: 6.457 (dd, $^3J_{HH}$ = 3.4 Hz, $^4J_{HH}$ = 0.6 Hz, 1H, C<u>*H*</u>-3”); 6.372 (dd, $^3J_{HH}$ = 3.4 Hz, $^5J_{HH}$ = 0.3 Hz, 1H, C<u>*H*</u>-4”); 4.953 (dd, $^2J_{HH}$ = 13.9 Hz, $^3J_{HH}$ = 9.0 Hz, 1H, C<u>*H*</u>-2’a); 4.912 (dd, $^2J_{HH}$ = 13.9 Hz, $^3J_{HH}$ = 6.2 Hz, 1H, C<u>*H*</u>-2’b); 4.765 (dddd, $^3J_{HH}$ = 9.0 Hz, $^3J_{HH}$ = 6.2 Hz, $^4J_{HH}$ = 0.6 Hz, $^5J_{HH}$ = 0.3 Hz, 1H, C<u>*H*</u>-1’); 3.870 (dd, $^3J_{HH}$ = 8.1 Hz, $^3J_{HH}$ = 4.4 Hz, 1H, C<u>*H*</u>-2); 3.024 (dd, $^2J_{HH}$ = 14.7 Hz, $^3J_{HH}$ = 4.4 Hz, 1H, C<u>*H*</u>-3a); 2.962 (dd, $^2J_{HH}$ = 14.7 Hz, $^3J_{HH}$ = 8.1 Hz, 1H, C<u>*H*</u>-3b). ^{13}C{^{1}H} NMR (176.0 MHz, CD_3CN/H_2O 1:1, 20.0 °C) δ: 171.24 (<u>*C*</u>-1); 151.88 (<u>*C*</u>-2”); 123.16 (<u>*C*</u>-5”); 113.70 (<u>*C*</u>H-4”); 113.12 (<u>*C*</u>H-3”); 76.65 (<u>*C*</u>H_2-2’); 53.84 (<u>*C*</u>H-2); 39.68 (<u>*C*</u>H-1’); 31.94 (<u>*C*</u>H_2-3). ^{15}N NMR (70.9 MHz, CD_3CN/H_2O 1:1, 20.0 °C) δ: 380.7 (<u>*N*</u>O_2); 39.3 (<u>*N*</u>H_2).

Diastereoisomer 4b:

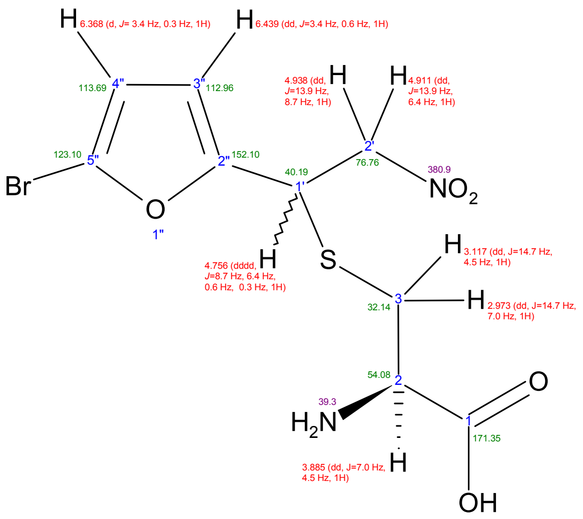

^{1}H NMR (700.1 MHz, CD_3CN/H_2O 1:1, 20.0 °C) δ: 6.439 (dd, $^{3}J_{HH}$ = 3.4 Hz, $^{4}J_{HH}$ = 0.6 Hz, 1H, C*H*-3”); 6.368 (dd, $^{3}J_{HH}$ = 3.4 Hz, $^{5}J_{HH}$ = 0.3 Hz, 1H, C*H*-4”); 4.938 (dd, $^{2}J_{HH}$ = 13.9 Hz, $^{3}J_{HH}$ = 8.7 Hz, 1H, C*H*-2’a); 4.911 (dd, $^{2}J_{HH}$ = 13.9 Hz, $^{3}J_{HH}$ = 6.4 Hz, 1H, C*H*-2’b); 4.756 (dddd, $^{3}J_{HH}$ = 8.7 Hz, $^{3}J_{HH}$ = 6.4 Hz, $^{4}J_{HH}$ = 0.6 Hz, $^{5}J_{HH}$ = 0.3 Hz, 1H, C*H*-1’); 3.885 (dd, $^{3}J_{HH}$ = 7.0 Hz, $^{3}J_{HH}$ = 4.5 Hz, 1H, C*H*-2); 3.117 (dd, $^{2}J_{HH}$ = 14.7 Hz, $^{3}J_{HH}$ = 4.5 Hz, 1H, C*H*-3a); 2.973 (dd, $^{2}J_{HH}$ = 14.7 Hz, $^{3}J_{HH}$ = 7.0 Hz, 1H, C*H*-3b). ^{13}C{^{1}H} NMR (176.0 MHz, CD_3CN/H_2O 1:1, 20.0 °C) δ: 171.35 (*C*-1); 152.10 (*C*-2”); 123.10 (*C*-5”); 113.69 (*C*H-4”); 112.96 (*C*H-3”); 76.76 (*C*H_2-2’); 54.08 (*C*H-2); 40.19 (*C*H-1’); 32.14 (*C*H_2-3). ^{15}N NMR (70.9 MHz, CD_3CN/H_2O 1:1, 20.0 °C) δ: 380.9 (*N*O_2); 39.3 (*N*H_2).

HRMS of *S*-[(1*R*)-1-(5-bromofuran-2-yl)-2-nitroethyl]-*L*-cysteine and *S*-[(1*S*)-1-(5-bromofuran-2-yl)-2-nitroethyl]-*L*-cysteine (4)

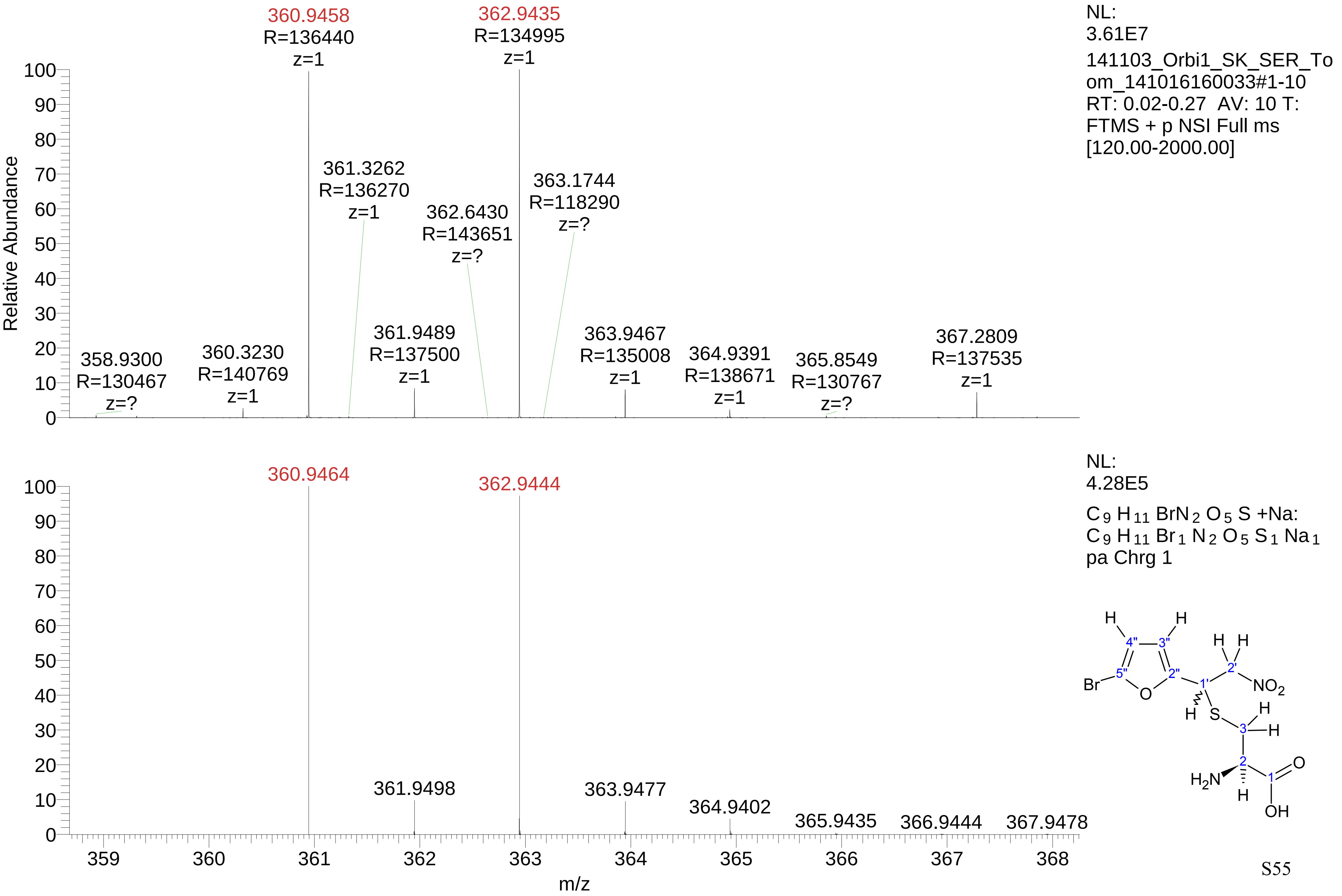

^{1}H NMR spectrum (700.1 MHz) of *S*-[(1*R*)-1-(5-bromofuran-2-yl)-2-nitroethyl]-*L*-cysteine and *S*-[(1*S*)-1-(5-bromofuran-2-yl)-2-nitroethyl]-*L*-cysteine (4) mixture in CD_3CN/H_2O (1:1)

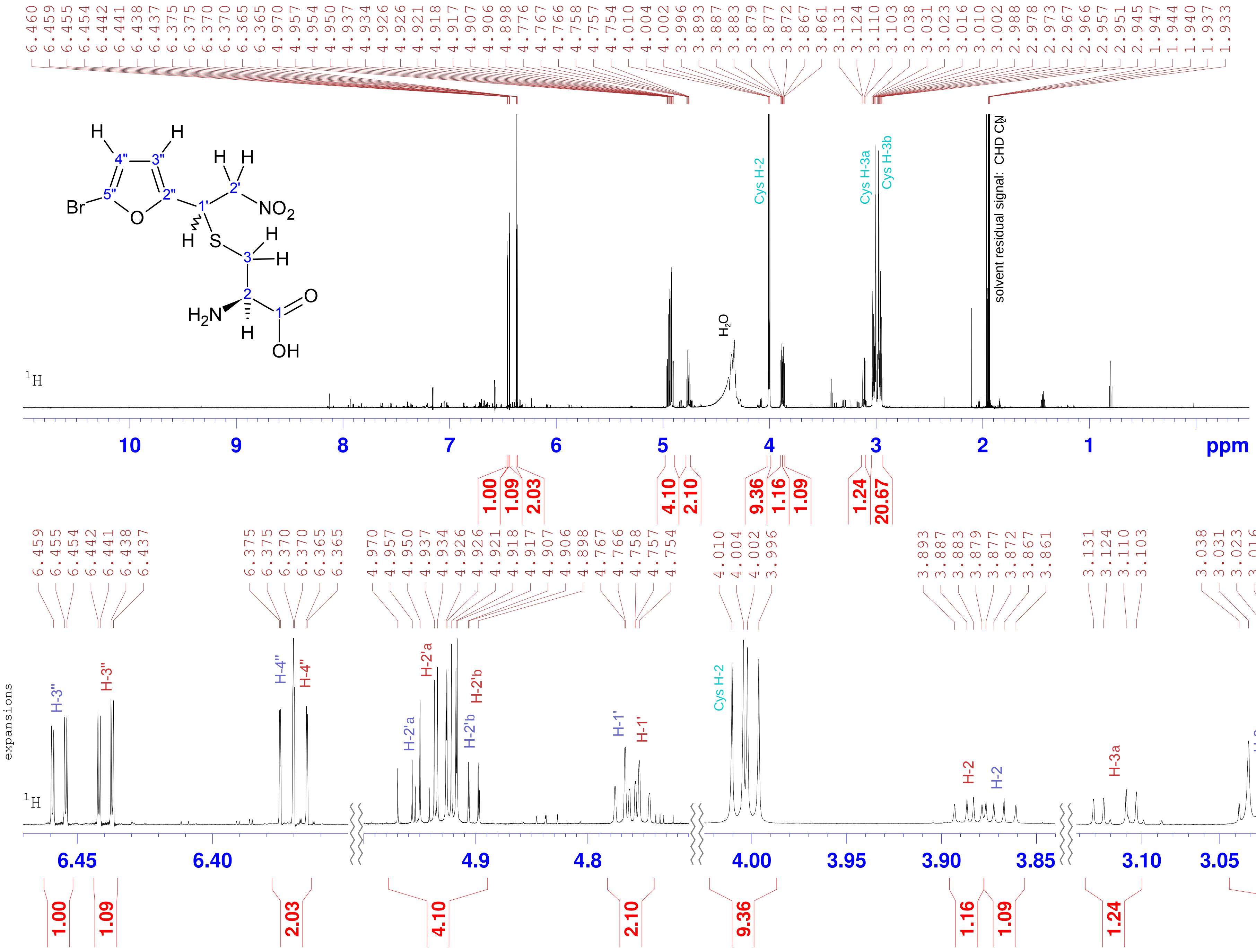

Current Data Parameters
NAME si
EXPNO 138
PROCNO 1

F2 - Acquisition Parameters
Date_ 20141125
Time 16.46
INSTRUM spect
PROBHD 5 mm CPPTCI 1H
PULPROG zgps
TD 131072
SOLVENT CD3CN
NS 16
DS 0
SWH 12626.263 Hz
FIDRES 0.096331 Hz
AQ 5.1905012 sec
RG 101
DW 39.600 usec
DE 25.00 usec
TE 293.1 K
D12 0.00002000 sec
L6 64
TD0 1

======== CHANNEL f1 ========
SFO1 700.0830432 MHz
NUC1 1H
P1 7.10 usec
P18 100000.00 usec

F2 - Processing parameters
SI 524288
SF 700.0800203 MHz
WDW GM
SSB 0
LB -0.20 Hz
GB 1
PC 1.00

$^{13}C\{^{1}H\}$ and DEPT-135 NMR spectra (176.0 MHz) of *S*-[(1*R*)-1-(5-bromofuran-2-yl)-2-nitroethyl]-*L*-cysteine and *S*-[(1*S*)-1-(5-bromofuran-2-yl)-2-nitroethyl]-*L*-cysteine (4) mixture in CD_3CN/H_2O (1:1)

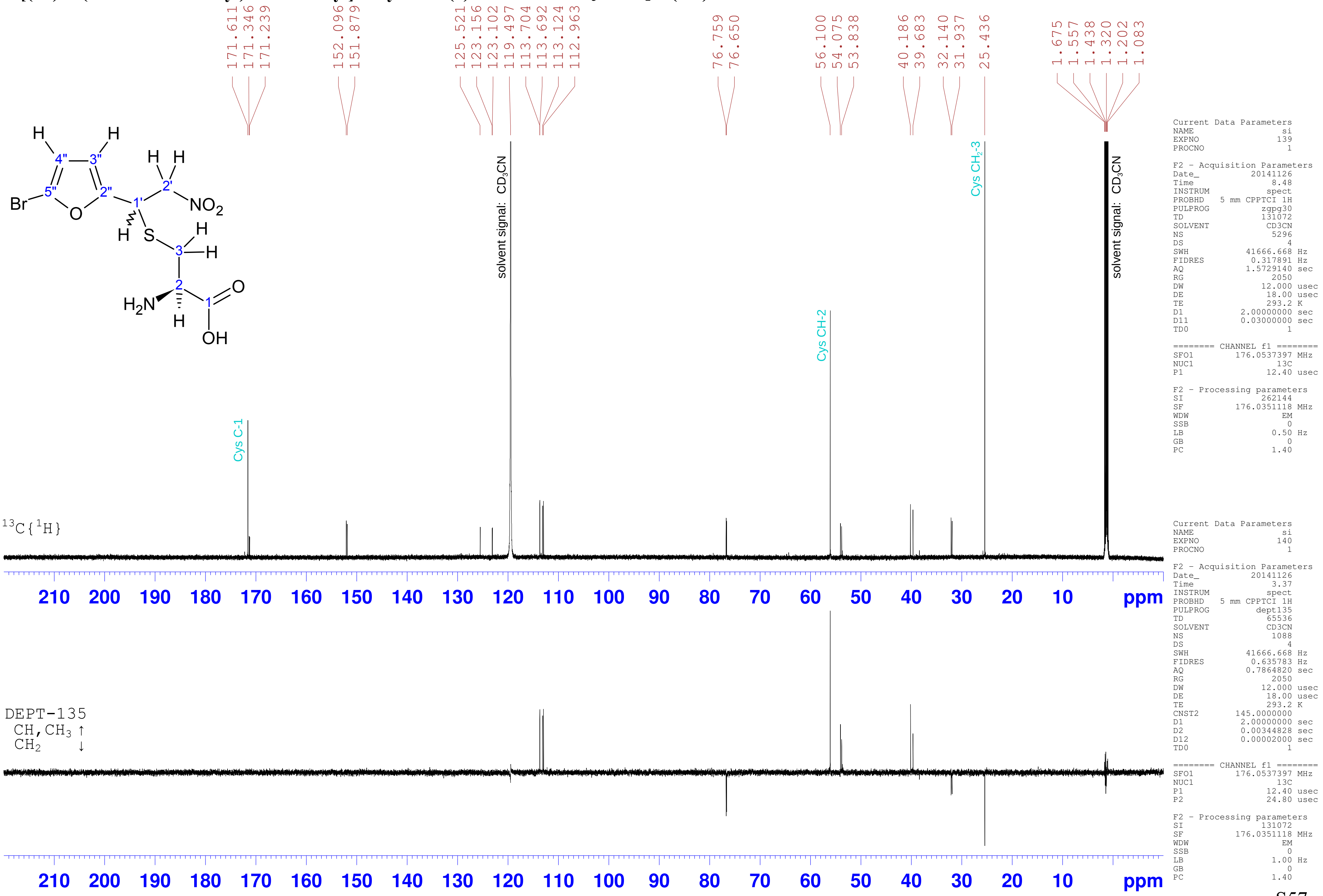

$^{13}C\{^1H\}$ and DEPT-135 NMR spectra (176.0 MHz) of *S*-[(1*R*)-1-(5-bromofuran-2-yl)-2-nitroethyl]-*L*-cysteine and *S*-[(1*S*)-1-(5-bromofuran-2-yl)-2-nitroethyl]-*L*-cysteine (4) mixture in CD_3CN/H_2O (1:1) – expansion from +110.0 ppm to +175.0 ppm

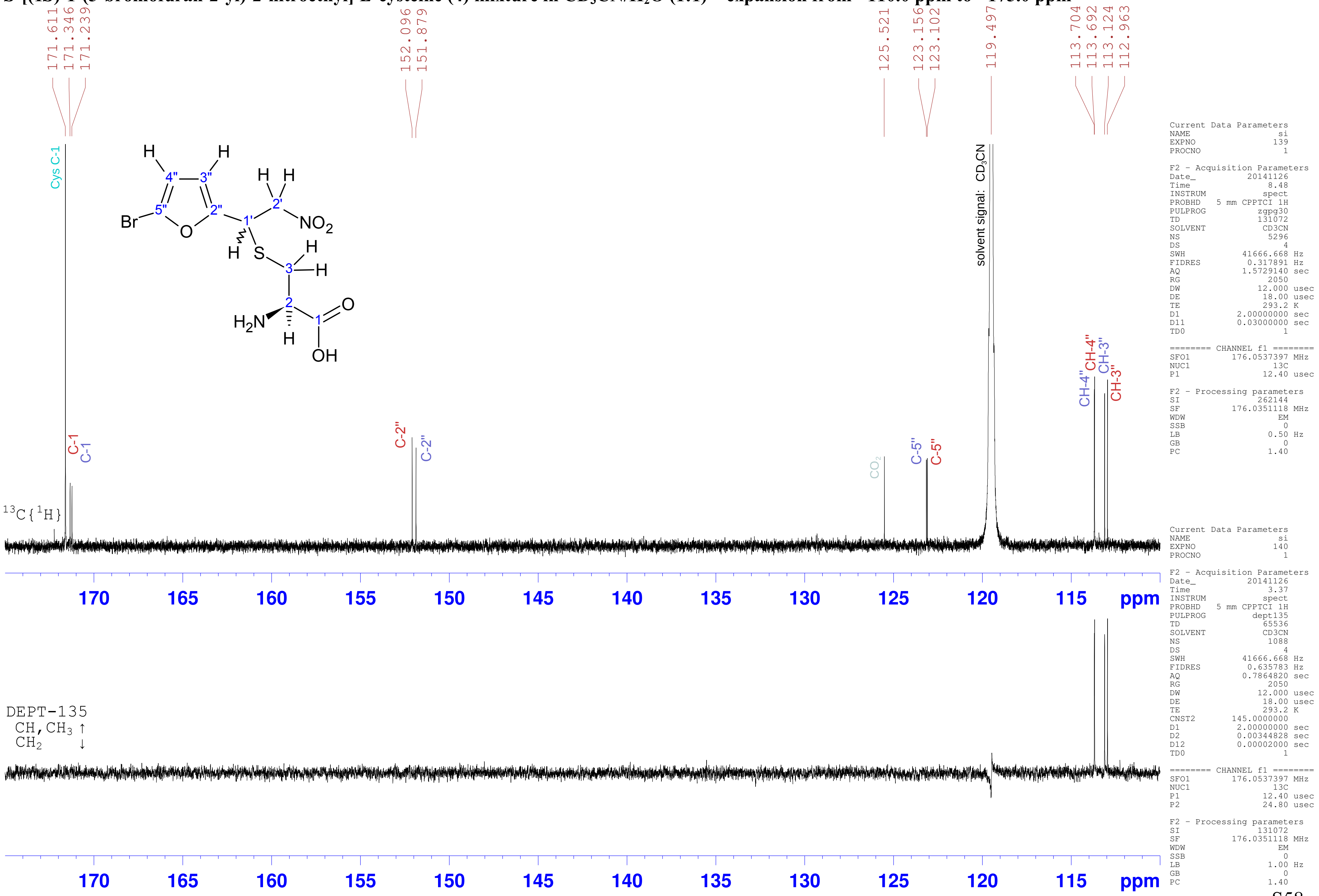

$^{13}C\{^{1}H\}$ and DEPT-135 NMR spectra (176.0 MHz) of *S*-[(1*R*)-1-(5-bromofuran-2-yl)-2-nitroethyl]-*L*-cysteine and *S*-[(1*S*)-1-(5-bromofuran-2-yl)-2-nitroethyl]-*L*-cysteine (4) mixture in CD_3CN/H_2O (1:1) – expansion from +22.0 ppm to +80.0 ppm

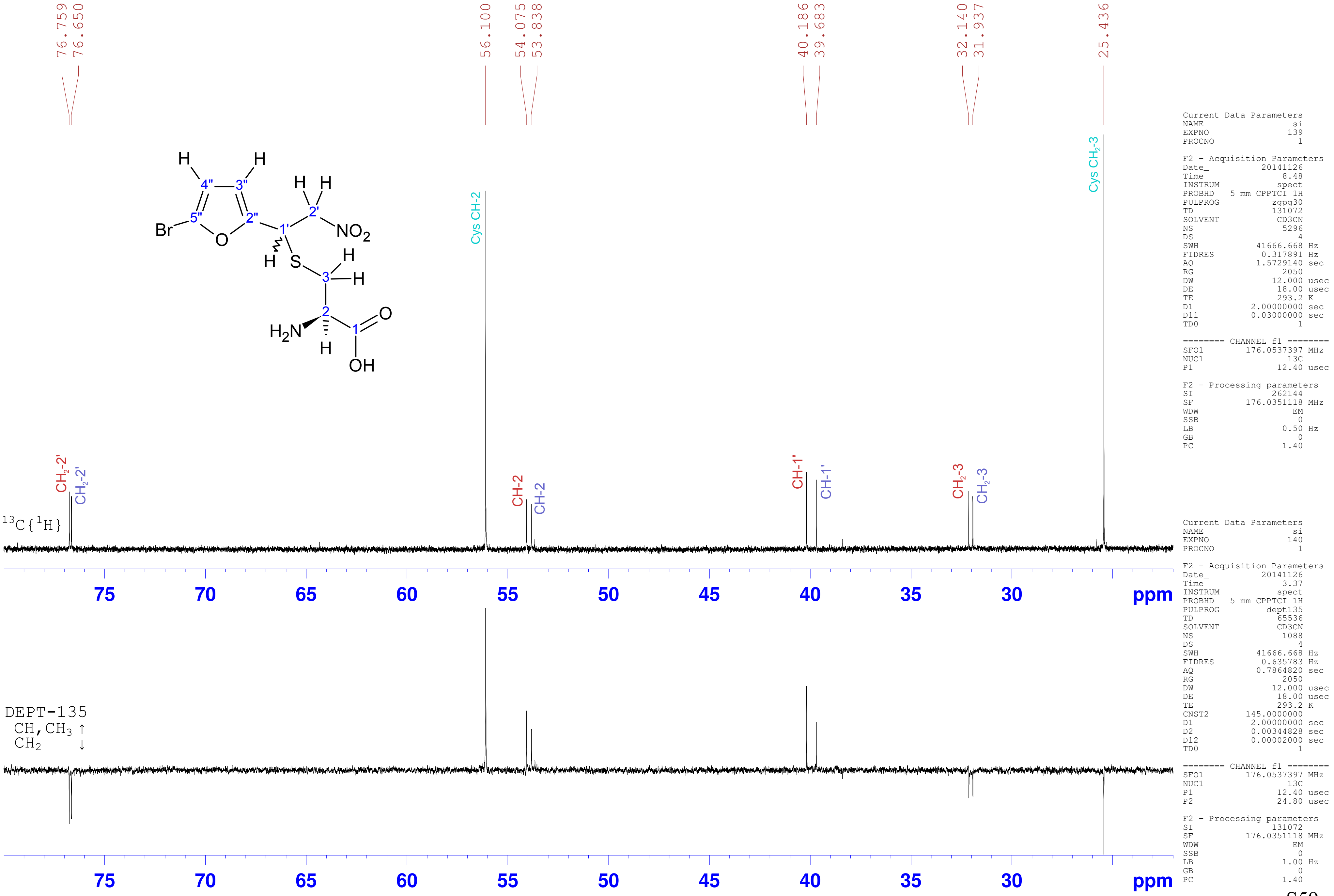

^{1}H-^{15}N HMBC NMR spectrum (700.1 MHz, 70.9 MHz) of *S*-[(1*R*)-1-(5-bromofuran-2-yl)-2-nitroethyl]-*L*-cysteine and *S*-[(1*S*)-1-(5-bromofuran-2-yl)-2-nitroethyl]-*L*-cysteine (4) mixture in CD_3CN/H_2O (1:1)

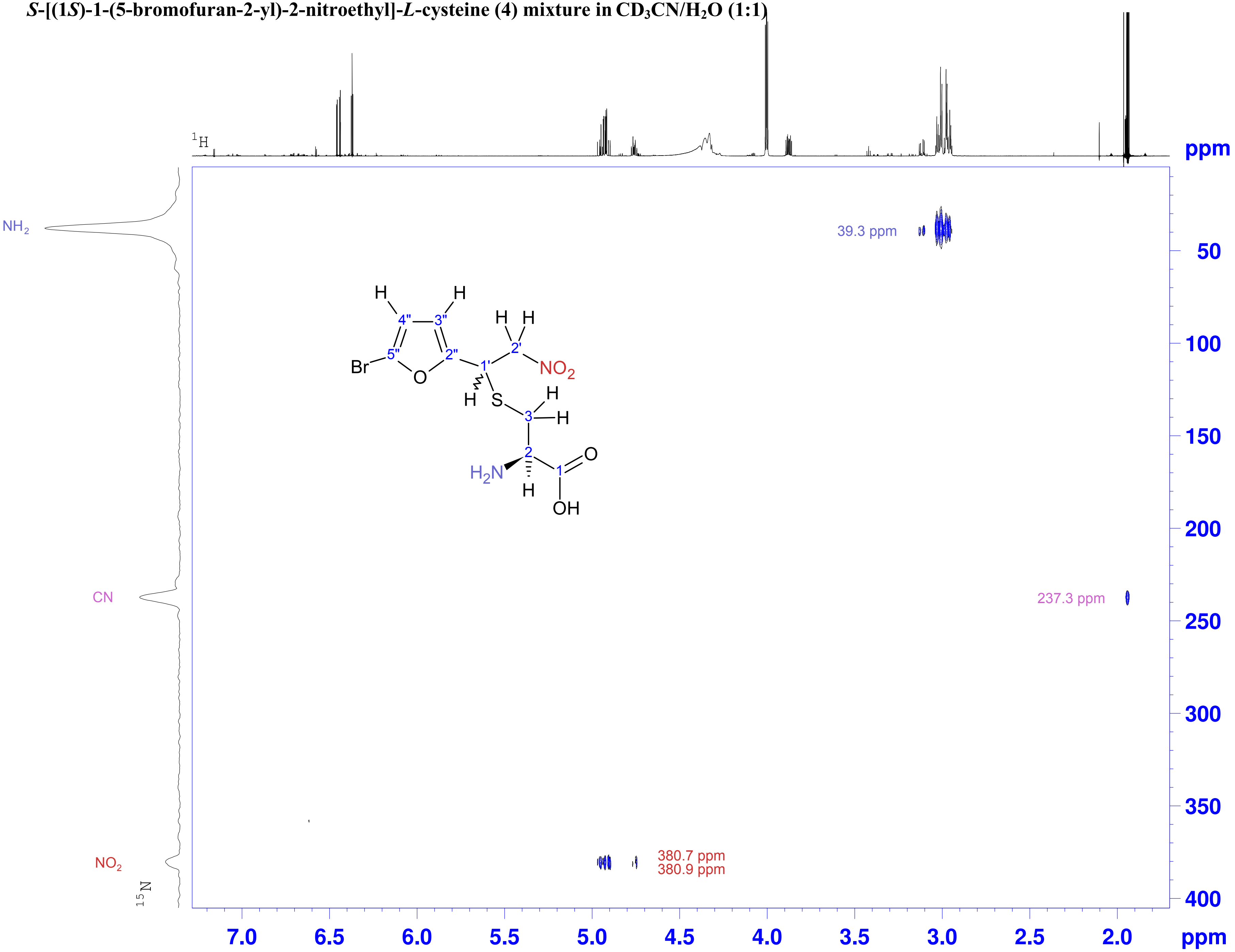


```
Current Data Parameters
NAME                    si
EXPNO                  143
PROCNO                   1

F2 - Acquisition Parameters
Date_             20141125
Time                 21.45
INSTRUM              spect
PROBHD   5 mm CPPTCI 1H
PULPROG  hmbcgplpndprqf
TD                    4096
SOLVENT              CD3CN
NS                     160
DS                      16
SWH              5175.983 Hz
FIDRES           1.263668 Hz
AQ              0.3957236 sec
RG                    1030
DW                  96.600 usec
DE                   25.00 usec
TE                   293.1 K
CNST2           95.0000000
CNST13           7.0000000
D0              0.00000300 sec
D1              1.29999995 sec
D2              0.00526316 sec
D6              0.07142857 sec
D11             0.03000000 sec
D12             0.00002000 sec
D16             0.00020000 sec
IN0             0.00001760 sec

======== CHANNEL f1 ========
SFO1           700.0830432 MHz
NUC1                    1H
P1                    7.10 usec
P2                   14.20 usec

F1 - Acquisition parameters
ND0                      2
TD                      64
SFO1            70.95303 MHz
FIDRES          443.892059 Hz
SW                 400.393 ppm
FnMODE                  QF

F2 - Processing parameters
SI                    4096
SF             700.0800203 MHz
WDW                  QSINE
SSB                      2
LB                    0.00 Hz
GB                       0
PC                    1.40

F1 - Processing parameters
SI                    1024
MC2                     QF
SF              70.9384928 MHz
WDW                  QSINE
SSB                      2
LB                    0.00 Hz
GB                       0
```

Spectra of diastereoisomeric 1:1 mixture of *S*-[(1*R*)-1-(5-bromofuran-2-yl)-2-nitro(2,2-$^2H_2$)ethyl]-*L*-(*N*,*N*,*O*-$^2H_3$)cysteine and *S*-[(1*S*)-1-(5-bromofuran-2-yl)-2-nitro(2,2-$^2H_2$)ethyl]-*L*-(*N*,*N*,*O*-$^2H_3$)cysteine solution in CD_3CN/D_2O

Diastereoisomer deuterated-4a:

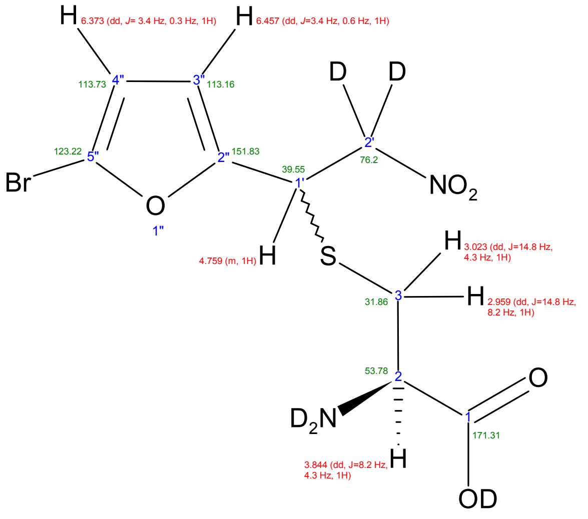

^{1}H NMR (700.1 MHz, CD_3CN/D_2O 1:1, 20.0 °C) δ: 6.457 (dd, $^3J_{HH}$ = 3.4 Hz, $^4J_{HH}$ = 0.6 Hz, 1H, C<u>*H*</u>-3”); 6.373 (dd, $^3J_{HH}$ = 3.4 Hz, $^5J_{HH}$ = 0.3 Hz, 1H, C<u>*H*</u>-4”); 4.759 (m, 1H, C<u>*H*</u>-1’); 3.844 (dd, $^3J_{HH}$ = 8.2 Hz, $^3J_{HH}$ = 4.3 Hz, 1H, C<u>*H*</u>-2); 3.023 (dd, $^2J_{HH}$ = 14.8 Hz, $^3J_{HH}$ = 4.3 Hz, 1H, C<u>*H*</u>-3a); 2.959 (dd, $^2J_{HH}$ = 14.8 Hz, $^3J_{HH}$ = 8.2 Hz, 1H, C<u>*H*</u>-3b). $^{13}C\{^1H\}$ NMR (176.0 MHz, CD_3CN/D_2O 1:1, 20.0 °C) δ: 171.31 (<u>*C*</u>-1); 151.83 (<u>*C*</u>-2”); 123.22 (<u>*C*</u>-5”); 113.73 (<u>*C*</u>H-4”); 113.16 (<u>*C*</u>H-3”); 76.2 (m, <u>*C*</u>H_2-2’); 53.78 (<u>*C*</u>H-2); 39.55 (<u>*C*</u>H-1’); 31.86 (<u>*C*</u>H_2-3).

Diastereoisomer deuterated-4b:

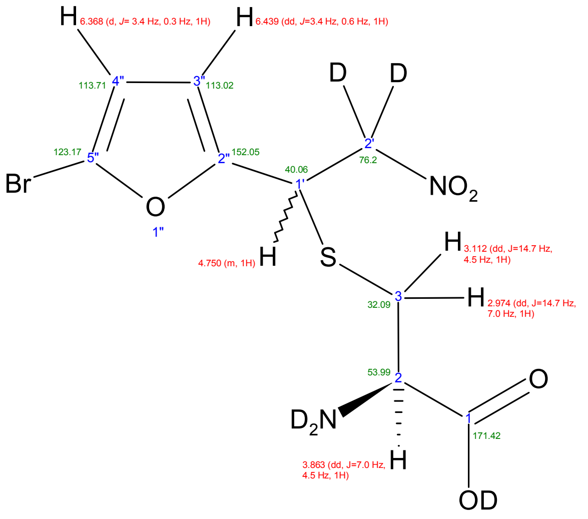

^{1}H NMR (700.1 MHz, CD_3CN/D_2O 1:1, 20.0 °C) δ: 6.439 (dd, $^{3}J_{HH}$ = 3.4 Hz, $^{4}J_{HH}$ = 0.6 Hz, 1H, C*H*-3"); 6.368 (dd, $^{3}J_{HH}$ = 3.4 Hz, $^{5}J_{HH}$ = 0.3 Hz, 1H, C*H*-4"); 4.750 (m, 1H, C*H*-1'); 3.863 (dd, $^{3}J_{HH}$ = 7.0 Hz, $^{3}J_{HH}$ = 4.5 Hz, 1H, C*H*-2); 3.112 (dd, $^{2}J_{HH}$ = 14.7 Hz, $^{3}J_{HH}$ = 4.5 Hz, 1H, C*H*-3a); 2.974 (dd, $^{2}J_{HH}$ = 14.7 Hz, $^{3}J_{HH}$ = 7.0 Hz, 1H, C*H*-3b). $^{13}C\{^{1}H\}$ NMR (176.0 MHz, CD_3CN/D_2O 1:1, 20.0 °C) δ: 171.42 (*C*-1); 152.05 (*C*-2"); 123.17 (*C*-5"); 113.71 (*C*H-4"); 113.02 (*C*H-3"); 76.2 (m, *C*H_2-2'); 53.99 (*C*H-2); 40.06 (*C*H-1'); 32.09 (*C*H_2-3).

^{1}H NMR spectrum (700.1 MHz) of *S*-[(1*R*)-1-(5-bromofuran-2-yl)-2-nitro(2,2-^{2}H2)ethyl]-*L*-(*N*,*N*,*O*-^{2}H3)cysteine and *S*-[(1*S*)-1-(5-bromofuran-2-yl)-2-nitro(2,2-^{2}H2)ethyl]-*L*-(*N*,*N*,*O*-^{2}H3)cysteine solution in CD_3CN/D_2O (1:1)

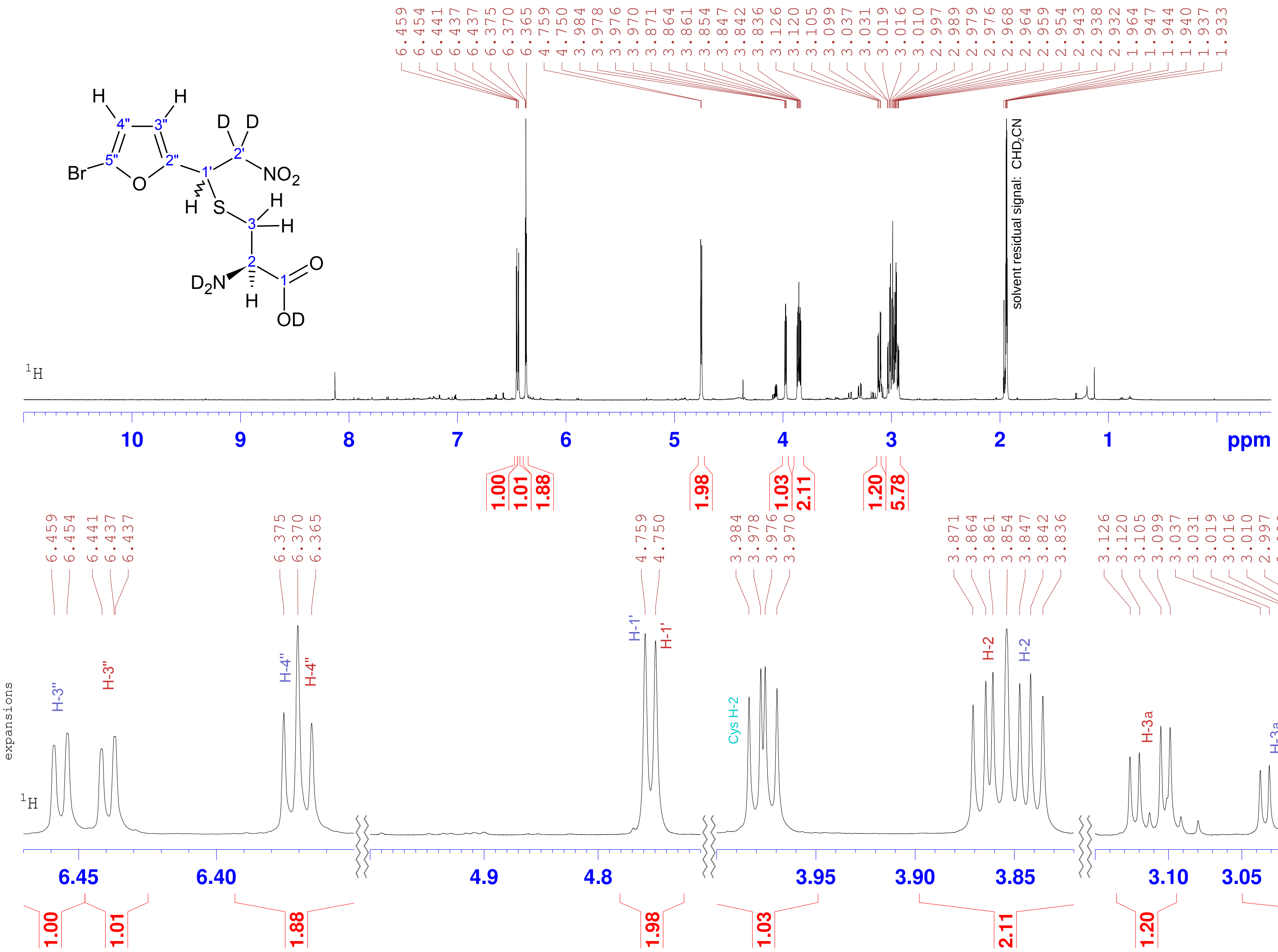


```
Current Data Parameters
NAME       Furvina_Cystein
EXPNO                   89
PROCNO                   1

F2 - Acquisition Parameters
Date_             20150105
Time                  9.56
INSTRUM              spect
PROBHD    5 mm CPPTCI 1H
PULPROG               zgps
TD                   65536
SOLVENT                D2O
NS                       8
DS                       4
SWH           12626.263 Hz
FIDRES         0.192661 Hz
AQ            2.5952756 sec
RG                     128
DW               39.600 usec
DE                25.00 usec
TE                293.1 K
D12          0.00002000 sec
L6                      64
TD0                      1

======== CHANNEL f1 ========
SFO1        700.0832871 MHz
NUC1                    1H
P1                7.10 usec
P18          100000.00 usec

F2 - Processing parameters
SI                  262144
SF          700.0802310 MHz
WDW                     EM
SSB                      0
LB                 0.10 Hz
GB                       0
PC                    1.00
```

$^{13}C\{^1H\}$ and DEPT-135 NMR spectra (176.0 MHz) of *S*-[(1*R*)-1-(5-bromofuran-2-yl)-2-nitro(2,2-$^2H_2$)ethyl]-*L*-(*N*,*N*,*O*-$^2H_3$)cysteine and *S*-[(1*S*)-1-(5-bromofuran-2-yl)-2-nitro(2,2-$^2H_2$)ethyl]-*L*- (*N*,*N*,*O*-$^2H_3$)cysteine solution in CD_3CN/D_2O (1:1)

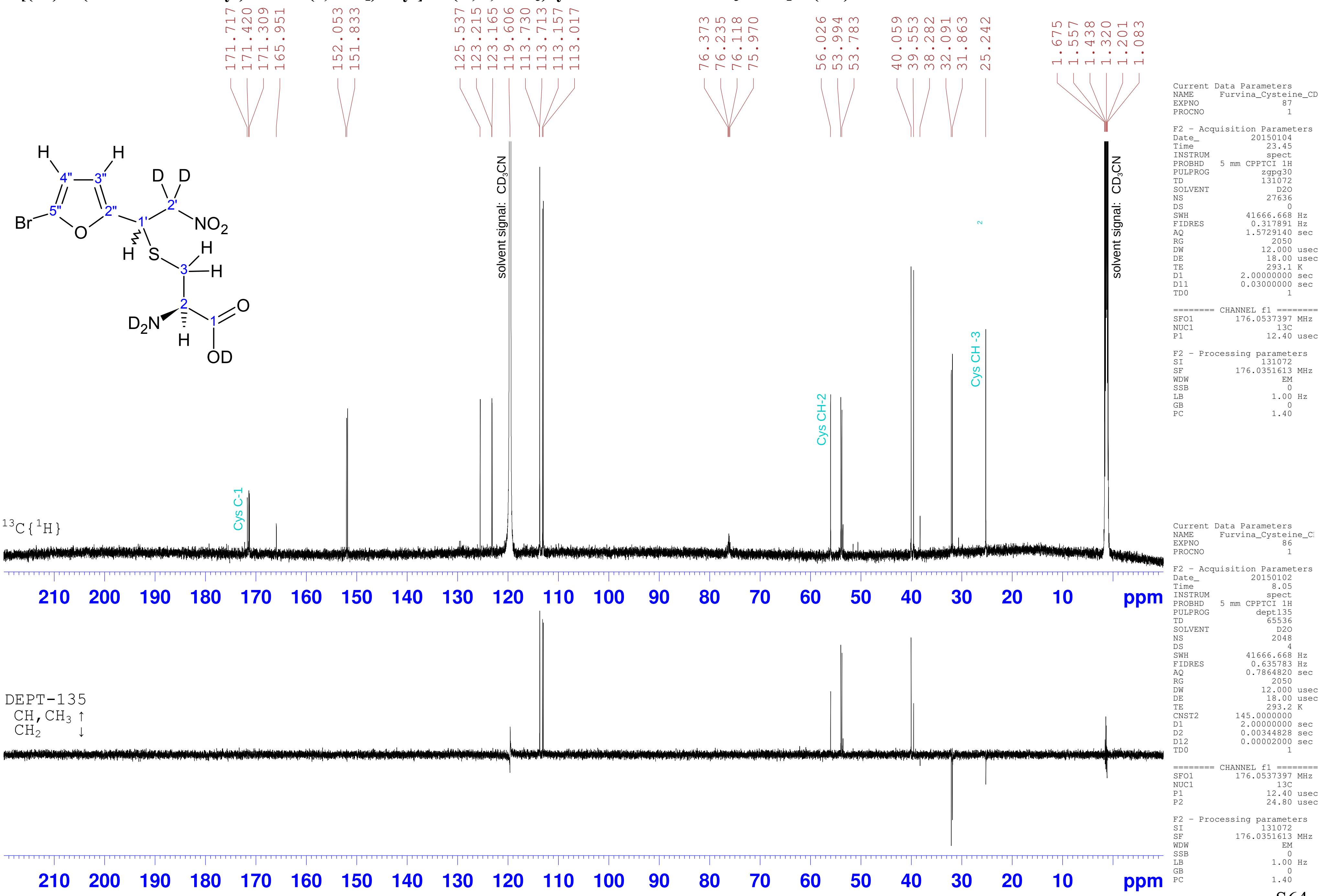

$^{13}C\{^1H\}$ and DEPT-135 NMR spectra (176.0 MHz) of *S*-[(1*R*)-1-(5-bromofuran-2-yl)-2-nitro(2,2-$^2H_2$)ethyl]-*L*-(*N*,*N*,*O*-$^2H_3$)cysteine and *S*-[(1*S*)-1-(5-bromofuran-2-yl)-2-nitro(2,2-$^2H_2$)ethyl]-*L*- (*N*,*N*,*O*-$^2H_3$)cysteine solution in CD_3CN/D_2O (1:1) – expansion from +110.0 ppm to +175.0 ppm

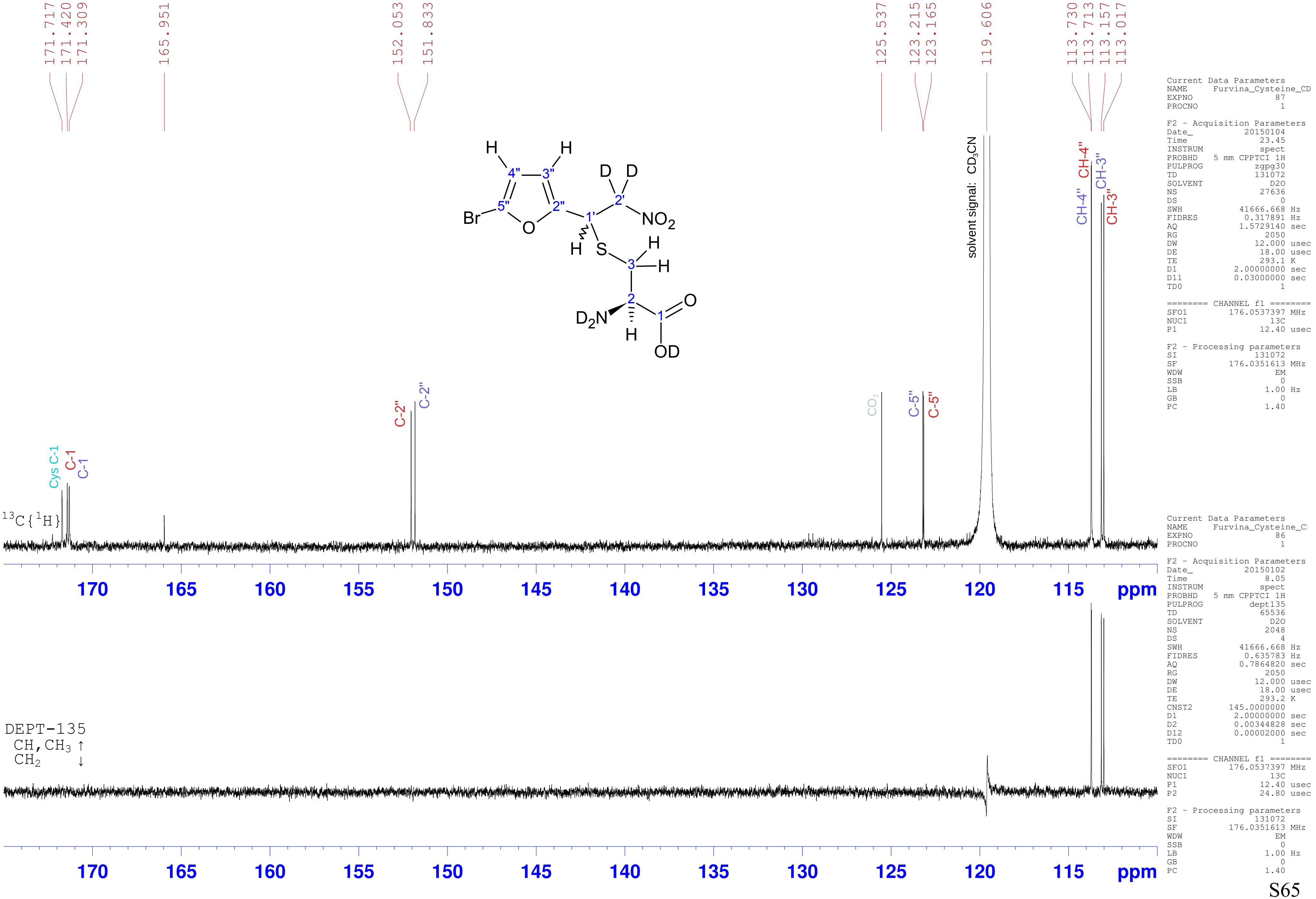

$^{13}C\{^1H\}$ and DEPT-135 NMR spectra (176.0 MHz) of *S*-[(1*R*)-1-(5-bromofuran-2-yl)-2-nitro(2,2-$^2H_2$)ethyl]-*L*-(*N*,*N*,*O*-$^2H_3$)cysteine and *S*-[(1*S*)-1-(5-bromofuran-2-yl)-2-nitro(2,2-$^2H_2$)ethyl]-*L*- (*N*,*N*,*O*-$^2H_3$)cysteine solution in CD_3CN/D_2O (1:1) – expansion from +22.0 ppm to +80.0 ppm

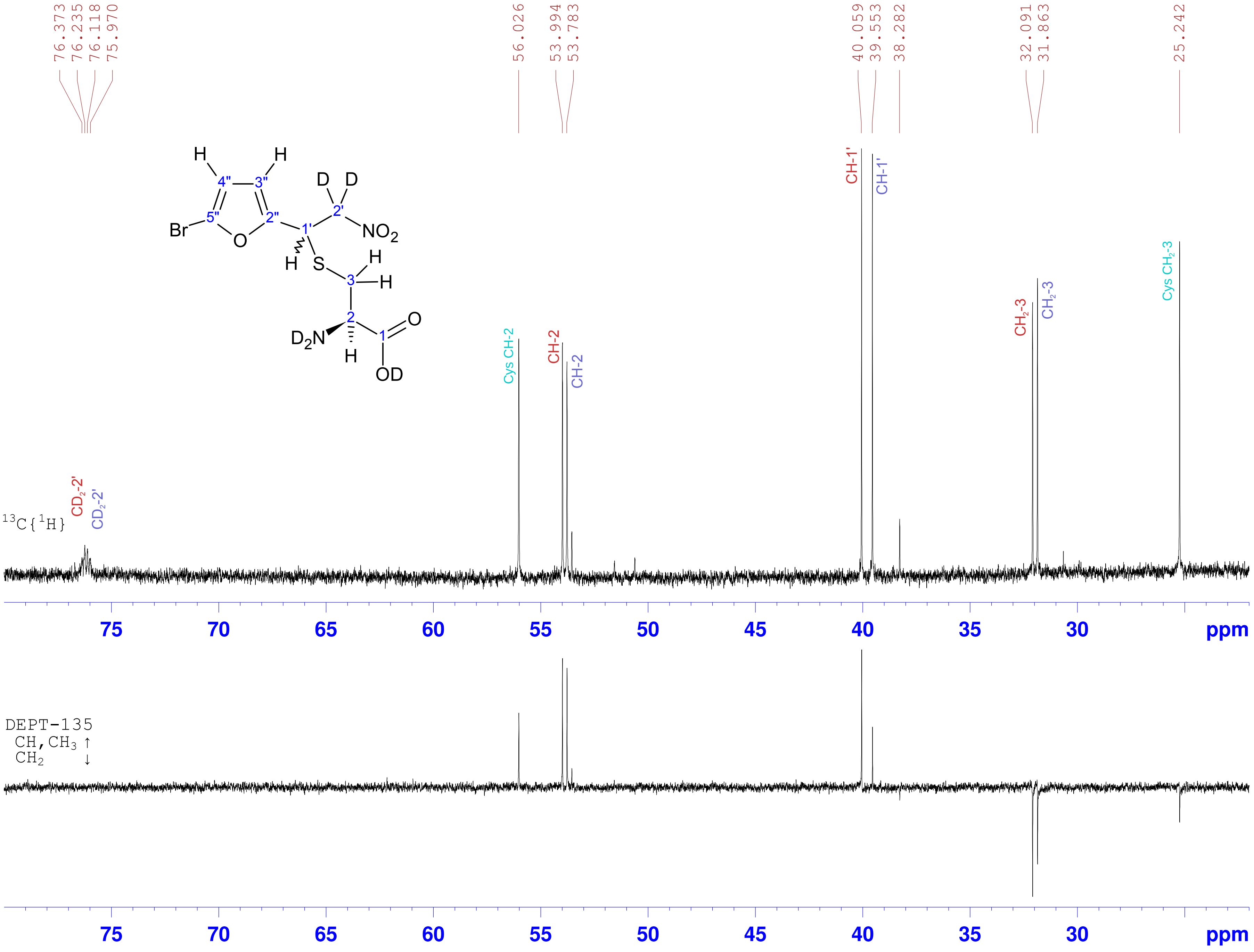

Current Data Parameters
NAME Furvina_Cysteine_CD
EXPNO 87
PROCNO 1

F2 - Acquisition Parameters
Date_ 20150104
Time 23.45
INSTRUM spect
PROBHD 5 mm CPPTCI 1H
PULPROG zgpg30
TD 131072
SOLVENT D2O
NS 27636
DS 0
SWH 41666.668 Hz
FIDRES 0.317891 Hz
AQ 1.5729140 sec
RG 2050
DW 12.000 usec
DE 18.00 usec
TE 293.1 K
D1 2.00000000 sec
D11 0.03000000 sec
TD0 1

======== CHANNEL f1 ========
SFO1 176.0537397 MHz
NUC1 13C
P1 12.40 usec

F2 - Processing parameters
SI 131072
SF 176.0351613 MHz
WDW EM
SSB 0
LB 1.00 Hz
GB 0
PC 1.40

Current Data Parameters
NAME Furvina_Cysteine_C
EXPNO 86
PROCNO 1

F2 - Acquisition Parameters
Date_ 20150102
Time 8.05
INSTRUM spect
PROBHD 5 mm CPPTCI 1H
PULPROG dept135
TD 65536
SOLVENT D2O
NS 2048
DS 4
SWH 41666.668 Hz
FIDRES 0.635783 Hz
AQ 0.7864820 sec
RG 2050
DW 12.000 usec
DE 18.00 usec
TE 293.2 K
CNST2 145.0000000
D1 2.00000000 sec
D2 0.00344828 sec
D12 0.00002000 sec
TD0 1

======== CHANNEL f1 ========
SFO1 176.0537397 MHz
NUC1 13C
P1 12.40 usec
P2 24.80 usec

F2 - Processing parameters
SI 131072
SF 176.0351613 MHz
WDW EM
SSB 0
LB 1.00 Hz
GB 0
PC 1.40

Spectra of 2-bromo-5-[1-(decylsulfanyl)-2-nitroethyl]furan (5) solution in $CDCl_3$

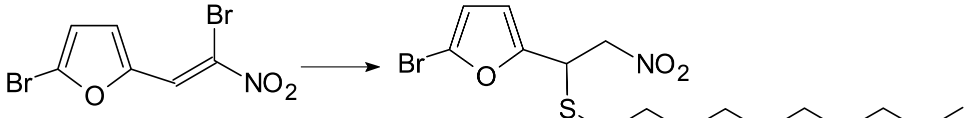

G1 **5**

G1 (6.5 mg, 21.9 μmol) was dissolved in CH_3OH (0.3 mL), followed by addition of 1-decanethiol (18 mg, 0.10 mmol) and saturated aqueous solution of $NaHCO_3$ (80 μL). After mixing, the solvent was evaporated under vacuum and the product was dissolved in $CDCl_3$ for NMR studies.

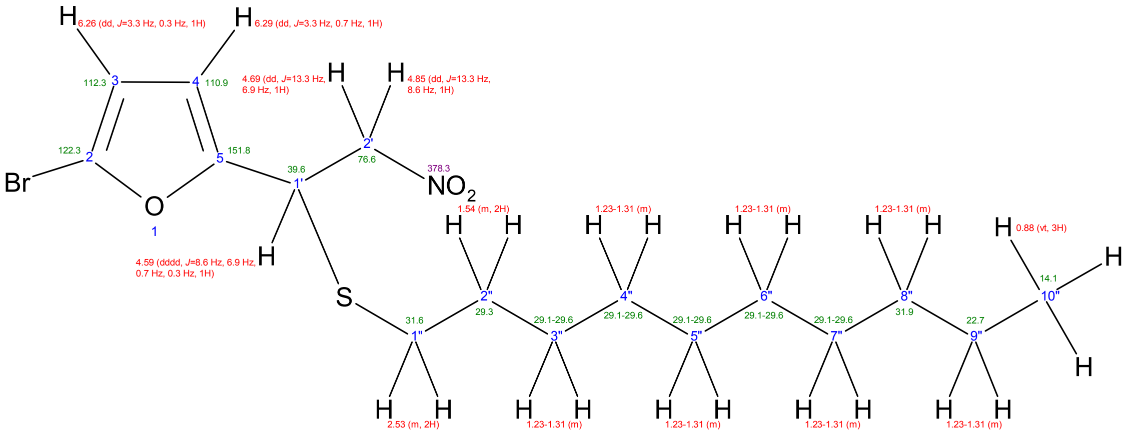

^{1}H NMR (700.1 MHz, $CDCl_3$, 20.0 °C) δ: 6.29 (dd, $^{3}J_{HH}$ = 3.3 Hz, $^{4}J_{HH}$ = 0.7 Hz, 1H, C$\underline{H}$-4); 6.26 (dd, $^{3}J_{HH}$ = 3.3 Hz, $^{5}J_{HH}$ = 0.3 Hz, 1H, C$\underline{H}$-3); 4.85 (dd, $^{2}J_{HH}$ = 13.3 Hz, $^{3}J_{HH}$ = 8.6 Hz, 1H, C$\underline{H}$-2'a); 4.69 (dd, $^{2}J_{HH}$ = 13.3 Hz, $^{3}J_{HH}$ = 6.9 Hz, 1H, C$\underline{H}$-2'b); 4.59 (dddd, $^{3}J_{HH}$ = 8.6 Hz, $^{3}J_{HH}$ = 6.9 Hz, $^{4}J_{HH}$ = 0.7 Hz, $^{5}J_{HH}$ = 0.3 Hz, 1H, C$\underline{H}$-1'); 2.53 (m, 2H, C$\underline{H}_2$-1"); 1.54 (m, 2H, C$\underline{H}_2$-2"); 1.23–1.31 (m, 14H, C$\underline{H}_2$-3"–C$\underline{H}_2$-9"); 0.88 (vt, 3H, C$\underline{H}_3$). ^{13}C NMR (176.0 MHz, $CDCl_3$, 20.0 °C) δ: 151.8 ($\underline{C}$-5); 122.3 ($\underline{C}$-2); 112.3 ($\underline{C}$H-3); 110.9 ($\underline{C}$H-4); 76.5 ($\underline{C}H_2$-2'); 39.6 ($\underline{C}$H-1'); 31.9 ($\underline{C}$H-8"); 31.6 ($\underline{C}$H-1"); 29.1–29.6 (C$\underline{H}_2$-2"–C$\underline{H}_2$-7"); 22.7 (C$\underline{H}_2$-9"); 14.1 (C$\underline{H}_2$-10"). ^{15}N NMR (70.9 MHz, $CDCl_3$, 20.0 °C) δ: 378.3 ($\underline{N}O_2$).

^{1}H NMR spectrum (700.1 MHz) of 2-bromo-5-[1-(decylsulfanyl)-2-nitroethyl]furan (5) solution in $CDCl_3$

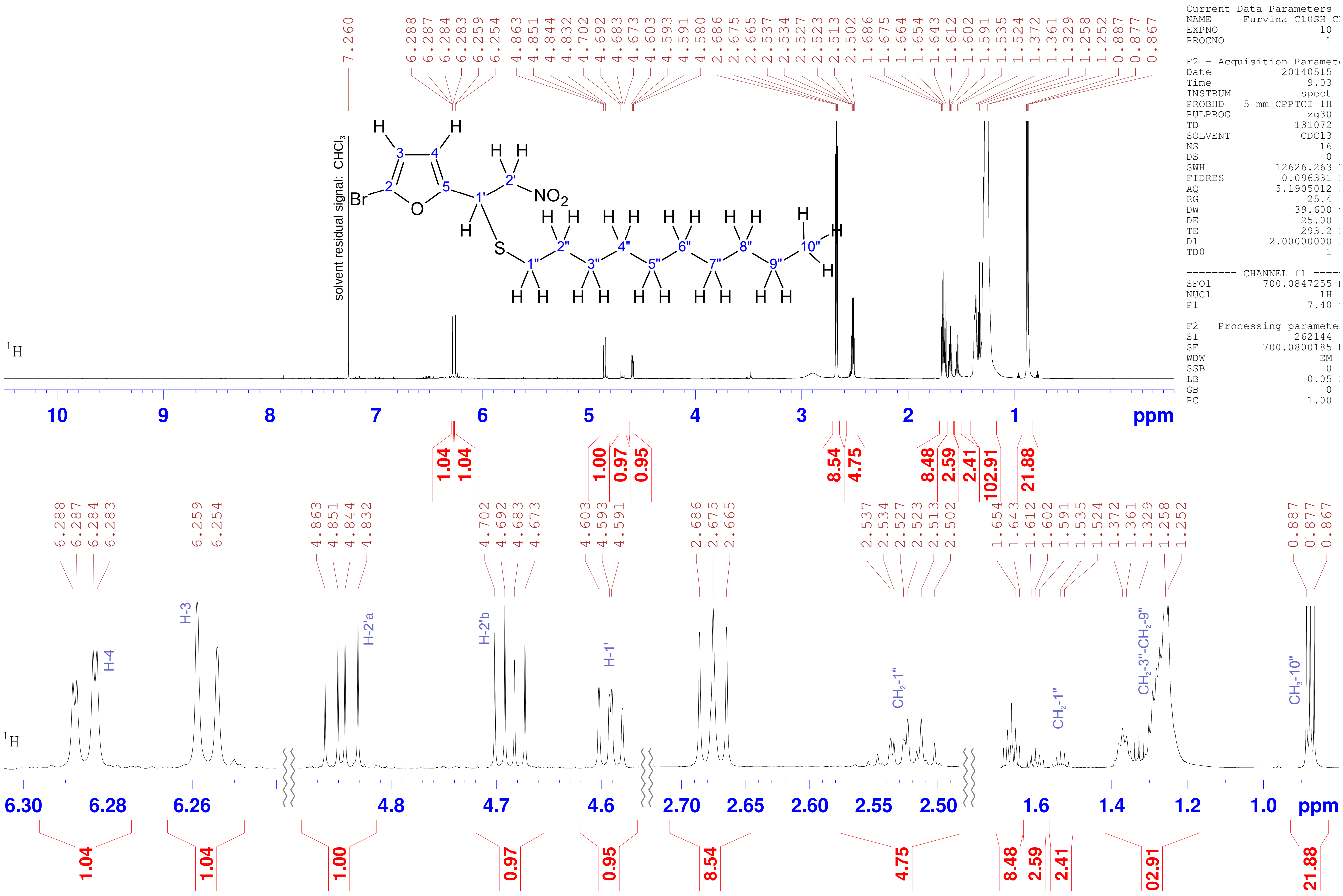

$^{13}C\{^{1}H\}$ and DEPT-135 NMR spectra (176.0 MHz) of 2-bromo-5-[1-(decylsulfanyl)-2-nitroethyl]furan (5) solution in $CDCl_3$

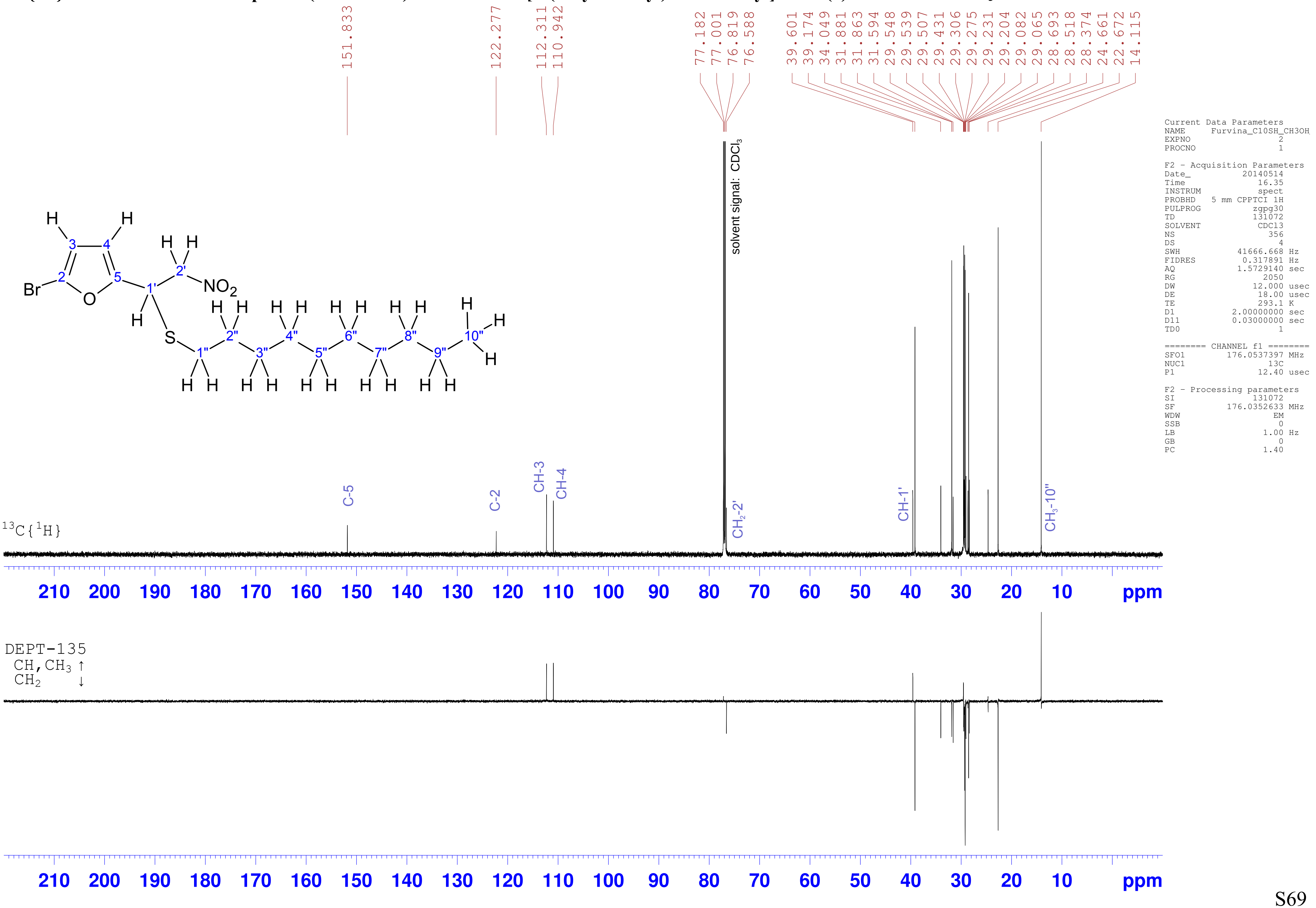

$^{13}C\{^1H\}$ and DEPT-135 NMR spectra (176.0 MHz) of 2-bromo-5-[1-(decylsulfanyl)-2-nitroethyl]furan (5) solution in $CDCl_3$ – expansion from +105.0 ppm to +155.0 ppm

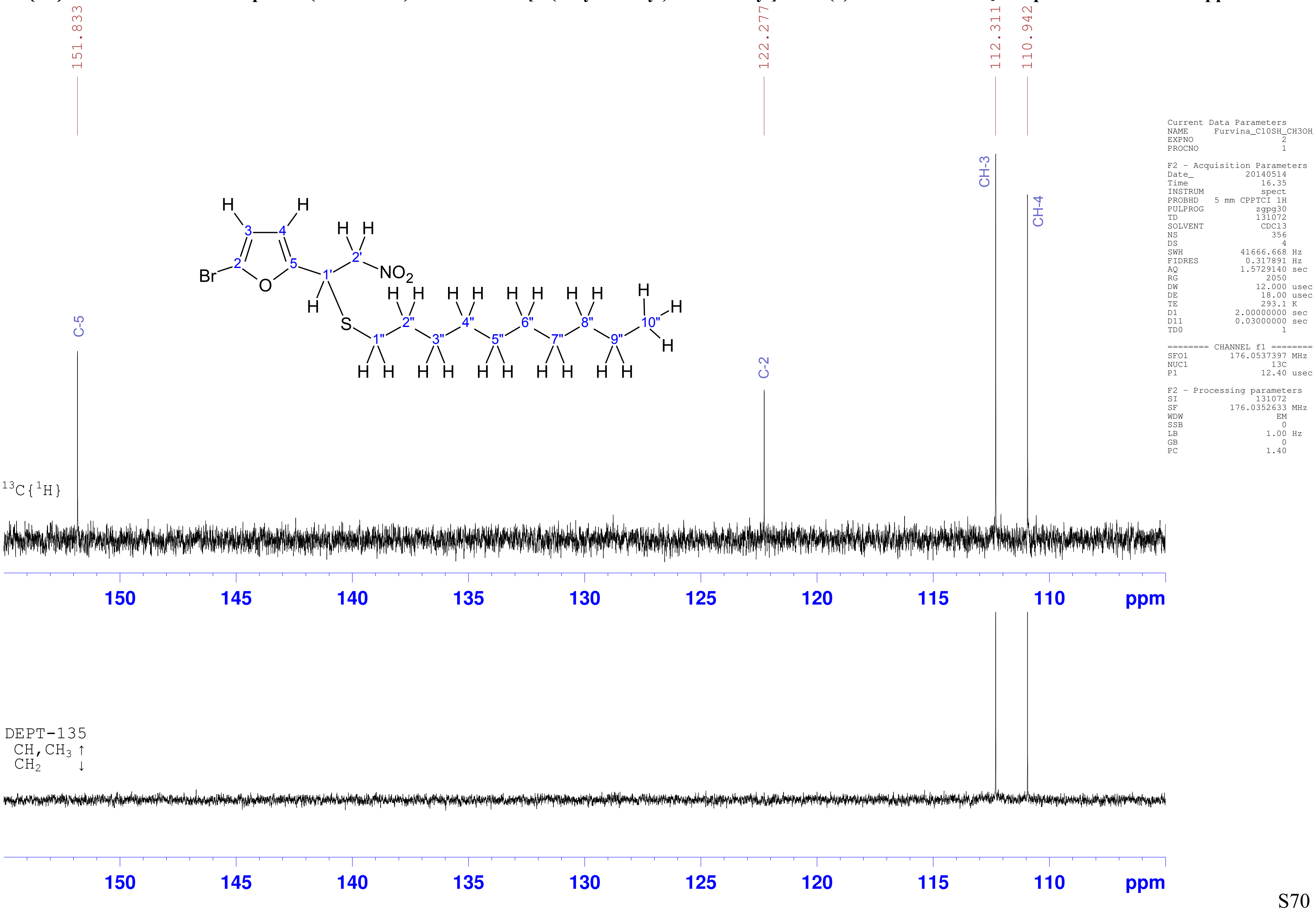

$^{13}C\{^{1}H\}$ and DEPT-135 NMR spectra (176.0 MHz) of 2-bromo-5-[1-(decylsulfanyl)-2-nitroethyl]furan (5) solution in $CDCl_3$ – expansion from +12.0 ppm to +81.0 ppm

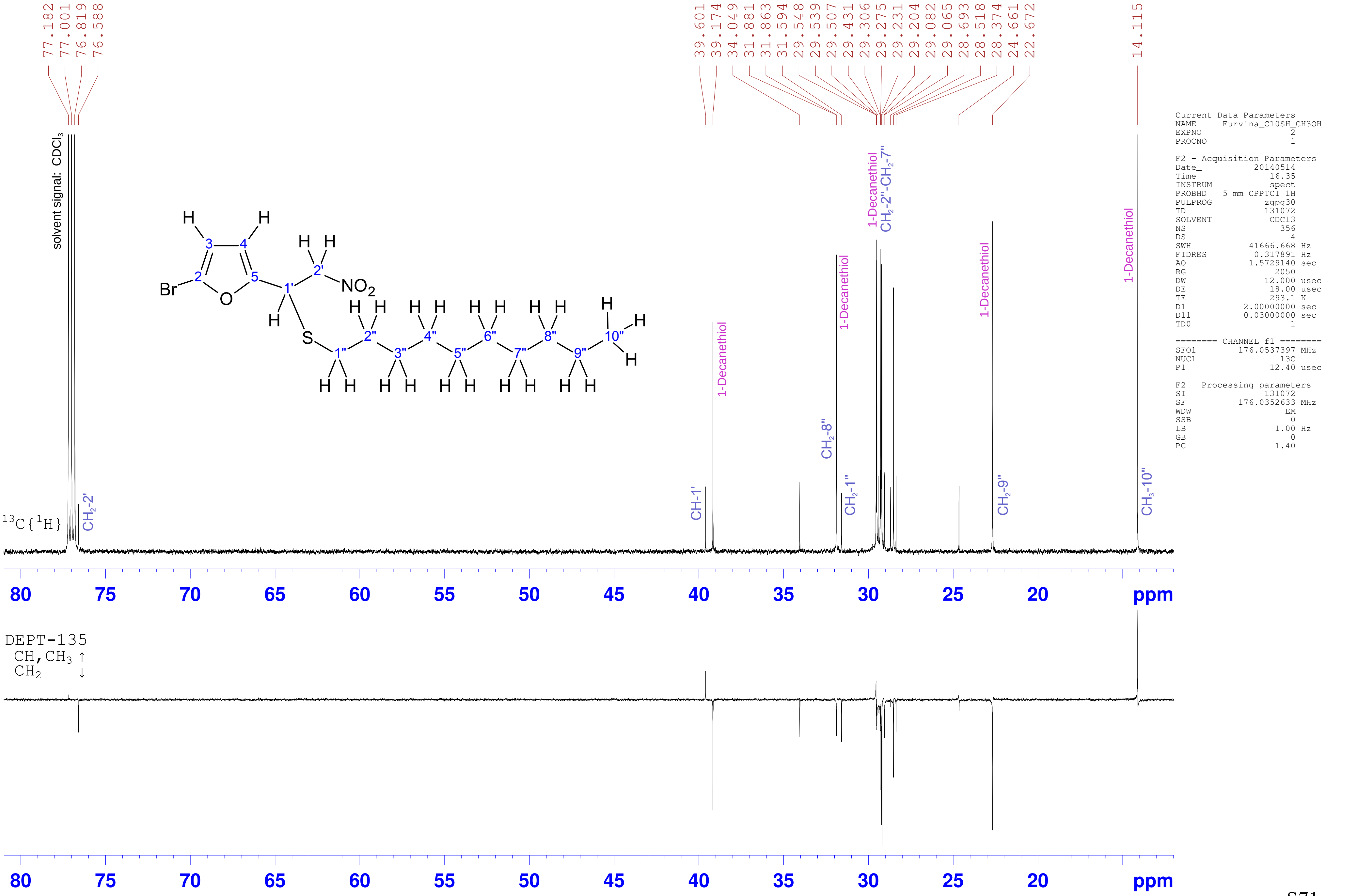

^{1}H-^{15}N HMBC NMR spectrum (700.1 MHz, 70.9 MHz) of 2-bromo-5-[1-(decylsulfanyl)-2-nitroethyl]furan (5) solution in $CDCl_3$

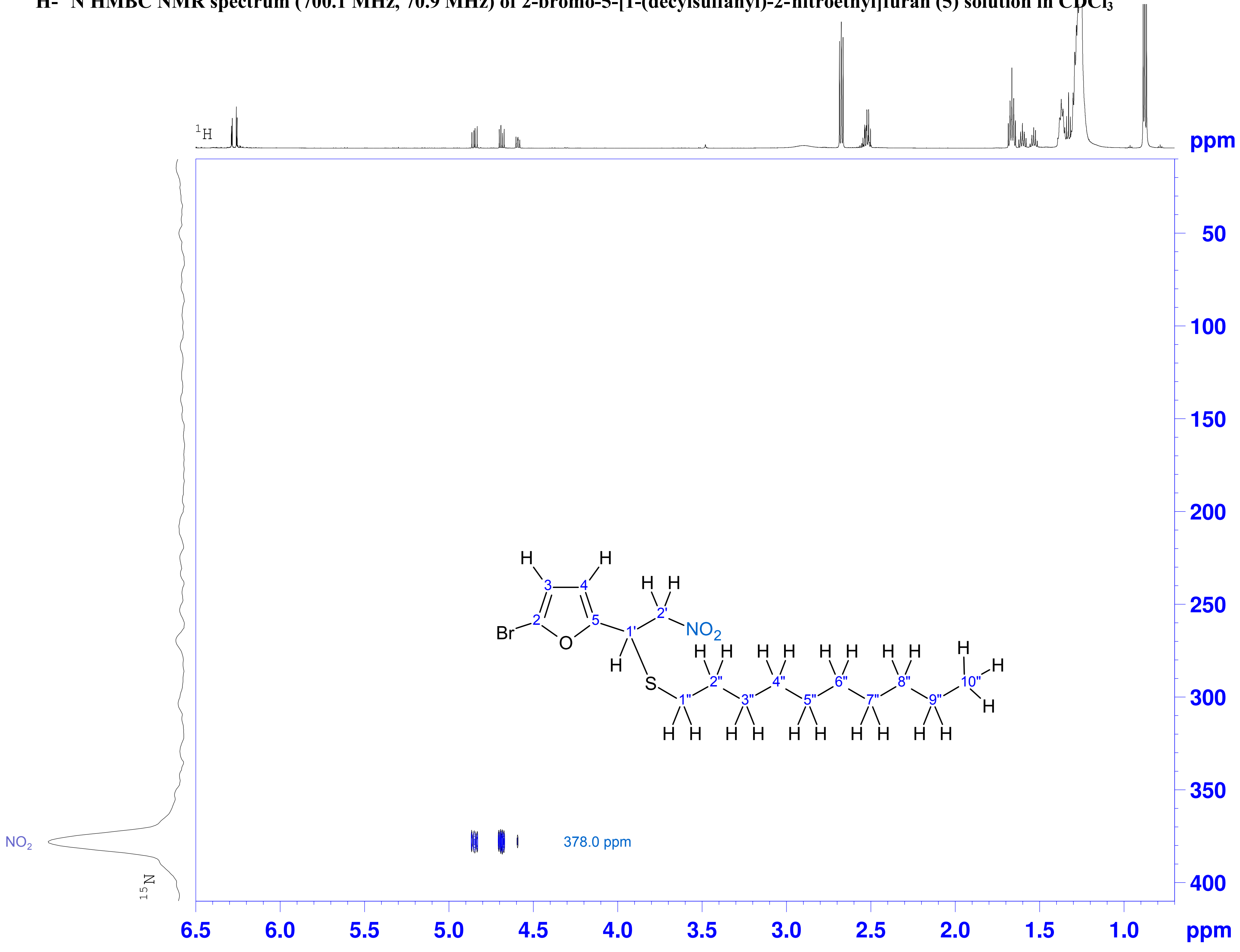


```
Current Data Parameters
NAME      Furvina_C10SH_C
EXPNO                   7
PROCNO                  1

F2 - Acquisition Parameters
Date_            20140514
Time                23.25
INSTRUM             spect
PROBHD   5 mm CPPTCI 1H
PULPROG    hmbcgplpndqf
TD                   4096
SOLVENT             CDCl3
NS                    256
DS                     16
SWH              5597.015 Hz
FIDRES           1.366459 Hz
AQ              0.3659593 sec
RG                   2050
DW                 89.333 usec
DE                  25.00 usec
TE                  293.2 K
CNST2          97.0000000
CNST13          7.0000000
D0             0.00000300 sec
D1             1.29999995 sec
D2             0.00515464 sec
D6             0.07142857 sec
D16            0.00020000 sec
IN0            0.00001760 sec

======== CHANNEL f1 ========
SFO1         700.0828003 MHz
NUC1                   1H
P1                   7.40 usec
P2                  14.80 usec

F1 - Acquisition parameters
ND0                     2
TD                     32
SFO1          70.95339 MHz
FIDRES      887.784119 Hz
SW             400.391 ppm
FnMODE                 QF

F2 - Processing parameters
SI                   4096
SF           700.0800185 MHz
WDW                 QSINE
SSB                     2
LB                   0.00 Hz
GB                      0
PC                   1.40

F1 - Processing parameters
SI                    512
MC2                    QF
SF            70.9384928 MHz
WDW                 QSINE
SSB                     2
LB                   0.00 Hz
GB                      0
```

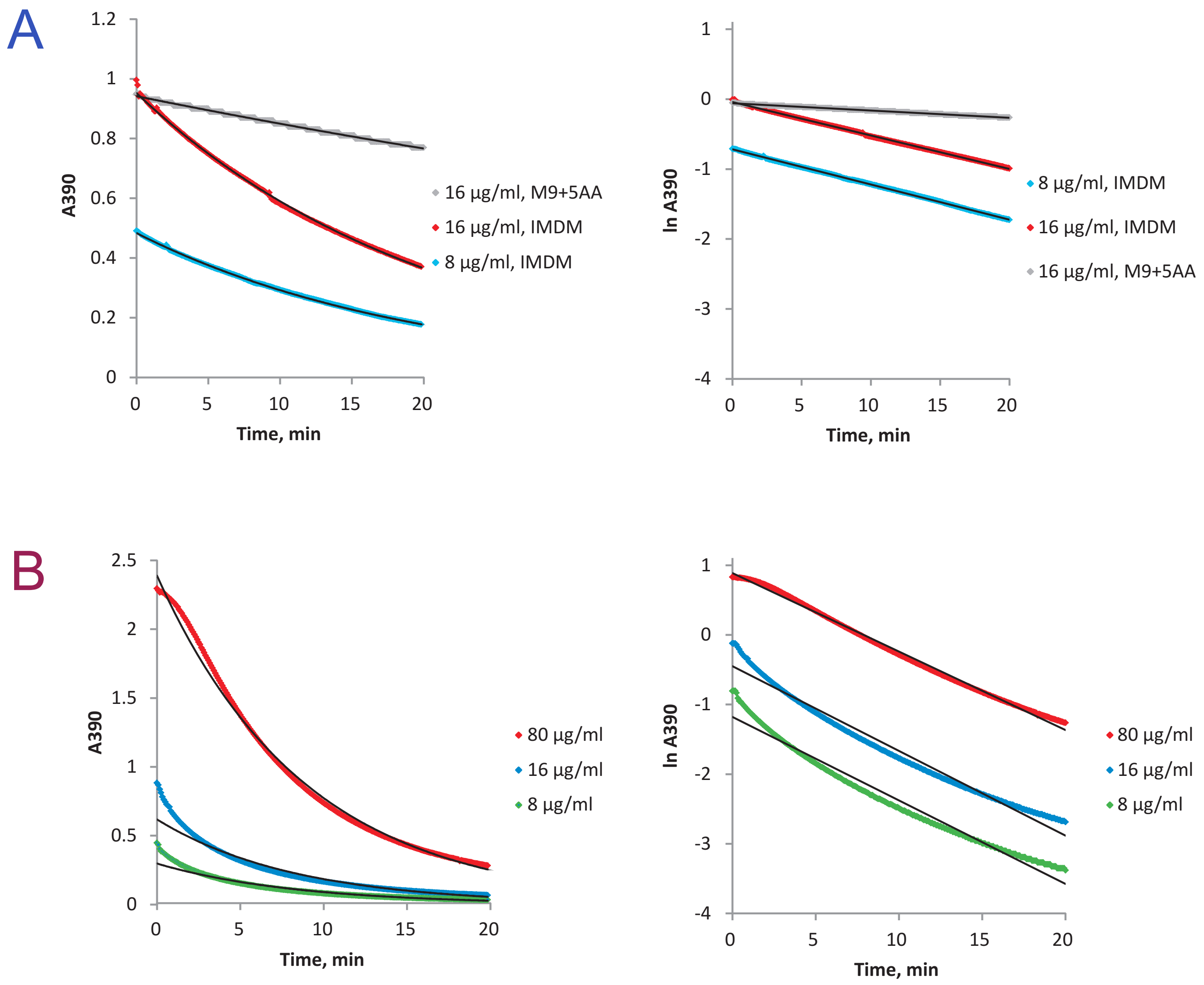

Figure S1. Conversion of G1 in cell culture medium IMDM (A) and bacterial complete medium CAMHB (B).
